# Novel Triterpenoid Alkaloids With Their Potential Cytotoxic Activity From the Roots of *Siraitia grosvenorii*

**DOI:** 10.3389/fchem.2022.885487

**Published:** 2022-04-29

**Authors:** Huijuan Wang, Guoxu Ma, Huaxiang Wang, Lingyu Li, Aijun Dong, Huiping Liu, Xiaoshuang Huo, Jianyong Si, Junchi Wang

**Affiliations:** The Key Laboratory of Bioactive Substances and Resources Utilization of Chinese Herbal Medicine, Ministry of Education, Institute of Medicinal Plant Development, Chinese Academy of Medical Sciences and Peking Union Medical College, Beijing, China

**Keywords:** Cucurbitaceae, *Siraitia grosvenorii*, cucurbitane-type triterpenoid, pyrazine, cytotoxicity

## Abstract

Four novel triterpenoid alkaloids, siragrosvenins A–D (**1–4**), and two new cucurbitane-type triterpenoids, siragrosvenins E–F (**5**, **6**), together with eight known analogs (**7−14**), were isolated from the roots of *Siraitia grosvenorii*. Compounds **1−4** possessed a rare cucurbitane-type triterpenoid scaffold, featuring an extra pyrazine unit *via* the Strecker reaction in the cucurbitane framework. Compound **5** displayed a 6/6/6/5/6/5-fused polycyclic ring system, with an uncommon fused furan and pyran ring in the side chain. All the structures were characterized by extensive spectroscopic analysis, including HRESIMS, NMR, and X-ray crystallographic data. It is worth noting that the DP4^+^ analysis method was applied for the first time to determine the absolute configurations of the trihydroxybutyl moiety in the side chain of compounds **1–4**. *In vitro* cytotoxicity screening found that compounds **4, 8, 9, 13,** and **14** exhibited remarkable cytotoxic activities against three cell lines with IC_50_ values ranging from 1.44 to 9.99 μM. Siragrosvenin D shows remarkable cytotoxic activity on MCF-7 cells. As a result, it inhibited the proliferation of MCF-7 cells and reduced their viability *via* the induction of G2/M phase arrest and significantly induced apoptosis in MCF-7 cells.

## Introduction

Triterpenoids are commonly distributed in higher plants and have attracted much attention due to their structural diversities and broad range of bioactivities (Chen et al., 2005). Cucurbitane triterpenoids, as an important part of the triterpenoid family, are famous for their highly oxygenated skeletons, which are obtained initially from the Cucurbitaceae genus. More importantly, members of this group of natural products have been reported for their diverse pharmacological effects, such as anticancer (Garg et al., 2018), anti-inflammatory (Liu et al., 2020), antihyperglycemic (Sun et al., 2018), and antilipidemic activities (Huang et al., 2012; Cai et al., 2015). In addition, cucurbitane triterpeniods possessing a nitrogen-containing heterocycle are rarely reported. The insertion of heteroatoms can often improve the biological activity of chemicals, which has attracted extensive attention of scholars (Pettit et al., 1988; Urban et al., 2007).

**FIGURE 1:**
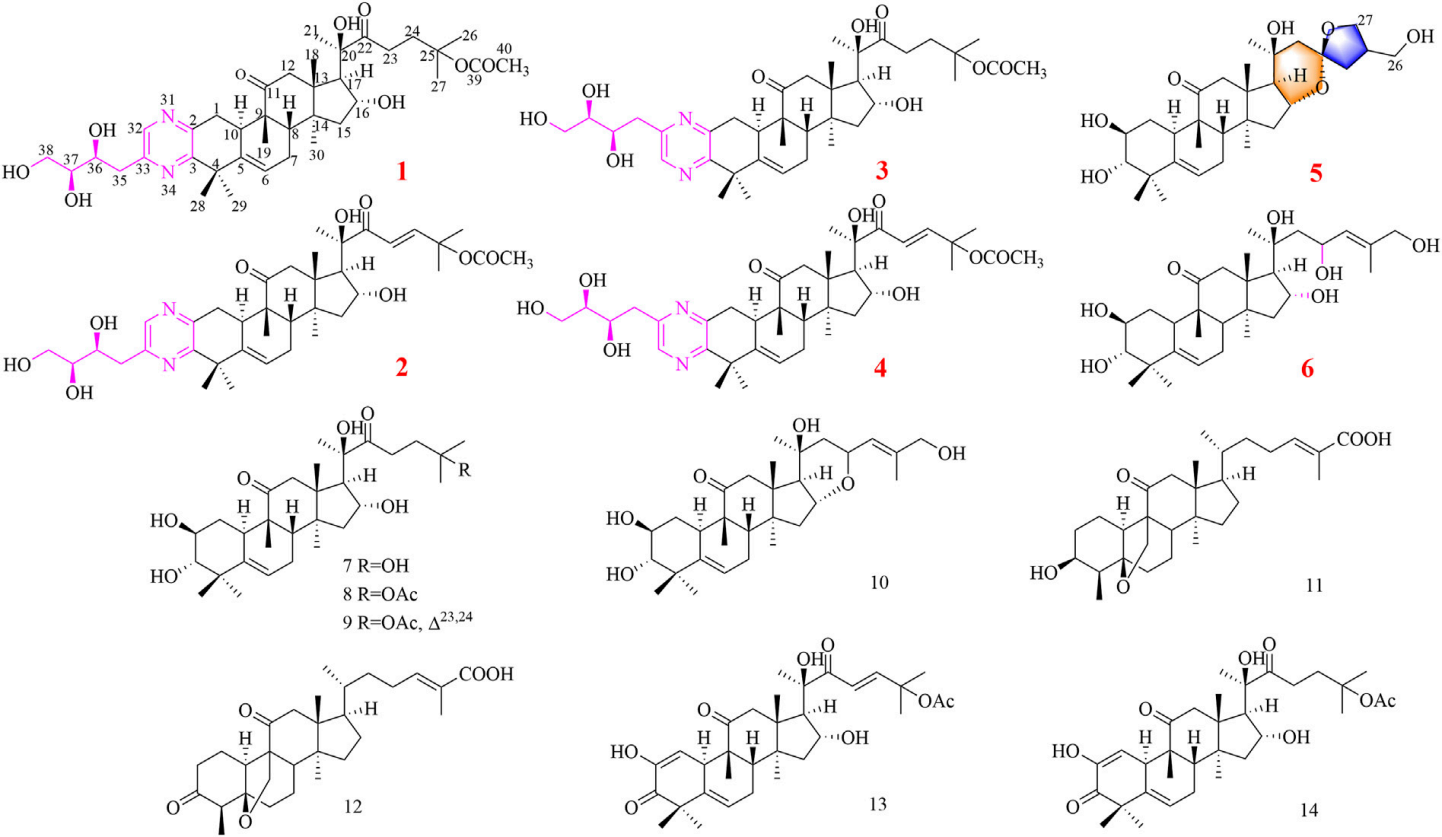
Compounds **1–14** isolated from *S. grosvenorii*.

*Siraitia grosvenorii* Swingle (Cucurbitaceae) is a perennial plant growing in the southern part of China, Guangxi province. The roots of *S. grosvenorii* are traditionally used to treat tongue fat, meningitis sequelae, diarrhea, and rheumatoid arthritis as a folk medicine in China (Qing et al., 2017). However, few investigations were conducted on the isolation and identification of compounds presented in the roots. In order to search for active natural products from this plant, we isolated four uncommon triterpenoid alkaloids (**1–4**), two new cucurbitane-type triterpenoids (**5**, **6**) and eight known compounds (**7–14**) (Figure 1). Among them, compounds **1–4** contained a novel cucurbitane-type triterpenoid skeleton with an additional pyrazine unit *via* a carbon–nitrogen linkage in the structure. Compound **5** showed an unexpected triterpenoid structure with a 6/6/6/5/6/5-fused polycyclic ring system, through aldol condensation. The cytotoxicity of these compounds was evaluated against three human cancer cell lines (MGC-803, MCF-7, and CNE-1) *in vitro*. Herein, we presented the isolation, structure elucidation, and cytotoxicity of these cucurbitane-type triterpenoids. Furthermore, we also conducted a preliminary investigation on the effects of siragrosvenin D, which could arrest the cell cycle and significantly induce apoptosis in MCF-7 cells.

## Materials and Methods

### Plant Material

The roots of *S. grosvenorii* were collected from the Yongfu county, Guangxi province and identified by Prof. Ma Xiaojun (Institute of Medicinal Plant Development, Chinese Academy of Medical Sciences and Peking Union Medical College) in October 2019. The voucher specimen (accession number 2019004) was stored at the herbarium of our institute.

### General Experimental Procedures

UV spectra data were recorded on a Thermo Scientific Genesys 10S spectrophotometer (Thermo Scientific, Madison, WI, United States). Infrared spectra were obtained on a Nicolet iS5 FT-IR spectrophotometer (Thermo Scientific, Madison, WI, United States). HRESIMS data were measured on a Thermo Scientific LTQ-Orbitrap XL (Bremen, Germany). Optical rotations were measured with an Anton Paar MCP 200 automatic polarimeter (Anton Paar GmbH, Graz, Austria) in MeOH at 25°C. 1D and 2D NMR spectra data were recorded on a Bruker AV III 600 NMR spectrometer (Rheinstetten, Germany). Column chromatography was performed by using silica gel (100–200, 300–400 mesh, Qingdao Haiyang Chemical Co., Ltd., Qingdao, China) and Sephadex LH-20 (Pharmacia Biotech, Sweden). Thin-layer chromatography (TLC) was performed over precoated silica gel GF_254_ plates (0.25 mm, Qingdao Haiyang Chemical Co., Ltd., Qingdao, China). Semi-preparative HPLC was carried out on a LC-UV system (Waters 2,549, United States) with a YMC-C_18_ column (250 × 10 mm, 5 μm, Japan) and Agilent SB-Phenyl (250 × 10 mm, 5 μm, United States), detected by a binary channel UV detector at 210 and 254 nm. All solvents used for HPLC were of HPLC grade obtained from Thermo Fisher.

### Extraction and Isolation

The air-dried roots (20.0 kg) of *S. grosvenorii* were extracted three times by 90% ethanol under reflux. The solvent was then removed under reduced pressure to yield a crude extract (1,300 g), which was then suspended in water (3 × 2000 ml) and partitioned with petroleum ether, dichloromethane, ethyl acetate, and n-butanol. The CH_2_Cl_2_ residue (200 g) was chromatographed on silica gel CC using an increasing gradient of CH_2_Cl_2_-MeOH (150:1 to 0:1, *v/v*) to afford seven main fractions (A−G).

Fraction B (30 g) was submitted to a silica gel column by using CH_2_Cl_2_-MeOH (120:1 to 0:1 *v/v*) as an eluent to generate fractions B_1_–B_5_. Subsequently, further purification of B_2_ was separated as four parts over an MCI gel column with a MeOH-H_2_O gradient system (7:3–0:1 *v/v*). B_2_-1 was purified by a Sephadex LH-20 column (4 × 120 cm) to yield compound **7** (10 mg). Compound **1** (3.0 mg, t_R_ 23 min) and compound **2** (8.0 mg, t_R_ 25 min) were isolated from fraction B_2_-3 (24 mg) *via* semi-preparative HPLC with 40% MeOH at 2 ml/min. B_2_-4 (33 mg) was chromatographed by semi-preparative HPLC (CH_3_CN-H_2_O, 65:35, *v/v*) to furnish compound **3** (3.2 mg, t_R_ 15 min), compound **4** (2.4 mg, t_R_ 17 min), and compound **8** (6 mg, t_R_ 22 min). B_2_-5 was subjected to an additional separation by preparative HPLC (MeOH-H_2_O, 55:45, *v/v*, 5 ml/min) to afford three parts, which were further purified by semi-preparative HPLC to yield compound **9** (20 mg, t_R_ 11.1 min), compound **11** (15 mg, t_R_ 12 min), and compound **5** (5 mg, t_R_ 15.3 min).

Fraction B_4_ (7 g) was applied to separation by the MCI gel column using MeOH–H_2_O with a stepwise elution gradient (7:3–0:1 *v/v*) to yield eight parts. The 60% MeOH fraction (B_4_-3, 2.5 g) was fractionated on silica gel columns to obtain eight fractions. Fraction B_4_-3-3 (30 mg) was chromatographed by semi-preparative HPLC (MeOH-H_2_O, 45:55) to furnish compound **10** (4.5 mg, t_R_ 24 min). Fraction B_4_-4 was subjected to Sephadex LH-20 (4 × 120 cm). We merged the same fractions according to their TLC profiles and then acquired compound **6** (6 mg, t_R_ 19 min), **12** (5 mg, t_R_ 28 min), and **13** (5 mg, t_R_ 30 min) from fraction B_4_-4 using semi-preparative HPLC (CH_3_CN -H_2_O, 70:30). B_4_-5 was separated by semi-preparative HPLC (20% MeOH–80% H_2_O) to yield **14** (4 mg, t_R_ 15.5 min).

### Compound Characterization

*Siragrosvenin A (* ***1*** *)*: pale-yellow powder; (α)^25^_D_ +43 (c 0.1, MeOH); UV (MeOH) *λ*_max_ (log ε) 205 (1.19) and 282 (0.90) nm; IR *ν*_max_ 3,419, 2,968, 2,928, 1,697, 1,392, 1,370, 1,260, and 1,205 cm^−1^; ^1^H NMR (Pyrdine-D_5_, 600 MHz) and ^13^C NMR (Pyrdine-D_5_, 150 MHz) data are shown in Tables 1, 2; HRESIMS (positive mode) *m/z* 707.3880 (M + Na)^+^ (calculated for C_38_H_56_N_2_O_9_Na, 707.3883).

**TABLE 1:** ^1^H (600 MHz) NMR data of compounds **1–6** in pyrdine-d_5_.

No.	1	2	3	4	5	6
1	3.22, overlap	3.22, overlap	3.28, dd (6, 12)	3.27, dd (6, 12)	2.50, m	2.52, m
	2.74, overlap	2.72, overlap	2.82, overlap	2.82, overlap	1.54, m	1.56, m
2	—	—	—	—	4.09, m	4.12, m
3	—	—	—	—	3.43, m	3.45, d (9)
4	—	—	—	—	—	—
5	—	—	—	—	—	—
6	5.92, m	5.91, m	5.90, m	5.89, m	5.69, m	5.72, m
7	2.36, m	2.38, m	2.37, m	2.37, m	2.33, m	2.33, m
	1.95, m	1.97, m	1.94, m	1.93, m	1.95, m	1.91, m
8	2.01, m	2.00, m	1.97, m	1.98, m	1.97, m	2.01, m
9	—	—	—	—	—	—
10	2.83, m	2.84, m	2.75, m	2.75, m	2.66, m	2.71, m
11	—	—	—	—	—	—
12	3.22, overlap	3.20, d (12)	3.20, d (12)	3.20, d (12)	3.16, d (12)	3.21, d (12)
	2.88, d (12)	2.92, d (12)	2.88, d (12)	2.91, d (12)	2.67, overlap	2.71, d (12)
13	—	—	—	—	—	—
14	—	—	—	—	—	—
15	1.94, m	1.98, m	1.92, m	1.94, m	1.95, m	1.69, m
—	1.73, m	1.76, m	1.72, m	1.75, m	1.65, m	1.98, m
16	4.96, m	5.10, m	4.96, m	5.11, m	5.00, m	4.92, m
17	2.93, d (7.2)	3.00, d (7.2)	2.93, d (7.2)	2.98, d (7.2)	2.10, d (12)	2.18, d (12)
18	1.27, s	1.27, s	1.27, s	1.26, s	1.22, s	1.29, s
19	1.25, s	1.25, s	1.24, s	1.23, s	1.24, s	1.27, s
20	—	—	—	—	—	—
21	1.68, s	1.70, s	1.64, s	1.71, s	1.36, s	1.49, overlap
22	—	—	—	—	1.94, overlap	2.70, m
—	—	—	—	—	1.80, d (15.8)	1.70, m
23	3.33, m	7.34, d (15.8)	3.34, m	7.35, d (15.8)	—	5.15, m
—	3.10, m	—	3.12, m	—	—	—
24	2.45, m	7.41, d (15.8)	2.45, m	7.41, d (15.8)	2.33, m	6.06, m
—	2.37, m	—	2.36, m	—	1.71, m	—
25	—	—	—	—	2.88, m	—
26	1.50, s	1.55, s	1.51, s	1.55, s	3.79, m	4.30, s
—	—	—	—	—	3.74, m	—
27	1.46, s	1.52, s	1.48, s	1.52, s	4.22, m	1.79, s
—	—	—	—	—	4.09, m	—
28	1.63, s	1.68, s	1.62, s	1.61, s	1.47, s	1.49, overlap
29	1.47, s	1.46, s	1.43, s	1.49, s	1.31, s	1.32, s
30	1.48, s	1.53, s	1.49, s	1.47, s	1.34, s	1.39, s
32	8.78, s	8.76, s	—	—	—	—
33	—	—	8.64, s	8.64, s	—	—
35	3.82, dd	3.82, dd	3.79, dd	3.79, dd	—	—
—	(2.6, 14)	(2.6, 14)	(2.6, 14)	(2.6, 14)	—	—
—	3.38, dd	3.38, dd	3.42, dd	3.42, dd	—	—
—	(9.8, 14)	(9.8, 14)	(9.8, 14)	(9.8, 14)	—	—
36	4.36, m	4.36, m	4.33, m	4.33, m	—	—
37	4.83, m	4.84, m	4.80, m	4.80, m	—	—
38	4.52, m	4.52, m	4.52, m	4.51, m	—	—
—	4.40, m	4.40, m	4.38, m	4.40, m	—	—
39	—	—	—	—	—	—
40	1.90, s	1.88, s	1.90, s	1.88, s	—	—

**TABLE 2:** ^13^C (150 MHz) NMR data of compounds **1–6** in pyrdine-d_5_.

No.	1	2	3	4	5	6
1	33.3, CH_2_	33.3, CH_2_	32.9, CH_2_	33.0, CH_2_	35.0, CH_2_	35.1, CH_2_
2	151.4, C	151.4, C	149.9, C	149.8, C	71.3, CH	71.3, CH
3	155.7, C	155.7, C	157.3, C	157.3, C	81.8, CH	81.8, CH
4	42.7, C	42.7, C	42.9, C	42.9, C	43.2, C	43.2, C
5	142.1, C	142.1, C	142.0, C	142.0, C	142.9, C	142.8, C
6	121.0, CH	121.0, CH	121.1, CH	121.1, CH	119.0, CH	119.0, CH
7	24.5, CH_2_	24.5, CH_2_	24.5, CH_2_	24.5, CH_2_	24.6, CH_2_	24.6, CH_2_
8	43.3, CH	43.4, CH	43.2, CH	43.4, CH	43.1, CH	43.2, CH
9	49.1, C	49.1, C	49.1, C	49.1, C	49.4, C	49.6, C
10	36.3, CH	36.2, CH	36.6, CH	36.5, CH	34.5, CH	34.5, CH
11	213.7, C	213.8, C	213.6, C	213.7, C	213.2, C	213.5, C
12	49.7, CH_2_	49.6, CH_2_	49.7, CH_2_	49.6, CH_2_	49.1, CH_2_	49.2, CH_2_
13	51.3, C	51.4, C	51.3, C	51.4, C	48.5, C	49.1, C
14	49.1, C	49.0, C	49.1, C	49.0, C	49.6, C	48.9, C
15	46.7, CH_2_	46.8, CH_2_	46.7, CH_2_	46.8, CH_2_	41.0, CH_2_	41.8, CH_2_
16	70.7, CH	71.0, CH	70.7, CH	71.0, CH	70.8, CH	77.0, CH
17	59.4, CH	60.2, CH	59.4, CH	60.2, CH	55.4, CH	56.3, CH
18	20.2, CH_3_	20.3, CH_3_	20.2, CH_3_	20.2, CH_3_	20.2, CH_3_	21.0, CH_3_
19	20.8, CH_3_	21.0, CH_3_	20.8, CH_3_	21.0, CH_3_	21.0, CH_3_	20.6, CH_3_
20	80.5, C	80.2, C	81.9, C	80.2, C	72.6, C	71.8, C
21	25.9, CH_3_	26.0, CH_3_	25.9, CH_3_	26.0, CH_3_	29.0, CH_3_	25.8, CH_3_
22	215.4, C	204.7, C	215.5, C	204.8, C	49.6, CH_2_	50.0, CH_2_
23	32.6, CH_2_	122.8, CH	32.6, CH_2_	122.8, CH	110.0, C	73.7, CH
24	35.7, CH_2_	150.2^a^, CH	35.8, CH_2_	150.2^a^, CH	42.2, CH_2_	126.2, CH
25	81.9, C	80.0, C	80.5, C	80.0, C	40.6, CH	138.5, C
26	26.3, CH_3_	26.5, CH_3_	26.3, CH_3_	26.5, CH_3_	64.8, CH_2_	67.6, CH_2_
27	26.4, CH_3_	26.9, CH_3_	26.4, CH_3_	26.9, CH_3_	71.1, CH_2_	14.7, CH_3_
28	32.0, CH_3_	32.1, CH_3_	31.6, CH_3_	31.7, CH_3_	25.8, CH_3_	30.0, CH_3_
29	30.7, CH_3_	30.7, CH_3_	30.9, CH_3_	30.8, CH_3_	22.6, CH_3_	22.7, CH_3_
30	19.3, CH_3_	19.3, CH_3_	19.3, CH_3_	19.3, CH_3_	21.7, CH_3_	21.6, CH_3_
32	144.2, CH	144.2, CH	143.3, C	143.2, C	—	—
33	153.8, C	153.8, C	154.6, CH	154.5, CH	—	—
35	40.4, CH_2_	40.4, CH_2_	40.4, CH_2_	40.4, CH_2_	—	—
36	73.5, CH	73.5, CH	73.6, CH	73.6, CH	—	—
37	76.8, CH	76.8, CH	76.7, CH	76.7, CH	—	—
38	65.5, CH_2_	65.5, CH_2_	65.4, CH_2_	65.4, CH_2_	—	—
39	170.5, C	170.2, C	170.5, C	170.2, C	—	—
40	22.6, CH_3_	22.1, CH_3_	22.6, CH_3_	22.1, CH_3_	—	—

*Siragrosvenin B (* ***2*** *)*: pale-yellow powder; (α)^25^_D_ +25 (c 0.1, MeOH); UV (MeOH) *λ*_max_ (log ε) 205 (1.32) and 282 (1.01) nm; IR *ν*_max_ 3,444, 2,976, 2,942, 1,698, 1,369, 1,254, and 1,022 cm^−1^; ^1^H NMR (Pyrdine-D_5_, 600 MHz) and ^13^C NMR (Pyrdine-D_5_, 150 MHz) data are shown in Tables 1, 2; HRESIMS (positive mode) *m/z* 705.3724 (M + Na)^+^ (calculated for C_38_H_54_N_2_O_9_Na, 705.3727).

*Siragrosvenin C (* ***3*** *)*: pale-yellow powder; (α)^25^_D_ +5 (c 0.1, MeOH); UV (MeOH) *λ*_max_ (log ε) 207 (2.09) and 282 (1.35) nm; IR *ν*_max_ 3,420, 2,966, 2,928, 1,697, 1,388, 1,371, 1,261, and 1,024 cm^−1^ ^1^H NMR (Pyrdine-D_5_, 600 MHz) and ^13^C NMR (Pyrdine-D_5_, 150 MHz) data are shown in Tables 1, 2; HRESIMS (positive mode) *m/z* 707.3882(M + Na)^+^ (calculated for C_38_H_56_N_2_O_9_Na, 707.3883).

*Siragrosvenin D (* ***4*** *)*: pale-yellow powder; (α)^25^_D_ +9 (c 0.1, MeOH); UV (MeOH) *λ*_max_ (log ε) 207 (2.35) and 282 (0.90) nm; IR *ν*_max_ 3,419, 2,967, 2,927, 1,687, 1,372, 1,260, and 1,023 cm^−1^; ^1^H NMR (Pyrdine-D_5_, 600 MHz) and ^13^C NMR (Pyrdine-D_5_, 150 MHz) data are shown in Tables 1, 2; HRESIMS (positive mode) *m/z* 705.3730 (M + Na)^+^ (calculated for C_38_H_54_N_2_O_9_Na, 705.3727).

*Siragrosvenin E (* ***5*** *)*: white powder; (α)^25^_D_ +159 (c 0.1, MeOH); UV (MeOH) *λ*_max_ (log ε) 207 (2.13) nm; IR *ν*_max_ 3,419, 2,965, 2,882, 1,688, 1,055, and 1,029 cm^−1^; ^1^H NMR (600 MHz, Pyrdine-D_5_) and ^13^C NMR (150 MHz, Pyrdine-D_5_) data are shown in Tables 1, 2; HRESIMS (positive mode) *m/z* 541.3126 (M + Na)^+^ (calculated for C_30_H_46_O_7_Na, 541.3141).

*Siragrosvenin F* (**6**): white powder; (α)^25^_D_ +59 (c 0.1, MeOH); UV (MeOH) *λ*_max_ (log ε) 228 (2.60) nm; IR *ν*_max_ 3,391, 2,966, 2,882, 1,687, 1,373, 1,068, and 1,027 cm^−1^; ^1^H NMR (600 MHz, Pyrdine-D_5_) and ^13^C NMR (150 MHz, Pyrdine-D_5_) data are shown in Tables 1, 2; HRESIMS (positive mode) *m/z* 543.3301 (M + Na)^+^ (calculated for C_30_H_48_O_7_Na, 543.3297).

### ^13^C NMR Calculations

The ^13^C NMR spectra were calculated according to the reported methods (Buevich and Elyashberg, 2016). The computational data were fitted in the GraphPad Prism 7. The process is also described in detail in Supplementary Material.

### X-Ray Crystallographic Analysis

Colorless needle crystals of compound **5** were crystallized in CH_3_CN–H_2_O (5:1) at room temperature. Crystal data: C_30_H_46_O_7_, hexagonal, a = 22.7327 (2) Å, b = 22.7327 (2) Å, c = 9.80650 (10) Å, *β* = 90°, U = 4,388.81 (9) Å3, T = 99.99 (10), space group P6_1_, Z = 6, μ (Cu Kα) = 1.54184, and Flack parameter = -0.03 (4). A total of 49,416 reflections were measured, 6,100 unique (R_int_ = 0.0394) which were used in all calculations. The final R_1_ was 0.0331 (I > 2σ (I)), and the wR_2_ was 0.0916 (all data). Crystal size: 0.32 × 0.06 × 0.05 mm^3^.

The crystallographic data have been deposited in the Cambridge Crystallographic Data Center (CCDC), and the CCDC deposition number is CCDC 2151134. These data can be obtained free of charge *via* http://www.ccdc.cam.ac.uk/conts/retrieving.html.

### Cell Viability Assays

The cellular viability of compounds **1–14** was evaluated using the MTT procedure with MGC-803, MCF-7, and CNE-1 cancer cell lines. The cells were cultured in DMEM supplemented with 10% fetal bovine serum and cultured at a density of 3 × 10^4^ cells/mL in a 96-well microtiter plate. After 24 h of incubation, six various concentrations of each agent dissolved in dimethyl sulfoxide (DMSO) were then added in the wells. Each concentration was evaluated three times. After incubation under 5% CO_2_ at 37°C for 48 h, 20 μL of MTT (5 mg/ml) was added into each well, and the cells were incubated for an additional 4 h. Then, after the liquid was removed, DMSO (200 μL) was placed into the wells. After shaking for 5 min, the absorbance was measured with a microplate reader at 570 nm (SpectraFluor, TECAN, Sunrise, Austria).

### Cell Cycle Analysis

The MCF-7 cells were plated in a six-well plate at a density of 3 × 10^4^ cells per well and treated with compound **4** (0, 5, 10, and 20 μM). After 24 h, the cells were fixed in ice-cold ethanol (70%) at 4°C overnight. After the cells were suspended in 0.1% Triton X-100 and 100 μg/ml RNase A, 5 μL PI solution was added and incubated for 30 min (Ueno et al., 2021). Then, the sample was analyzed by a flow cytometer FACS Verse. Modifit LT 4.0 was used to analyze the obtained data.

### Apoptosis Analysis

The MCF-7 cells were pre-treated with compound **4** (0, 5, 10, and 20 μM) for 24 h. After washing with PBS, the cells were incubated with 5 μL annexin V in binding buffer for 30 min at room temperature in the dark, followed by 10 μL PI for 5 min. FACS Calibur flow cytometry (Becton Dickinson, United States) was used to detect and analyze the stained cells. The apoptosis rate was reported as the percentage of apoptotic cells to the total number of cells.

### Colony Formation Assay

The MCF-7 cells were plated in a six-well plate at a density of 250 cells per well and were treated with different concentrations of compound **4** (0, 0.1, 0.5, 1, 2, and 4 μM) for 14 days. The drugs were removed, and cells were washed twice with PBS. Then, the cells were fixed in methanol for 10 min and stained with 0.1% crystal violet solution for 30 min at room temperature. Finally, PBS was used to wash the cells to visualize the colonies (Ni et al., 2018).

### Cell Morphology Observation and AO/EB Staining Assay

The MCF-7 cells were seeded at a density of 2 × 10^4^ cells/well onto 24-well plates and were treated with different concentrations of compound **4** (0, 5, 10, and 20 μM) for 24 h. After discarding the cell culture medium, some cells were added with 500 μL AO/EB staining solution for 5 min in the dark. Subsequently, photographs were taken under a fluorescence microscope.

### Statistical Analysis

All data were analyzed by GraphPad Prism version 5.0 and were presented as the mean ± SD in at least three independent experiments. Student’s *t*-test and one way ANOVA were conducted to evaluate significant distinctions. Values of *p* < 0.05 were considered as statistically significant.

## Results and Discussion

### Compound Structure Elucidation

**FIGURE 2:**
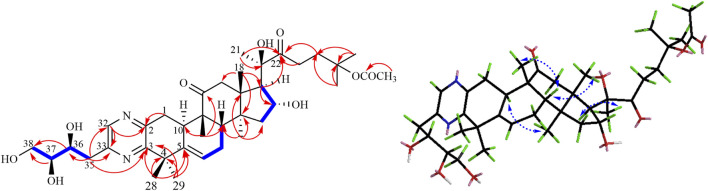
Key ^1^H-^1^H COZY, HMBC, and NOESY correlations of compound **1**.

Compound **1** was isolated as a pale-yellow powder. Its molecular formula C_38_H_56_N_2_O_9_ was indicated by its HRESIMS at *m/z* 707.3880 (M + Na)^+^, calculated for C_38_H_56_N_2_O_9_Na 707.3883, suggesting 12 degrees of unsaturation. The absorption bands at 3,419 and 1,697 cm^−1^ in the IR spectrum suggested the existence of the hydroxyl and carbonyl groups in compound **1**. The characteristic absorption bands at 205 and 280 nm in the UV spectrum provided evidence for the existence of the aromatic group. The ^1^H NMR spectrum (Table 1) displayed signals attributed to nine methyl groups at δ_H_ 1.25, 1.27, 1.46, 1.47, 1.48, 1.50, 1.63, 1.68, and 1.90 (each 3H, s); a set of oxygenated proton signals at δ_H_ 4.96 (1H, t), 4.83 (1H, t), 4.52 (1H, m), 4.40 (1H, m), and 4.36 (1H, m) suggested its polyhydroxy property. One olefinic proton at δ_H_ 5.92 (1H, m) indicated the presence of double bonds, and one downfield proton at δ_H_ 8.78 (1H, s) implied the heterocyclic aromatic ring. The ^13^C-APT NMR spectrum (Table 1) revealed 38 carbon resonances, including two keto carbonyls at δ_C_ 213.7 and 215.4, one ester carbonyl at δ_C_ 170.5, and six olefinic carbons and aromatic carbons at δ_C_ 155.7, 153.8, 151.4, 144.2, 142.1, and 121.0. The rest of the signals were ascribed to nine methyls, eight methylenes (including one oxygenated carbon at δ_C_ 65.5), five methines (including three oxygenated carbons at δ_C_ 76.8, 73.5, and 70.7), and six quaternary carbons (including two oxygenated carbons at δ_C_ 80.5 and 81.9). All protons are assigned to their corresponding carbons with the help of the HSQC spectrum. In fact, the results after the comparison of these data with the known ones suggested that compound **1** showed the same B–C–D ring and the C-17 side chain structure as 23,24-dihydrocucurbitacin E (Wu et al., 2004), except for the additional four aromatic carbons (δ_C_ 155.7, 153.8, 151.4, and 144.2) and a trihydroxybutyl side chain. In the HMBC spectrum, the correlations from δ_H_ 8.78 (1H, s) to C-2 (δ_C_ 151.4) and C-33 (δ_C_ 153.8) implied that the four downfield aromatic carbons formed a closed-loop system. Considering the two N atoms in the molecular formula, an extra pyrazine unit was established in the structure (Seeman et al., 1992). The HMBC correlations (Figure 1) from H_3_-28, H_3_-29 to C-3 (δ_C_ 155.7) and C-5 and from H-1 to C-2 (δ_C_ 151.4) revealed that the pyrazine ring was adjacent to ring A. The presence of 36,37,38-trihydroxybutyl moiety was established by the ^1^H−^1^H COZY (Figure 2) correlations of H-36/H-37/H-38 and the key HMBC correlations of H-35 to C-36 and C-37, H-36 to C-35 and C-37, and H-38 to C-36 and C-37 (Wang et al., 2014). Subsequently, the trihydroxybutyl moiety was confirmed to attach to C-33 from the HMBC correlations of H-35 to C-33, C-32, and C-3 and H-32 to C-1, C-2, and C-3. Thus, the planar structure of **1** was established unambiguously.

**FIGURE 3:**
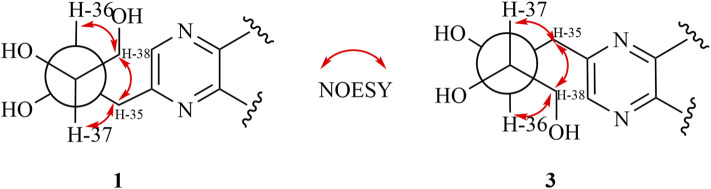
Threo-configuration of compound **1**.

**FIGURE 4:**
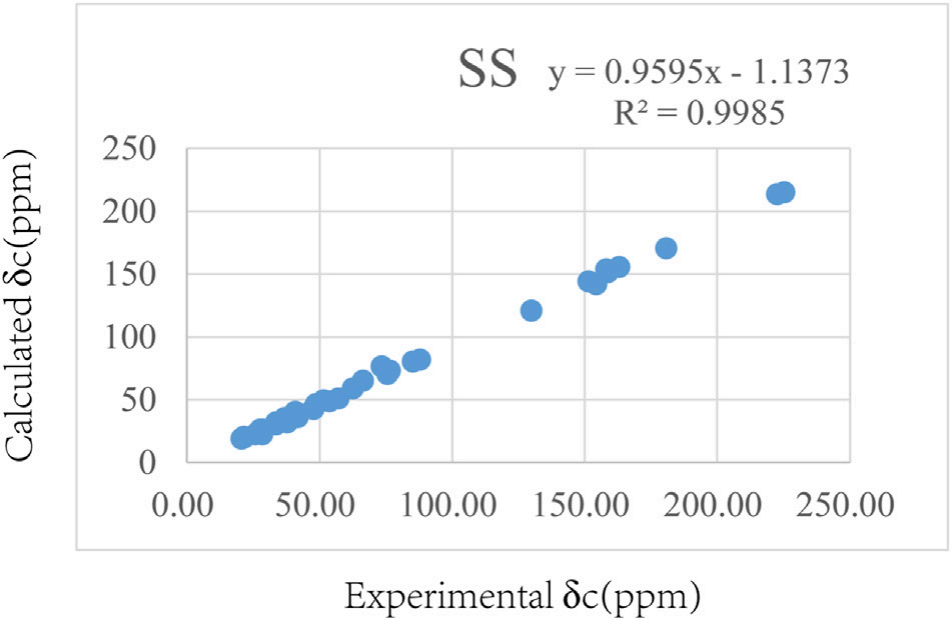
Regression analysis of the experimental and calculated ^13^C chemical shifts for **1**.

The relative configuration of **1** was evaluated by its NOESY spectrum (recorded in Pyrdine-d_5_). The NOESY correlations (Figure 2) from H_3_-19 to H_3_-18, H_3_-19 to H-8, H_3_-30 to H-10, H_3_-30 to H-17, and H_3_-18 to H-16 revealed that H_3_-18, H_3_-19, H-8, and H-16 are β-oriented, H_3_-30 and H-10, and H-17 are α-oriented. We tried to determine the absolute configuration of compound **1** by crystallization but failed. However, the clear comparison of NMR data with cucurbitacin E, whose absolute configuration has been fully determined, indicated that the cucurbitane partial structure at C-8, C-9, C-10, C-13, C-14, C-16, and C-17 of compound **1** was *S*, *R*, *R*, *R*, *S*, *R*, and *R*, respectively (Wu et al., 2004), which was completely consistent with the biogenic pathway of cucurbitane triterpenes. The relative configuration of the 36,37,38-trihydroxybutyl moiety was determined by *J*-based NMR. The low-temperature NMR (−4°C, CD_3_OD) of **1** revealed a large coupling constant between H-36 and H-37 (*J* = 9.2 Hz) (Wang et al., 2014), indicating an antirelationship between the two protons (Li et al., 2015). In addition, a significant NOESY correlation of H-37/H-35/H-38/H-36 indicated a threo configuration (Figure 3) of H-36 and H-37 (36*S*, 37*S* or 36*R*, 37*R*). The absolute configuration of this fragment was further determined by DP4 calculations (Marcarino et al., 2021). NMR shielding constants were computed using the GIAO method at the mPW1PW91/6–311 + G** level in the gas phase by the GAUSSIAN09 program (Zanardi et al., 2018). In the application of the DP4+ analysis method, we selected the only partial data of the 36,37,38-trihydroxybutyl moiety to carry out DP4 calculations of the two possible configurations (36*S*, 37*S* and 36*R*, 37*R*). Based on the calculation results, compound **1a** (36*S*, 37*S*) showed satisfying linear regression analysis (*R* ^2^ 0.9985) of the experimental data and calculated ^13^C chemical shifts (Figure 4), indicated better fit by comparison of experimental and calculated NMR data (Table 3), and then was designated as the most promising candidate. Therefore, the structure of compound **1** was assigned as shown in Figure 2.

**TABLE 3:** Comparison of calculated ^13^C NMR chemical shifts with experimentally observed shifts for **1**.

	DP4+ (%)	All data	0.00%	100.00%
Type	No.	Exp	1-RR	1-SS
C	33	153.82	162.2954822	158.159521
CH_2_	35	40.38	39.14271042	40.7792142
CH	36	73.52	79.24594056	73.4682572
CH	37	76.83	76.85050502	76.440967
CH_2_	38	65.46	63.22609582	66.2968835

Compound **2**, pale-yellow powder, had the molecular formula of C_38_H_54_N_2_O_9_ based on its HRESIMS data [*m/z* 705.3724 (M + Na)^+^, calculated for C_38_H_54_N_2_O_9_Na 705.3727], suggesting 13 degrees of unsaturation. The ^1^H NMR and ^13^C-APT NMR spectra data (Table 1) showed resemblance with those of **1**, except that compound **2** possessed an additional double bond at δ_C_ 122.8 and 150.2. In HMBC, the appearance of α–β unsaturated ketone at C-22–C-23 was further verified by the cross-peaks of H-23 (δ_H_ 7.34, 1H, d, *J* = 15.8 Hz), H-24 (δ_H_ 7.41, 1H, d, *J* = 15.8 Hz) to C-22 (δ_C_ 204.7) and correlations of the signals at H_3_-26 (δ_H_, 1.55) and H_3_-27 (δ_H_, 1.52) with the resonances at C-24 (δ_C_ 150.2). Additional NOESY correlations of H_3_-19/H_3_-18, H_3_-19/H-8, H_3_-30/H-10, H_3_-30/H-17, and H_3_-18/H-16 indicated that H_3_-18, H_3_-19, H-8, and H-16 are β-oriented, while H_3_-30, H-10, and H-17 are α-oriented. The similar absorption peaks (205 nm, Δε 9.0; 300 nm, Δε 1.2) in the CD spectrum of compound **2** indicated that the absolute configurations of compound **2** was identical with those of **1**. Thus, the structure of **2** was established as described.

Compound **3** was isolated as pale-yellow powder and assigned an identical molecular formula of C_38_H_56_N_2_O_9_ similar to that of **1** based on the HRESIMS ion at *m/z* 707.3880 (calculated for C_38_H_56_N_2_O_9_Na 707.3883). Extensive analysis of its NMR data indicated that the structure of **3** was similar to that of **1**, except for the downfield shifts of C-33 (Δδ + 0.8) and C-3 (Δδ + 1.6) and upfield shifts of C-32 (Δδ−0.9) and C-2 (Δδ−1.5), which indicated that the location of the trihydroxybutyl group may be different. By the HMBC experiment, the cross-peaks from H-35 (δ_H_ 3.79, 3.42) to C-32 (δ_C_143.3), C-33 (δ_C_ 154.6), and C-2 (δ_C_ 149.9) and from H-33 (δ_H_ 8.64, s) to C-3 (δ_C_ 157.3) and C-4 (δ_C_ 42.9) proved that the fragment of the 36,37,38-trihydroxybutyl unit was linked to C-32. Similar NOESY correlations and identical CD spectra of compounds **3** and **1** suggested their undifferentiated absolute configuration. Thus, the structure of **3** was elucidated as shown.

Compound **4** was obtained as a pale-yellow powder. The molecular formula of **4** was established to be C_38_H_54_N_2_O_9_ from its HRESIMS data. The NMR spectroscopic data (Table 1) of **4** displayed high similarity to those of **3**, except for an additional double bond at the side chain, which indicated that compound **4** was a dehyrdrogenated product of compound **3**, as proven by the HMBC experiment. In the HMBC spectrum, the cross peaks from H-23 (δ_H_ 7.35, 1H, d, *J* = 15.8 Hz) and H-24 (δ_H_ 7.41, 1H, d, *J* = 15.8 Hz) to C-22 (δ_C_ 204.8) and from H_3_-26 (δ_H_, 1.55) and H_3_-27 (δ_H_, 1.52) to C-24 (δ_C_ 150.2) supported this deduction. The identical CD spectra and similar optical rotation of compounds **3–4** indicated that they owed the same absolute configuration. Therefore, the structure of compound **4** was determined as depicted and given the trivial name siragrosvenin D.

**FIGURE 5:**
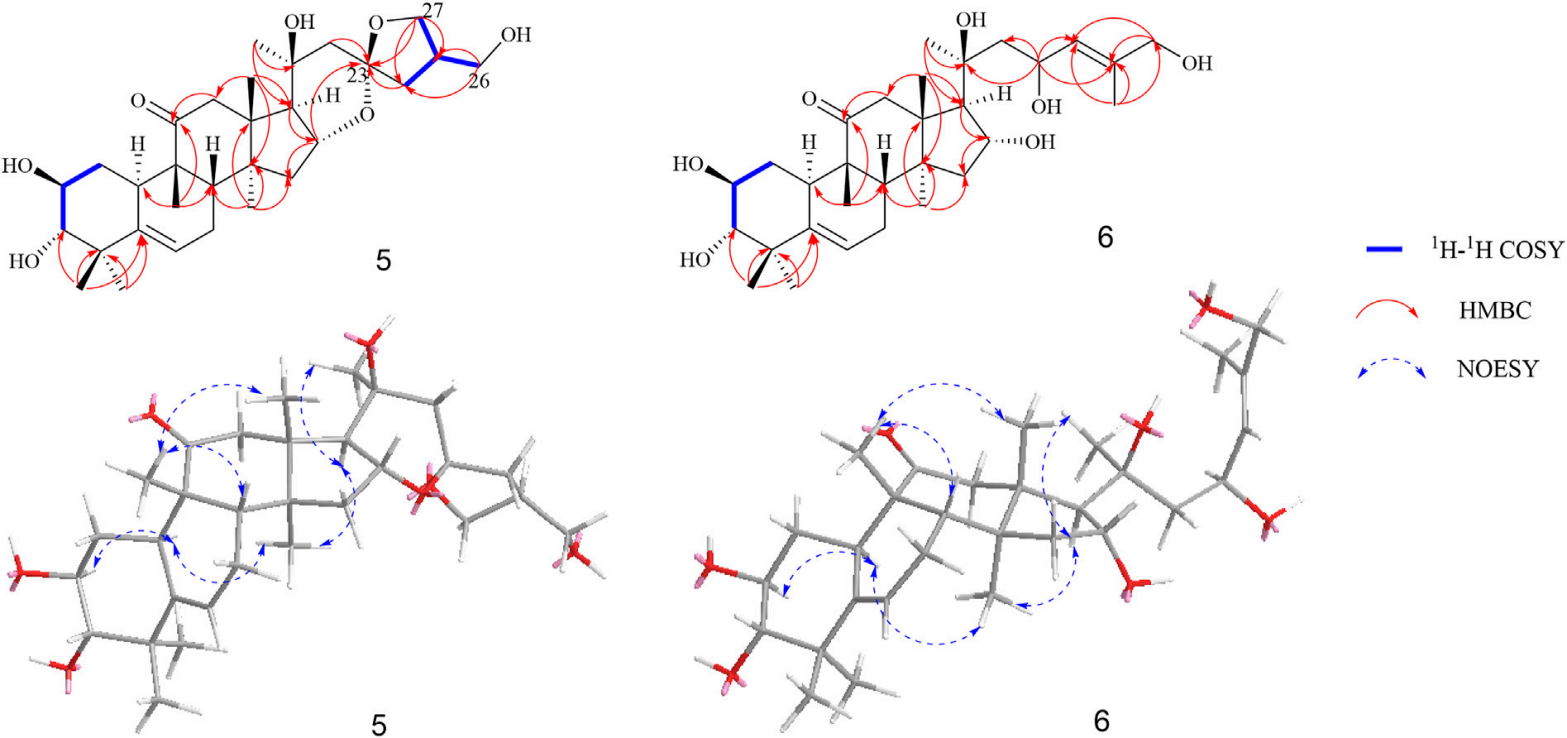
Key ^1^H-^1^H COZY, HMBC, and NOESY correlations of compounds **5** and **6**.

Compound **5** was isolated as a white amorphous powder, and it has a molecular formula of C_30_H_46_O_7_ as deduced on the basis of (M + Na)^+^ ion peak at *m/z* 541.3126 (calculated C_30_H_46_O_7_Na 541.3141) in HRESIMS, which indicated eight degrees of unsaturation. The ^1^H NMR data (Table 1) displayed six methyl groups (δ_H_ 1.22, 1.24, 1.31, 1.34, 1.36, and 1.47, each 3H, s), one olefinic methine [δ_H_ 5.69 (1H, m, H-6)], three oxygenated methines [δ_H_ 4.09 (1H, overlap, H-2), 3.43 (1H, m, H-3), and 5.00 (1H, m, H-16)], and two methylenes [δ_H_ 3.79 (1H, m, H-26a), 3.74 (1H, m, H-26b) and 4.22 (1H, m, H-27a), 4.09 (1H, overlap, H-27b)]. The ^13^C-APT NMR spectrum revealed 30 carbon signals attributed to one ketone carbon (δ_C_ 213.2), one pair of double bonds (δ_C_ 142.9 and 119.0), three oxygenated methines (δ_C_ 81.8, 71.3, and 70.8), two oxygenated quaternary carbons (δ_C_ 72.6 and 110.0), two oxygenated methylenes (δ_C_ 71.1 and 64.8), and six methyls (δ_C_ 20.2, 21.0, 21.7, 22.6, 25.8, and 29.0). Based on these data, compound **5** was classified as a cucurbitane triterpenoid (Clericuzio et al., 2004). Comparing the results of ^1^H and ^13^C NMR spectrum data of compound **5** with those of jinfushanencin F suggested their identical A/B/C/D rings (Li et al., 2016), except for the specific quaternary carbon signal at δ_C_ 110.0 in compound **5**. Considering the unsaturation of **5**, we inferred that there was an extra ring in the side chain. In the 2D NMR spectra, the fragment of C-24–C-25 (-C-27)-C-26 was established in the ^1^H-^1^H COZY experiment, while the HMBC correlations from H-27 (δ_H_ 4.22, 4.09) and H-24 (δ_H_ 2.33, 1.71) to δ_C_ 110.0 suggested the presence of a spiro ring as formed by aldol condensation at C-23 (Figure 5). Thus, the planar structure of **5** was elucidated as an uncommon triterpenoid structure, which showed the spiro ring in the side chain.

**FIGURE 6:**
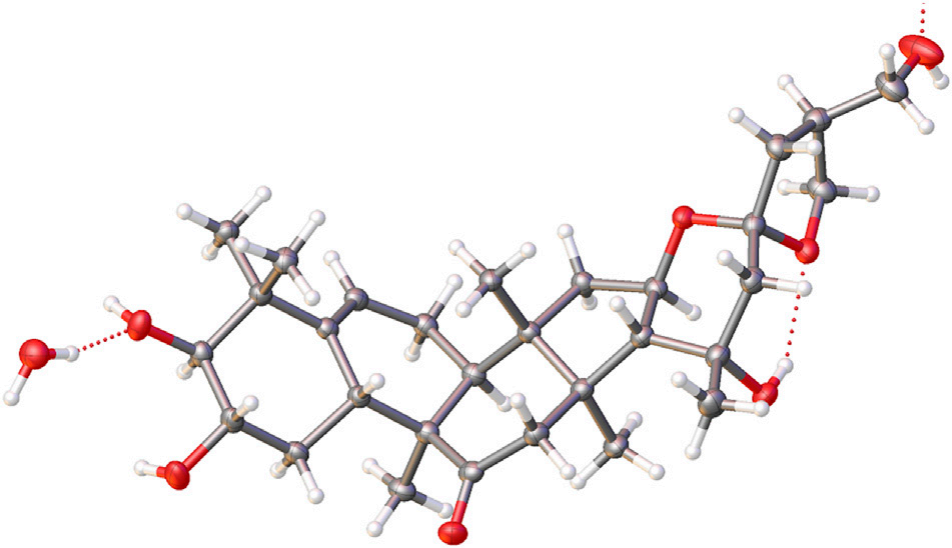
ORTEP diagram of **5**.

The relative configuration was elucidated based on the NOESY correlations. Intense correlation of H-2/H-10 and H-3/Me-19 indicated that 2-OH was in β-orientation and 3-OH was in α-orientation. In addition, the cross peaks of H-17/Me-30, H-10/Me-30, H-8/Me-18, and H-8/Me-19 suggested the β-orientation for H-10, Me-30, and H-17 and α-orientation for H-8, Me-18, and Me-19. Fortunately, a suitable crystal of **5** was obtained and the single-crystal X-ray diffraction analysis was performed using Cu–Kα radiation (Figure 6), which established the absolute configuration of **5** to be 2*S*, 3*S*, 8*S*, 9*R*, 10*R*, 13*R*, 14*S*, 16*R*, 17*R*, 20*R*, and 25*S*. Consequently, the structure of **5** was named as siragrosvenin E.

Compound **6** was obtained as a white amorphous powder and given the molecular formula of C_30_H_48_O_7_ based on HRESIMS. The ^1^H NMR spectrum of **6** also showed signals of a typical cucurbitacin triterpenoid with seven methyls at δ_H_ 1.27, 1.29, 1.32, 1.39, 1.49 (overlap), and 1.79 and two olefin protons at δ_H_ 5.15 (1H, m, H-23) and 6.06 (1H, m H-24). In addition, there were four oxygenated methines at δ_H_ 4.12 (1H, overlap, H-2), 3.45 (1H, d, *J* = 9 Hz, H-3), 4.92 (1H, m, H-16), and 5.15 (1H, m, H-23) and one methylene at δ_H_ 4.30 (2H, s, H-26). Its ^13^C NMR spectra exhibited thirty carbon signals ascribed to seven methyls, five methylenes, four alkene carbon atoms, six oxygenated carbons, and five quaternary carbons (a carbonyl and an oxygenated carbon). The spectroscopic data displayed a resemblance to those of the known compound jinfushanencin F (Li et al., 2016), except for the more 18 amu than jinfushanencin F, which indicated that compound **6** could be a hydrolyzate of jinfushanencin F in the C-16–O-23 moiety. The key HMBC correlations from H-23 (δ_H_ 5.15) to C-20 (δ_C_ 71.8), C-22 (δ_C_ 50.0), C-24 (δ_C_ 126.2), and C-25 (δ_C_ 138.5) suggested that one hydroxy group was connected to C-23, and HMBC correlations from H-16 (δ_H_ 4.92) to C-13 (δ_C_ 49.1), C-14 (δ_C_ 48.9), C-17 (δ_C_ 56.3), and C-20 (δ_C_ 71.8) showed that another hydroxy group was attached to C-16 (Figure 5). The relative configurations of **6** were similar to those of compound **5** according to their similar NOESY correlations and NMR data. The ECD spectrum of **6** also showed same cotton effects to those of **5,** suggesting identical absolute configuration. However, with rotational freedom in the side chain, it was not possible to definitively assign the C-23 configuration for either epimer. Thus, the structure of **6** was elucidated as depicted, named siragrosvenin F.

In addition, eight known compounds were isolated from the roots of *S. grosvenorii*. Their structures were identified as 23,24-dihydrocucurbitacin F (**7**) (Guerrero-Analco et al., 2007), cucurbitacin IIa (**8**) (Zeng et al., 2021), cucurbitacin Q1 (**9**) (Add El-Fattah, 1994), jinfushanencin F (**10**) (Li et al., 2016), siraitic acid A (**11**) (Si et al., 1999), siraitic acid B (**12**) (Si et al., 1999), cucurbitacin E (**13**) (Attard et al., 2005), and 23,24-dihydrocucurbitacin E (**14**) (Tang et al., 2015).

### Proposed Biosynthetic Pathways for the Formation of Compounds **1–4**

**FIGURE 7:**
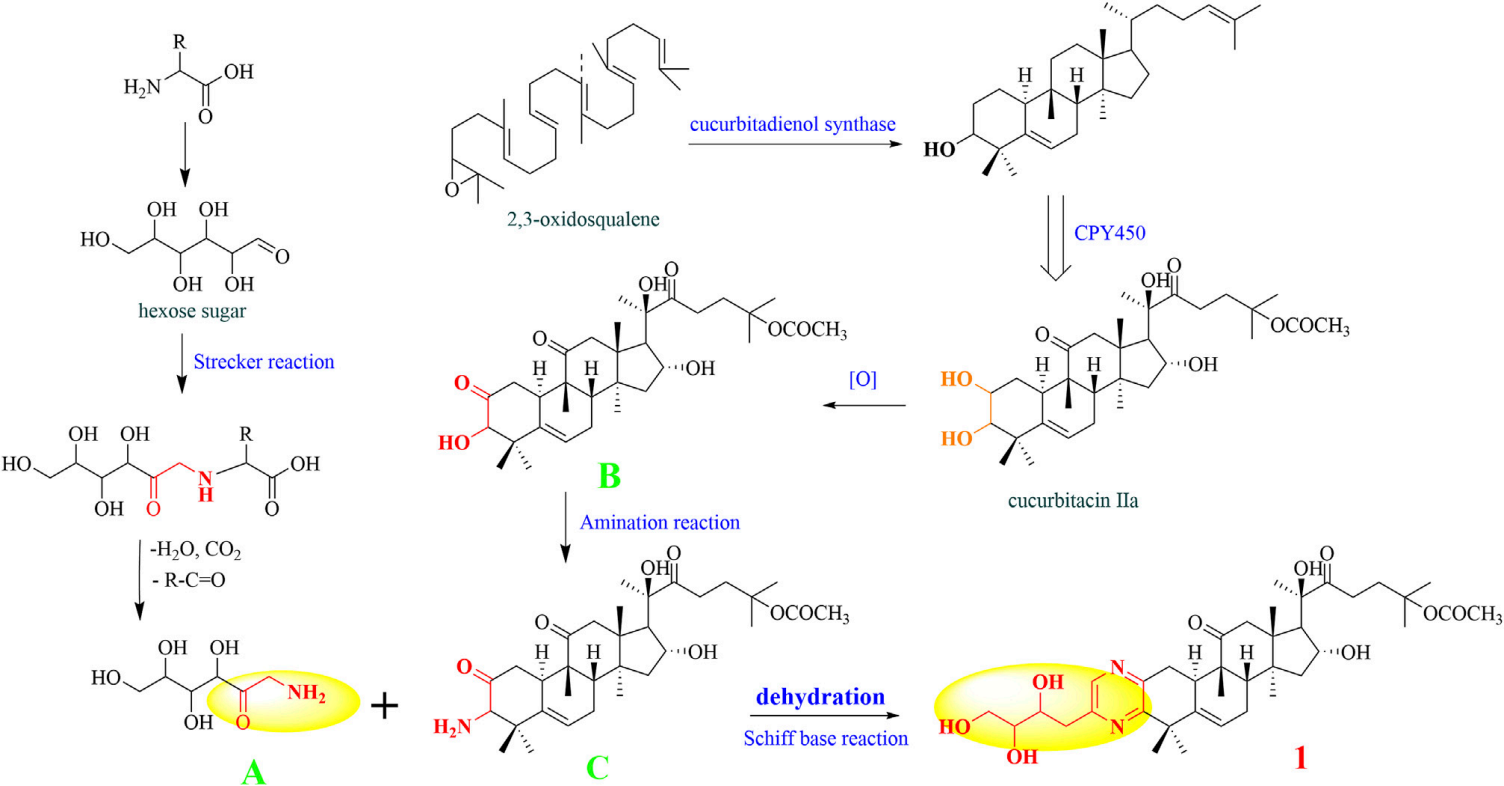
Putative biosynthetic pathway of compound **1**.

As far as we know, none of the identified cucurbitane-type pyrazine triterpenoid alkaloids has been reported as natural products so far in the literature. Consequently, we tried to deduce the potential biosynthetic pathway of compound **1** (Figure 7). First, the free amino acids in plants could react with the carbonyl group of hexose sugar. After the Strecker reaction and dehydration reaction, we obtained the amide-type conjugation (A) (Fujii and Kobatake, 1972; Wakamatsu et al., 2019). Simultaneously, 2, 3-oxysqualene was protonated, cyclized, rearranged, and deprotonated under the catalysis of various 2,3-oxidosqualene cyclases (OSCs) to obtain triterpenoid precursors, such as cucurbitenol. Then, cucurbitacin IIa was obtained under the catalysis of various cytochrome oxygenases P450 (CPY450), and the oxidation of its C-2 hydroxyl group will produce the derivative iso-23,24-dihydrocucurbitacin B (B), which could trigger the next chemical reaction. Subsequently, the precursor (B) was further aminated to form the structure C. The formation process of the pyrazines can be explained by the occurrence of Schiff’s base reaction by the degradation of the carbonyl group and amide group (Ganesan, 1996), which was easier to take place in the plants to yield compound **1**. Finally, structures **2–4** could be generated by a similar mechanism as that described for structure **1**.

### Cytotoxicity Evaluation of all Isolates

Further studies were also performed using the MTT viability assay to evaluate the cytotoxicity of compounds **1−14** against MGC-803 (human gastric cancer cells), MCF-7 (human breast cancer cells), and CNE-1 (human nasopharyngeal carcinoma cells), and taxol (diterpene alkaloid) was used as a positive control. As shown in Table 4, compounds **4, 8, 9, 13**, and **14** exhibited obvious *in vitro* cytotoxicity, with IC_50_ values ranging from 1.44 to 9.99 μM. Cucurbitacin E, with the lowest IC_50_ values among those compounds, has been investigated extensively for its cytotoxicity toward several cancer cell lines through various underlying molecular mechanisms (Sun et al., 2010; Zhang et al., 2012; Attard and Martinoli, 2015). Through the analysis of the structure–activity relationship, α–β unsaturated ketone as functional groups can significantly enhance the cytotoxicity of cucurbitane-type compounds (Lin et al., 2015). In addition, compound **4** also showed the potential of cytotoxicity with an IC_50_ of 8.04 μM.

**TABLE 4:** Cytotoxicity of compounds **1**–**14** (IC_50_, μM).

	MGC-803	MCF-7	CNE-1
**1**	68.54 ± 0.33	>100	>100
**2**	34.14 ± 0.88	14.79 ± 1.22	13.75 ± 1.87
**3**	>100	>100	>100
**4**	12.30 ± 0.61	8.04 ± 0.63	8.86 ± 0.22
**5**	>100	>100	>100
**6**	58.40 ± 5.08	34.80 ± 3.50	31.65 ± 2.25
**7**	48.24 ± 4.42	>100	35.58 ± 1.81
**8**	3.96 ± 1.78	93.08 ± 3.62	>100
**9**	12.89 ± 4.98	7.49 ± 0.07	2.14 ± 0.39
**10**	>100	>100	>100
**11**	46.54 ± 1.94	>100	55.32 ± 3.32
**12**	>100	>100	>100
**13**	2.48 ± 0.48	3.24 ± 0.35	1.44 ± 0.31
**14**	9.99 ± 2.79	46.93 ± 2.55	5.99 ± 0.50
Taxol	0.56 ± 0.09	1.49 ± 0.22	2.72 ± 0.19

### Cytotoxicity Against MCF-7 Cells of Siragrosvenin D (4)

**FIGURE 8:**
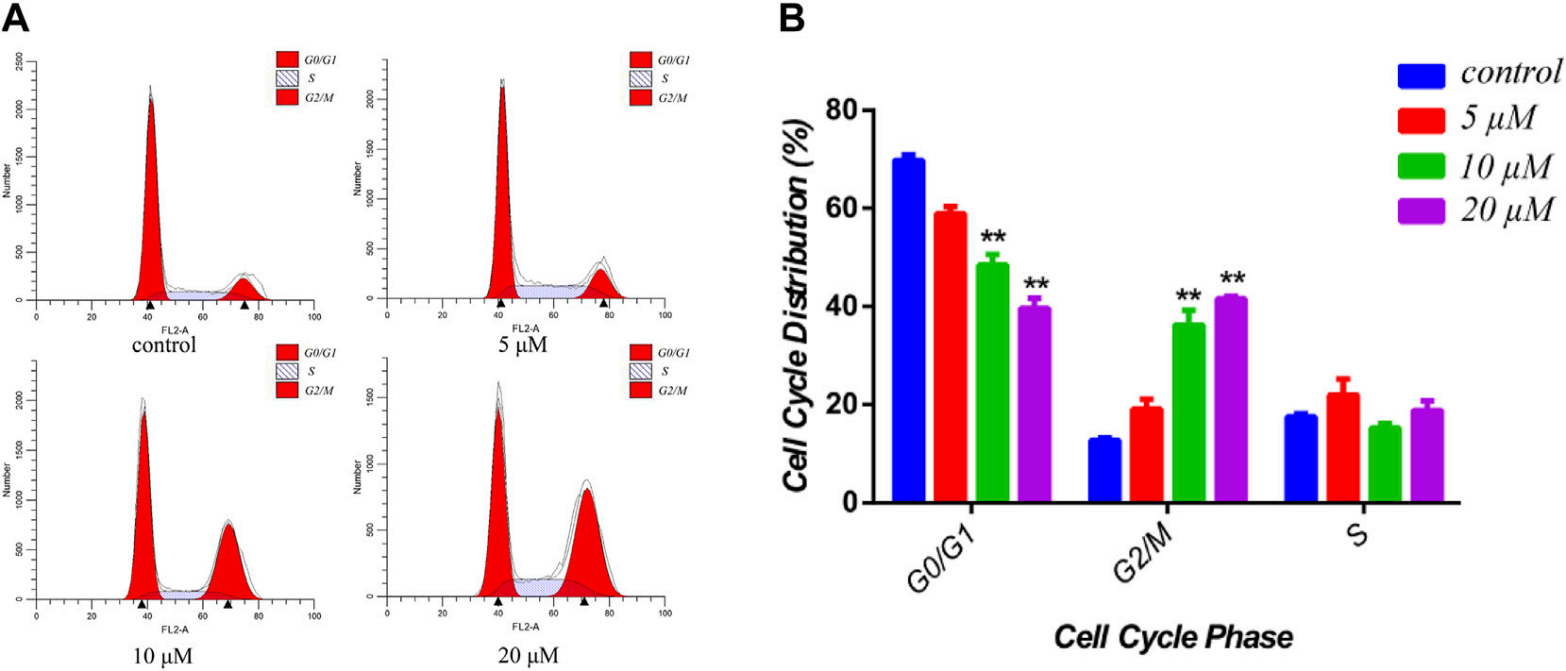
Effects of siragrosvenin D on cell cycle distribution in MCF-7 cells. **(A)** MCF-7 cells were treated by siragrosvenin D (0, 5, 10, and 20 μM) for 24 h and then analyzed by flow cytometry for cell cycle distribution. **(B)** Data were presented as the mean ± SD of three independent experiments. **p < 0.01 vs. control was considered statistically significant.

Cucurbitacin E, as the most anticancer potential natural products of the isolated compounds, has been investigated extensively for its cytotoxic activities toward several cancer cell lines through the various underlying molecular mechanisms (Huang et al., 2012; Attard and Martinoli, 2015). Therefore, to investigate the cytotoxicity of the siragrosvenin D, the cells were exposed to different concentrations of this compound. In cell cycle analysis, siragrosvenin D significantly arrested the growth of cells at the G2/M phase, increasing from 12.70% of cells treated with the negative control to 13.44, 36.25, and 41.56% of cells treated with 5, 10, and 20 μM of siragrosvenin D, respectively (Figure 8). These results implied that siragrosvenin D inhibited proliferation of MCF-7 cells *via* the induction of G2/M phase arrest.

**FIGURE 9:**
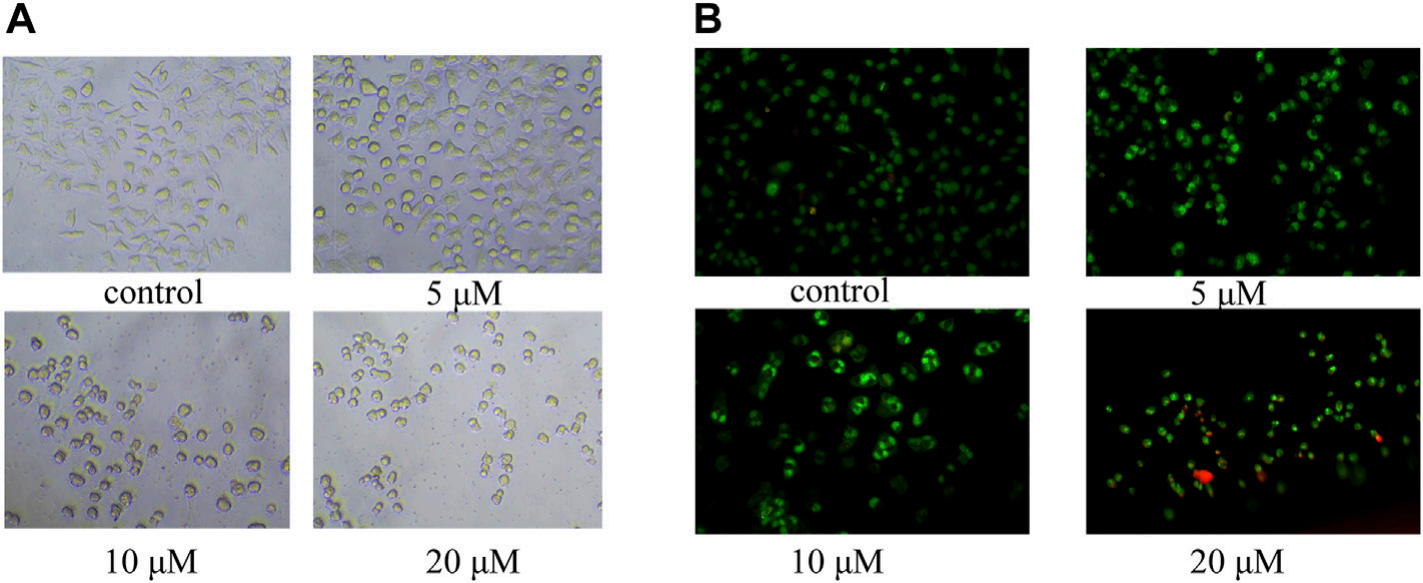
Apoptosis induced by siragrosvenin D in MCF-7 cells detected by the fluorescence microscope. **(A)** Morphology of the cells after being treated with siragrosvenin D (0, 5, 10, and 20 μM) for 24 h. **(B)** Percentage of apoptotic cells by AO/EB staining after being treated with siragrosvenin D (0, 5, 10, and 20 μM) for 24 h.

**FIGURE 10:**
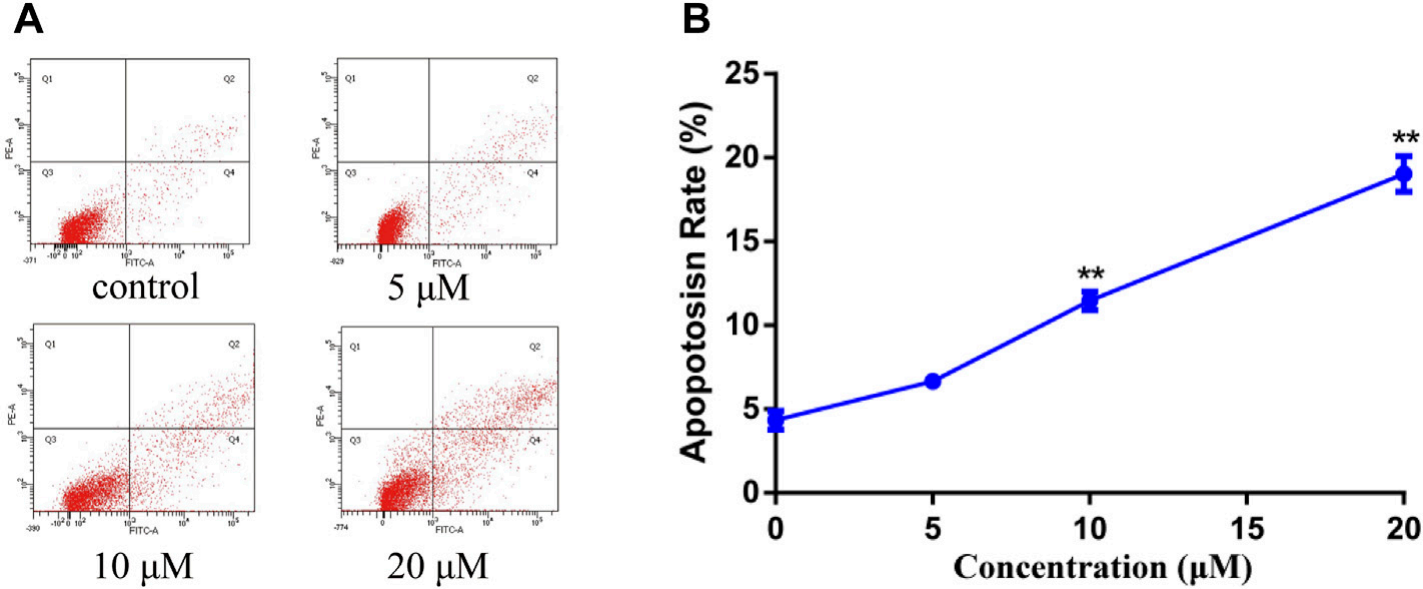
Apoptosis induced by siragrosvenin D in MCF-7 cells detected by the annexin V-FITC/PI staining test. MCF-7 cells were treated with siragrosvenin D (0, 5, 10, and 20 μM) for 24 h. **(A)** Apoptotic rates were determined by annexin V-FITC/PI staining. Apoptosis rate was reported as the percentage of apoptotic cells (AV+/PI− and AV+/PI+) among total cells. **(B)** Data were presented as the mean ± SD (*n* = 3). **p < 0.01.

In order to study the mechanism of the promoting effect of siragrosvenin D on cell apoptosis, first, we observed the morphology of the cells after being treated with siragrosvenin D (0, 5, 10, and 20 μM). Comparing to negative control, with the increase of the concentration of siragrosvenin D, the number of cells started to decrease, accompanied with contraction and exfoliation (Figure 9). The results of AO/EB staining also demonstrated that siragrosvenin D significantly increased the percentage of apoptotic cells in the treated cells, which showed red fluorescence. Moreover, cell apoptosis was analyzed by flow cytometry. As shown in Figure 10, siragrosvenin D induced apoptosis of MCF-7 cells evidently. The percentage of apoptotic and necrotic cells in the treated cells was increased in a dose-dependent manner compared with that in the control group.

**FIGURE 11:**
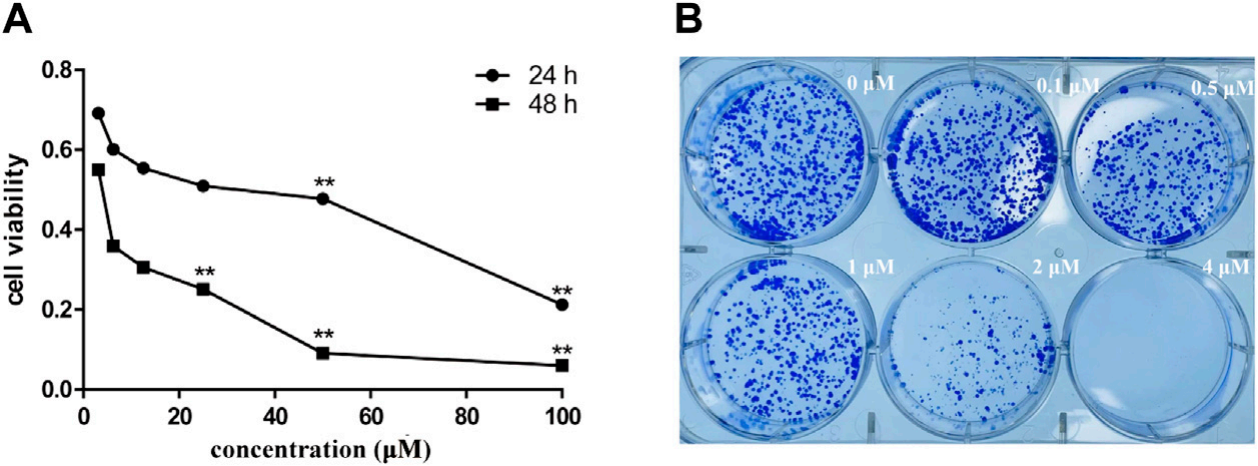
Cytotoxicity of siragrosvenin D. **(A)** MTT assay was performed to assess cell viability in MCF-7 cells treated with different concentrations of siragrosvenin D for 24 and 48 h. **(B)** Effect of siragrosvenin D on colony formation. All data are presented as mean ± SD. **p < 0.01.

To assess the effect of siragrosvenin D on the proliferation of MCF-7 cells, we treated the cells with different concentrations of siragrosvenin D (0, 5, 10, and 20 μM) and evaluated its cell viability by the MTT assay for 24 and 48 h. It was observed that the growth of MCF-7 cells was suppressed in a dose- and time-dependent manner (Figure 11A). In addition, colony formation assay showed that after 14 days of incubation with siragrosvenin D, the number of colonies in the treated groups was significantly less than that in the control group (Figure 11B).

## Conclusion

In summary, four novel cucurbitane-type triterpenoid pyrazine alkaloids, siragrosvenins A–D (**1–4**), along with two new cucurbitacins, siragrosvenins E–F (**5–6**), were isolated from the roots of *S. grosvenorii*. Among them, compounds **1–4** contained a novel cucurbitane-type triterpenoid skeleton with an additional pyrazine unit *via* a carbon–nitrogen linkage in the structure. Compound **5** showed an unexpected triterpenoid structure with a 6/6/6/5/6/5-fused polycyclic ring system, through aldol condensation. Although the pyrazine moiety is ever reported in plants (Li et al., 2006), siragrosvenins A–D (**1−4**) are the first examples of cucurbitane-type triterpenoid pyrazine alkaloids isolated from the herbs and may provide new chemotypes for the development of novel promising anticancer agents. Siragrosvenin B and D showed more significant cytotoxicity against the tested cell lines, which further confirmed that the presence of the α,β-unsaturated ketone moiety could improve the antitumor activity. A widely accepted mechanism for these compounds was the occurrence of the Michael addition between the α,β-unsaturated ketone fraction and the soft nucleophiles, such as mercaptan and protein sulfhydryl groups, resulting in the inactivation of the SH enzyme or SH coenzyme (Wijeratne et al., 2012).

Furthermore, we also conducted a preliminary investigation on siragrosvenin D, and the results implied that siragrosvenin D inhibited proliferation of MCF-7 cells and reduced their viability *via* the induction of G2/M phase arrest and significantly induced apoptosis in MCF-7 cells.

## Data Availability

The original contributions presented in the study are publicly available. These data can be found here: https://www.ccdc.cam.ac.uk/structures/, 2151134.
