# Supplementary material for: Novel Triterpenoid Alkaloids With Their Potential Cytotoxic Activity From the Roots of Siraitia grosvenorii
Source: Front Chem. 2022 Apr 29;10:885487. doi: 10.3389/fchem.2022.885487 (PMC9099095; doi:10.3389/fchem.2022.885487)
Supplement: Supplementary file 1 [file DataSheet1.docx]

***Supplementary Material***

**Contents**

**MS, IR, UV, ECD and NMR Spectra of Compound 1**

Figure S1. HR-ESI-MS spectrum of **1**

Figure S2. IR spectrum of **1**

Figure S3. UV spectrum in MeOH of **1**

Figure S4. ECD spectrum in MeOH of **1**

Figure S5. ^1^H-NMR spectrum (Pyrdine-D_5_, 600 MHz) of **1**

Figure S6. ^13^C-APT NMR spectrum (Pyrdine-D_5_, 125 MHz) of **1**

Figure S7. ^1^H-^1^H COSY spectrum (Pyrdine-D_5_) of **1**

Figure S8. HSQC spectrum (Pyrdine-D_5_) of **1**

Figure S9. HMBC spectrum (Pyrdine-D_5_) of **1**

Figure S10. NOESY spectrum (Pyrdine-D_5_) of **1**

Figure S11. ^1^H-NMR spectrum (CD_3_OD, 600 MHz) of **1**

**MS, IR, UV, ECD and NMR Spectra of Compound 2**

Figure S12. HR-ESI-MS spectrum of **2**

Figure S13. IR spectrum of **2**

Figure S14. UV spectrum in MeOH of **2**

Figure S15. ECD spectrum in MeOH of **2**

Figure S16. ^1^H-NMR spectrum (Pyrdine-D_5_, 600 MHz) of **2**

Figure S17. ^13^C-APT NMR spectrum (Pyrdine-D_5_, 125 MHz) of **2**

Figure S18. ^1^H-^1^H COSY spectrum (Pyrdine-D_5_) of **2**

Figure S19. HSQC spectrum (Pyrdine-D_5_) of **2**

Figure S20. HMBC spectrum (Pyrdine-D_5_) of **2**

Figure S21. NOESY spectrum (Pyrdine-D_5_) of **2**

**MS, IR, UV, ECD and NMR Spectra of Compound 3**

Figure S22. HR-ESI-MS spectrum of **3**

Figure S23. IR spectrum of **3**

Figure S24. UV spectrum in MeOH of **3**

Figure S25. ECD spectrum in MeOH of **3**

Figure S26. ^1^H-NMR spectrum (Pyrdine-D_5_, 600 MHz) of **3**

Figure S27. ^13^C-APT NMR spectrum (Pyrdine-D_5_, 125 MHz) of **3**

Figure S28. ^1^H-^1^H COSY spectrum (Pyrdine-D_5_) of **3**

Figure S29. HSQC spectrum (Pyrdine-D_5_) of **3**

Figure S30. HMBC spectrum (Pyrdine-D_5_) of **3**

Figure S31. NOESY spectrum (Pyrdine-D_5_) of **3**

Figure S32. ^1^H-NMR spectrum (CD_3_OD, 600 MHz) of **3**

**MS, IR, UV, ECD and NMR Spectra of Compound 4**

Figure S33. HR-ESI-MS spectrum of **4**

Figure S34. IR spectrum of **4**

Figure S35. UV spectrum in MeOH of **4**

Figure S36. ECD spectrum in MeOH of **4**

Figure S37. ^1^H-NMR spectrum (Pyrdine-D_5_, 600 MHz) of **4**

Figure S38. ^13^C-APT NMR spectrum (Pyrdine-D_5_-D5, 125 MHz) of **4**

Figure S39. ^1^H-^1^H COSY spectrum (Pyrdine-D_5_) of **4**

Figure S40. HSQC spectrum (Pyrdine-D_5_) of **4**

Figure S41. HMBC spectrum (Pyrdine-D_5_) of **4**

Figure S42. NOESY spectrum (Pyrdine-D_5_) of **4**

**MS, IR, UV, ECD and NMR Spectra of Compound 5**

Figure S43. HR-ESI-MS spectrum of **5**

Figure S44. IR spectrum of **5**

Figure S45. UV spectrum in MeOH of **5**

Figure S46. ECD spectrum in MeOH of **5**

Figure S47. ^1^H-NMR spectrum (Pyrdine-D_5_, 600 MHz) of **5**

Figure S48. ^13^C-APT NMR spectrum (Pyrdine-D_5_, 125 MHz) of **5**

Figure S49. ^1^H-^1^H COSY spectrum (Pyrdine-D_5_) of **5**

Figure S50. HSQC spectrum (Pyrdine-D_5_) of **5**

Figure S51. HMBC spectrum (Pyrdine-D_5_) of **5**

Figure S52. NOESY spectrum (Pyrdine-D_5_) of **5**

**MS, IR, UV, ECD and NMR Spectra of Compound 6**

Figure S53. HR-ESI-MS spectrum of **6**

Figure S54. IR spectrum of **6**

Figure S55. UV spectrum in MeOH of **6**

Figure S56. ECD spectrum in MeOH of **6**

Figure S57. ^1^H-NMR spectrum (Pyrdine-D_5_, 600 MHz) of **6**

Figure S58. ^13^C-APT NMR spectrum (Pyrdine-D_5_, 125 MHz) of **6**

Figure S59. ^1^H-^1^H COSY spectrum (Pyrdine-D_5_) of **6**

Figure S60. HSQC spectrum (Pyrdine-D_5_) of **6**

Figure S61. HMBC spectrum (Pyrdine-D_5_) of **6**

Figure S62. NOESY spectrum (Pyrdine-D_5_) of **6**

**S-1 Calculation of ^13^C NMR Spectra for 1**

**MS, IR, UV, ECD and NMR Spectra of Compound 1**

Figure S1. HR-ESI-MS spectrum of **1**


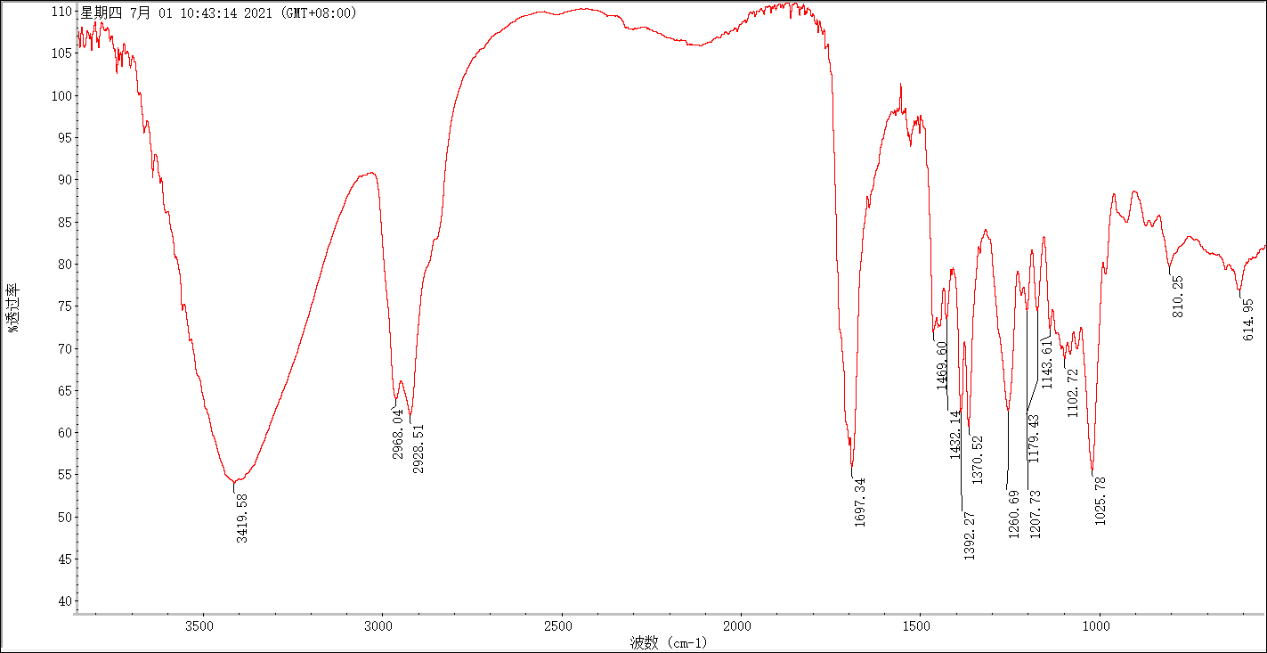


Figure S2. IR spectrum of **1**

Figure S3. UV spectrum in MeOH of **1**


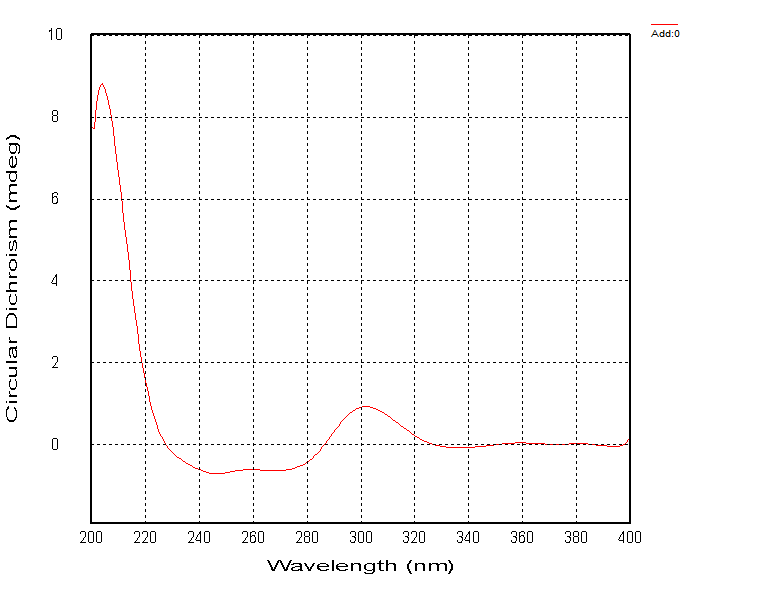


Figure S4. ECD spectrum in MeOH of **1**


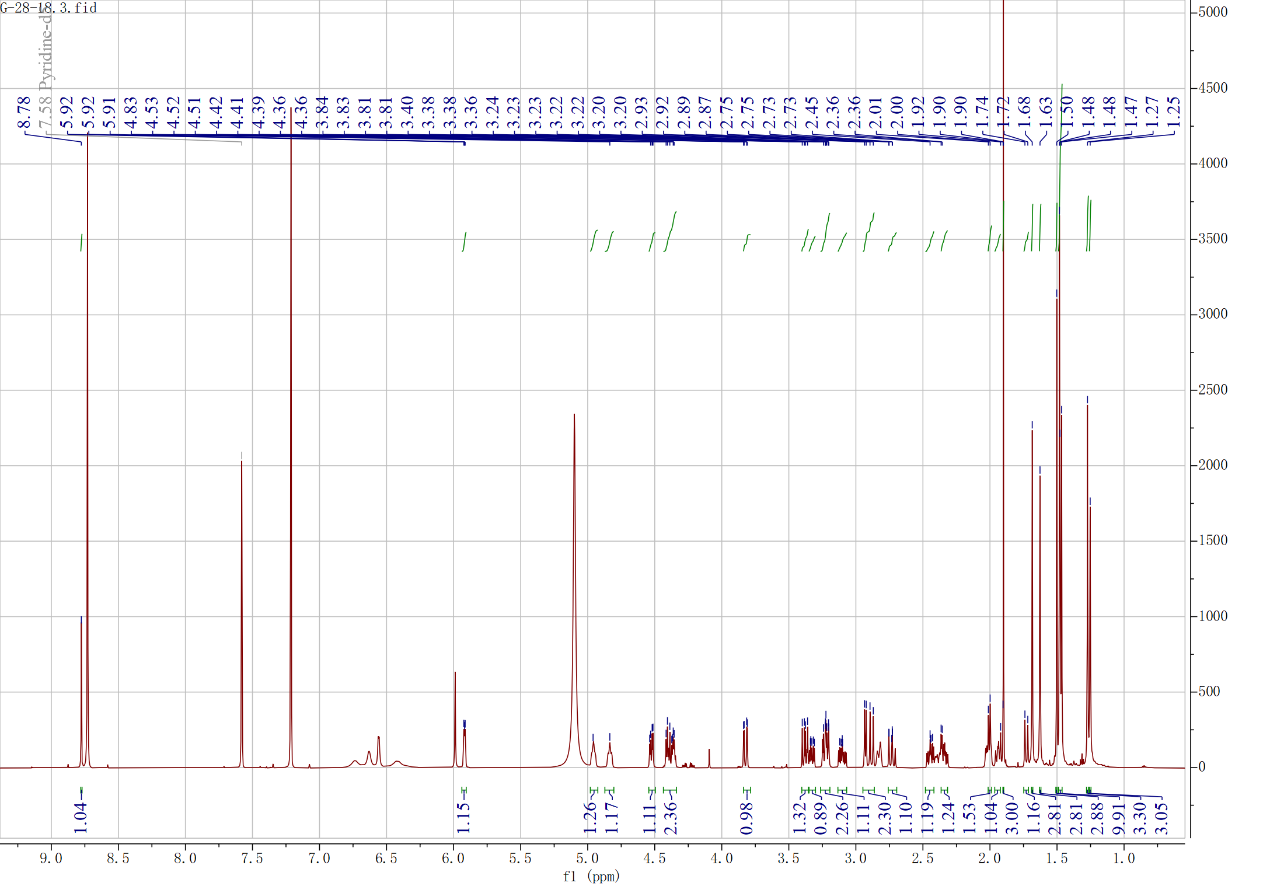


Figure S5. ^1^H-NMR spectrum (Pyrdine-D_5_, 600 MHz) of **1**


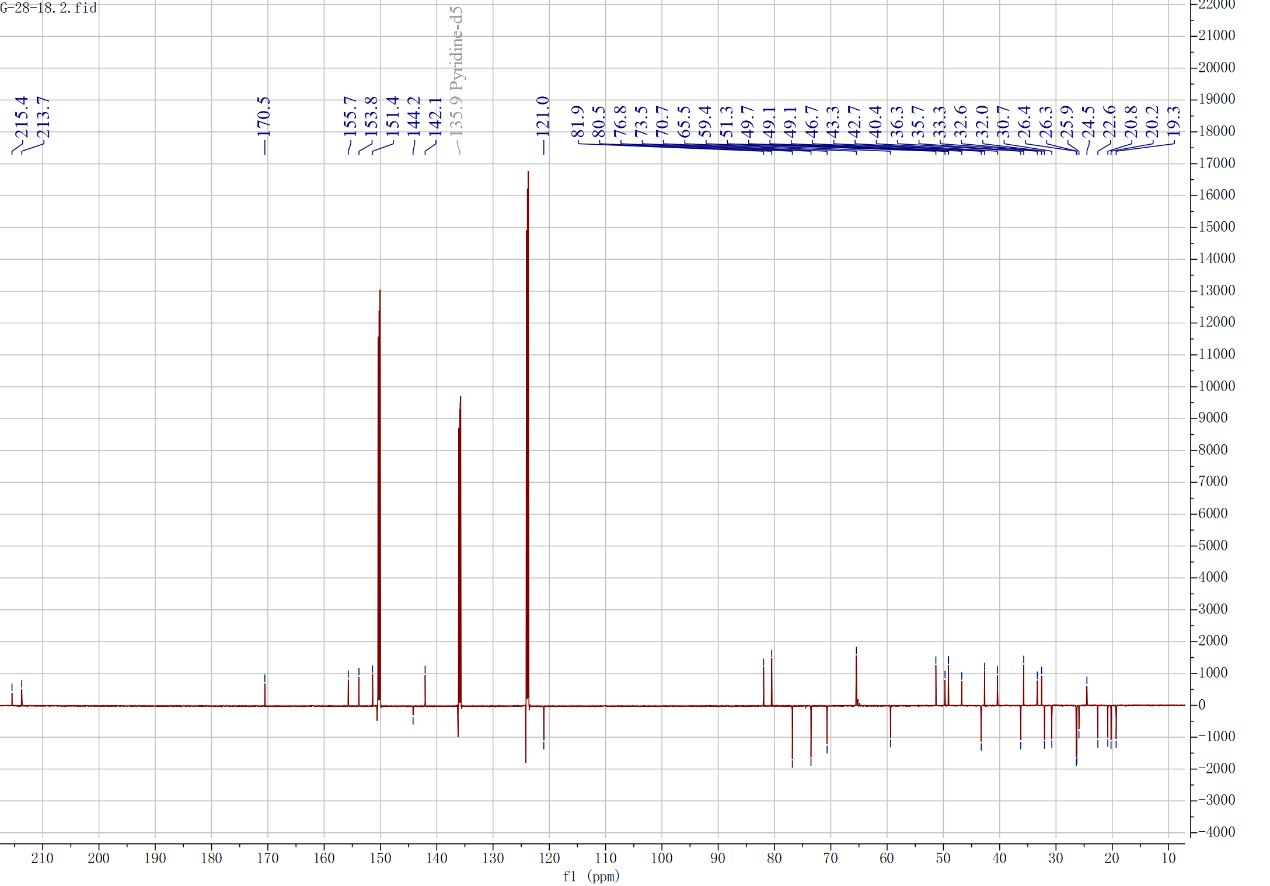


Figure S6. ^13^C-APT NMR spectrum (Pyrdine-D_5_, 125 MHz) of **1**


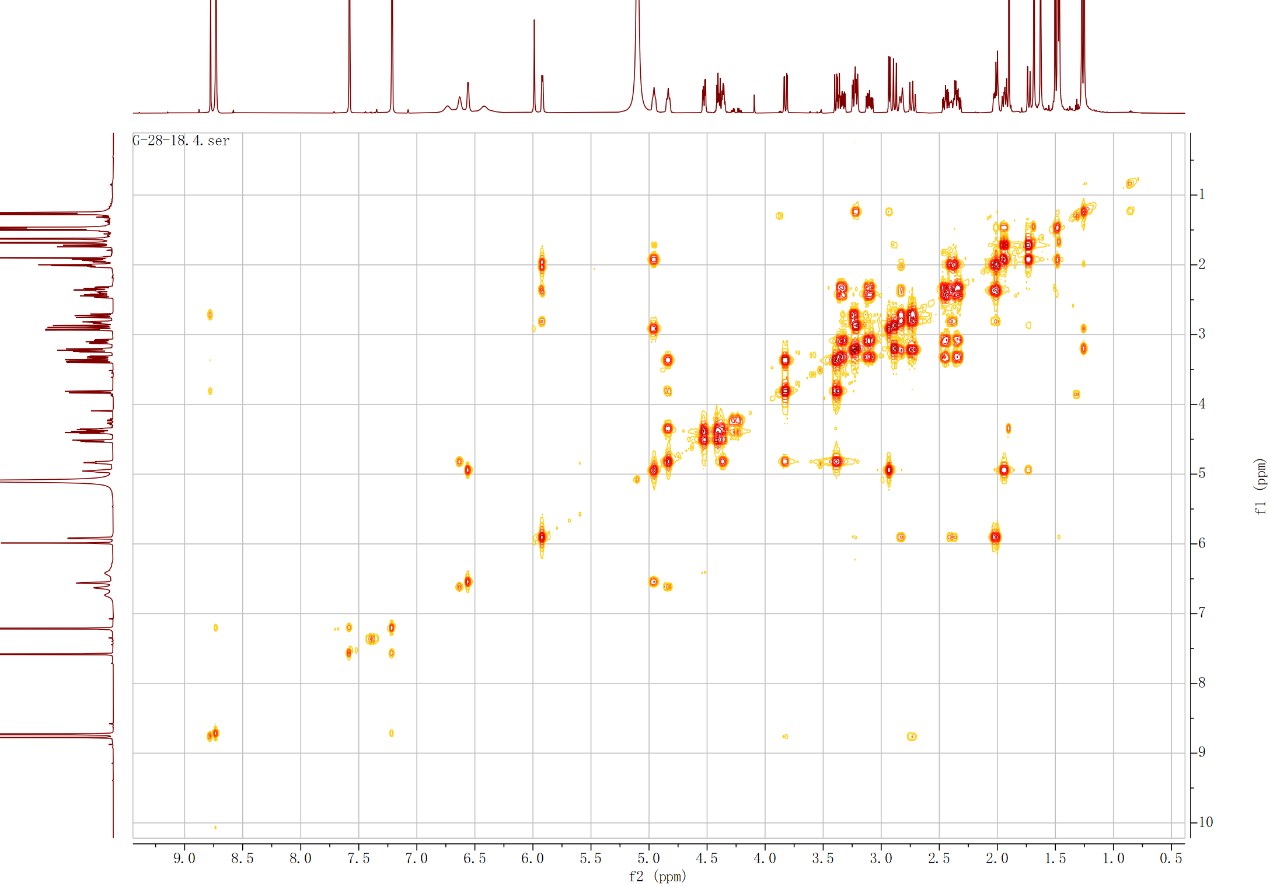


Figure S7. ^1^H-^1^H COSY spectrum (Pyrdine-D_5_) of **1**


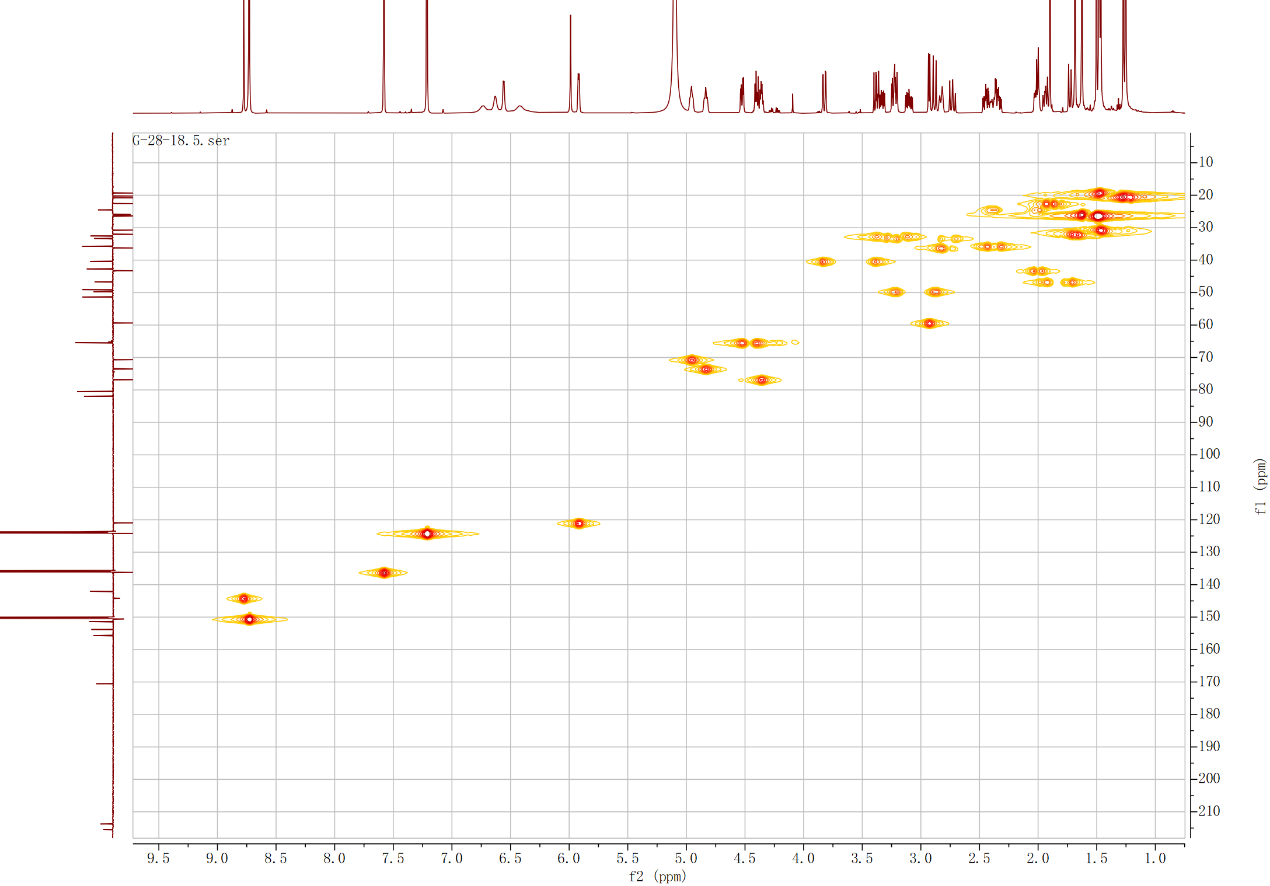


Figure S8. HSQC spectrum (Pyrdine-D_5_) of **1**


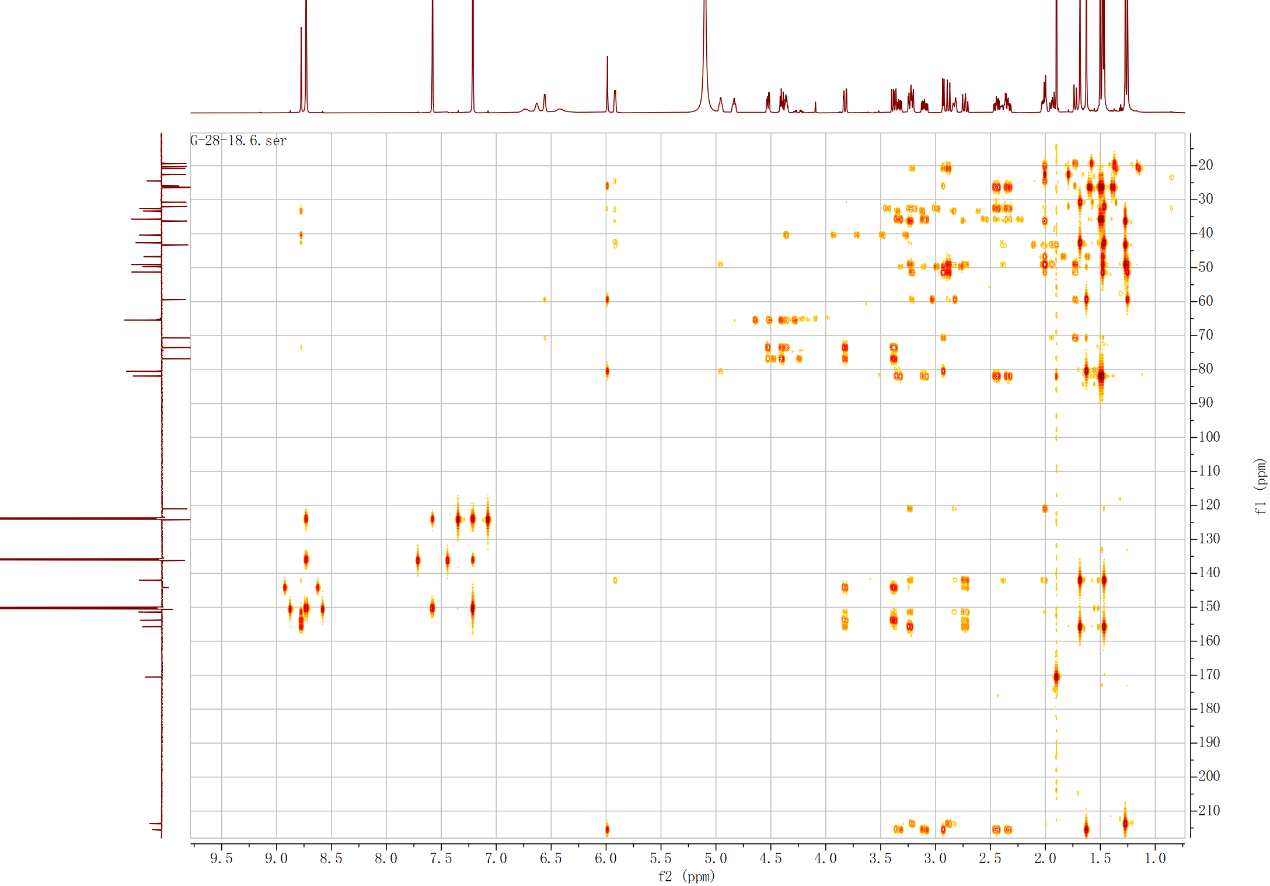


Figure S9. HMBC spectrum (Pyrdine-D_5_) of **1**


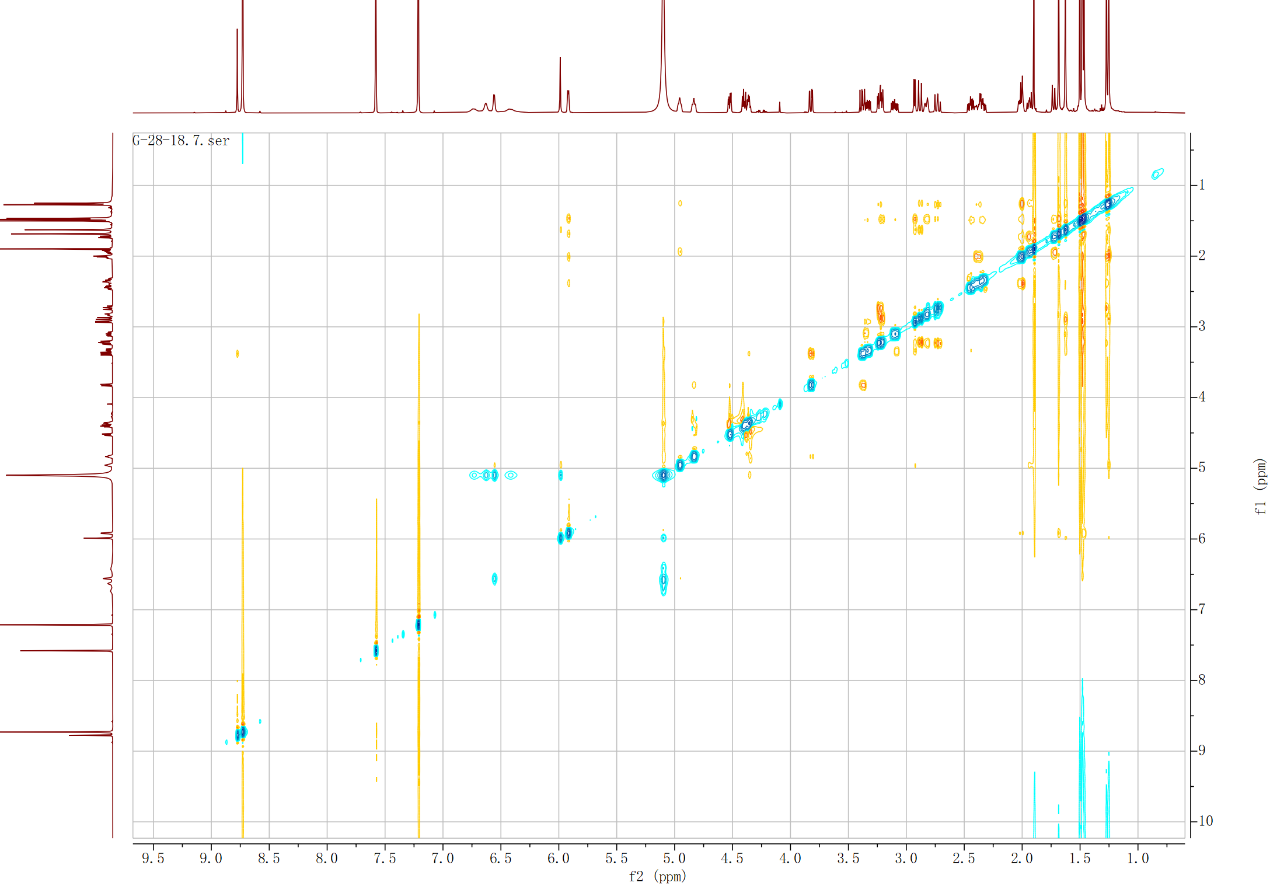


Figure S10. NOESY spectrum (Pyrdine-D_5_) of **1**


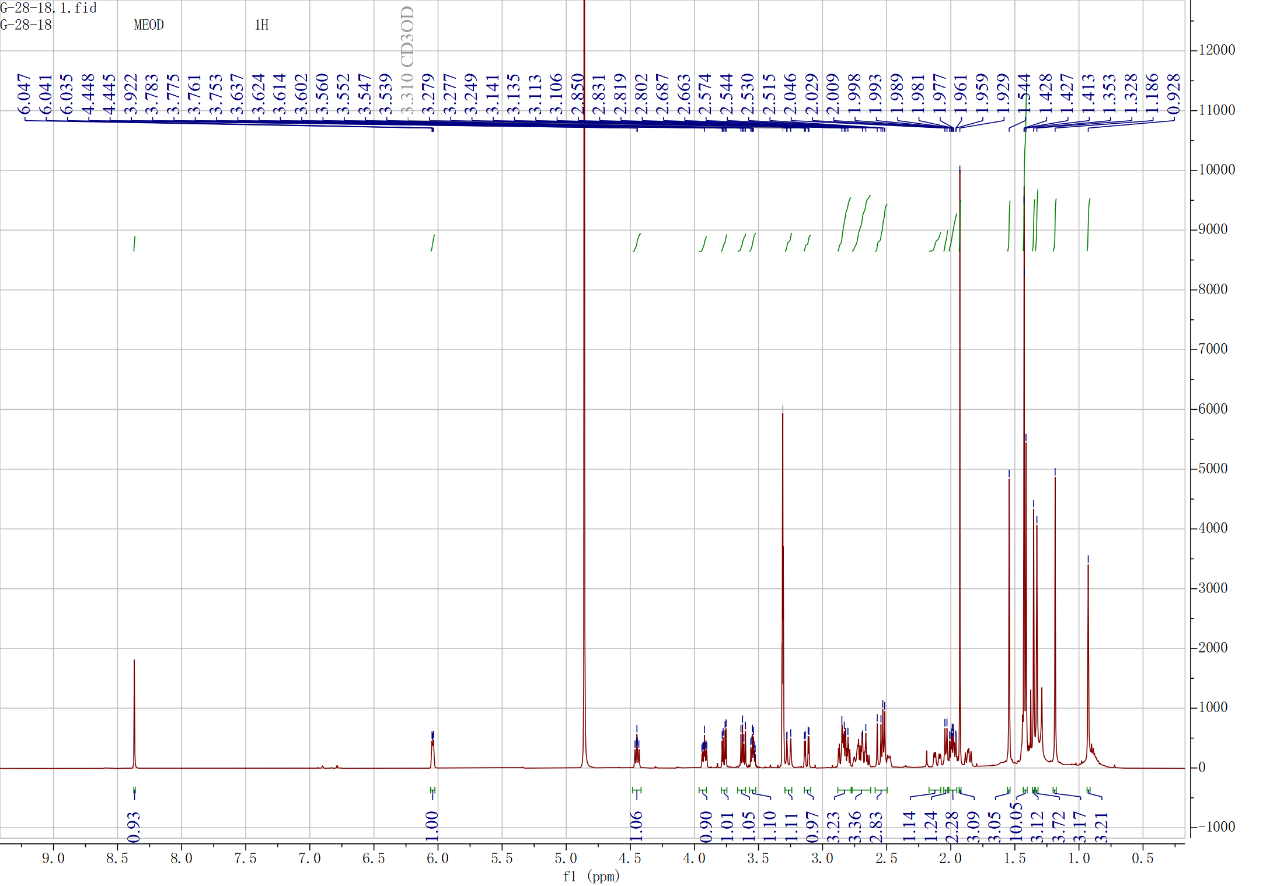


Figure S11. ^1^H-NMR spectrum (CD_3_OD, 600 MHz) of **1**

**MS, IR, UV, ECD and NMR Spectra of Compound 2**

Figure S12. HR-ESI-MS spectrum of **2**


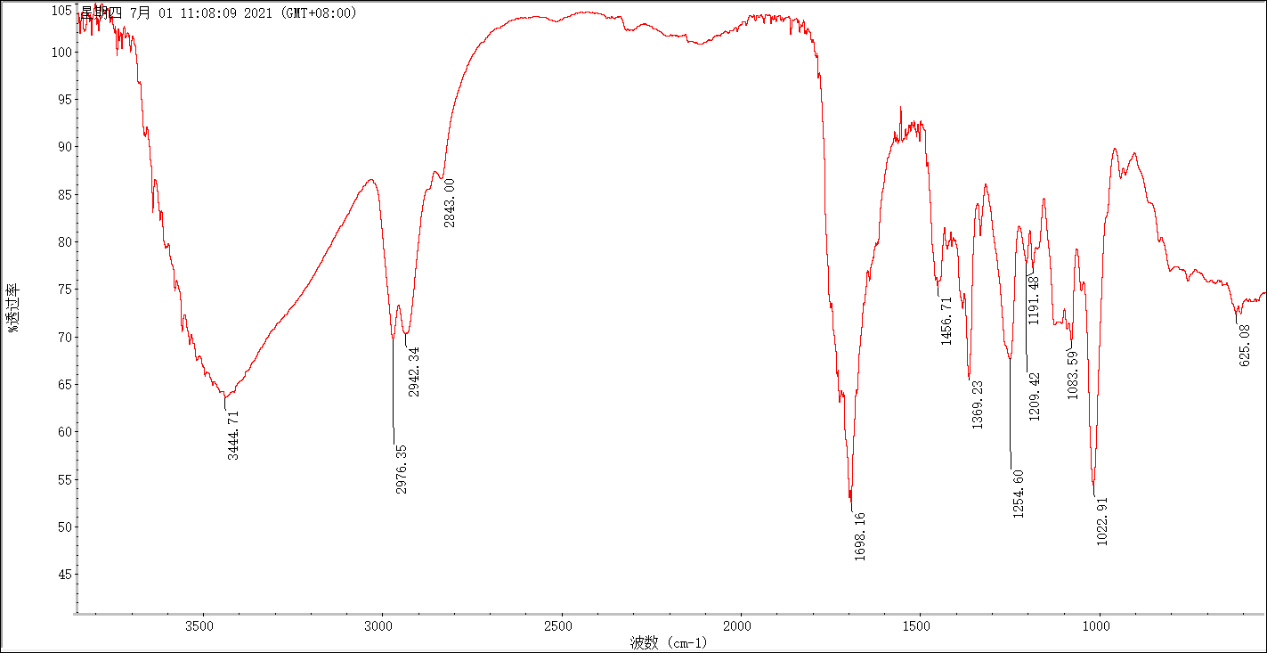


Figure S13. IR spectrum of **2**

Figure S14. UV spectrum in MeOH of **2**


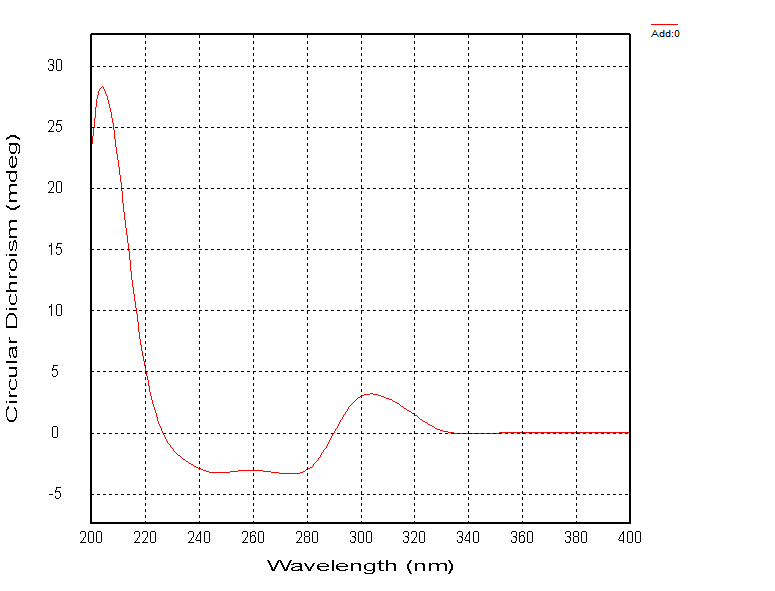


Figure S15. ECD spectrum in MeOH of **2**


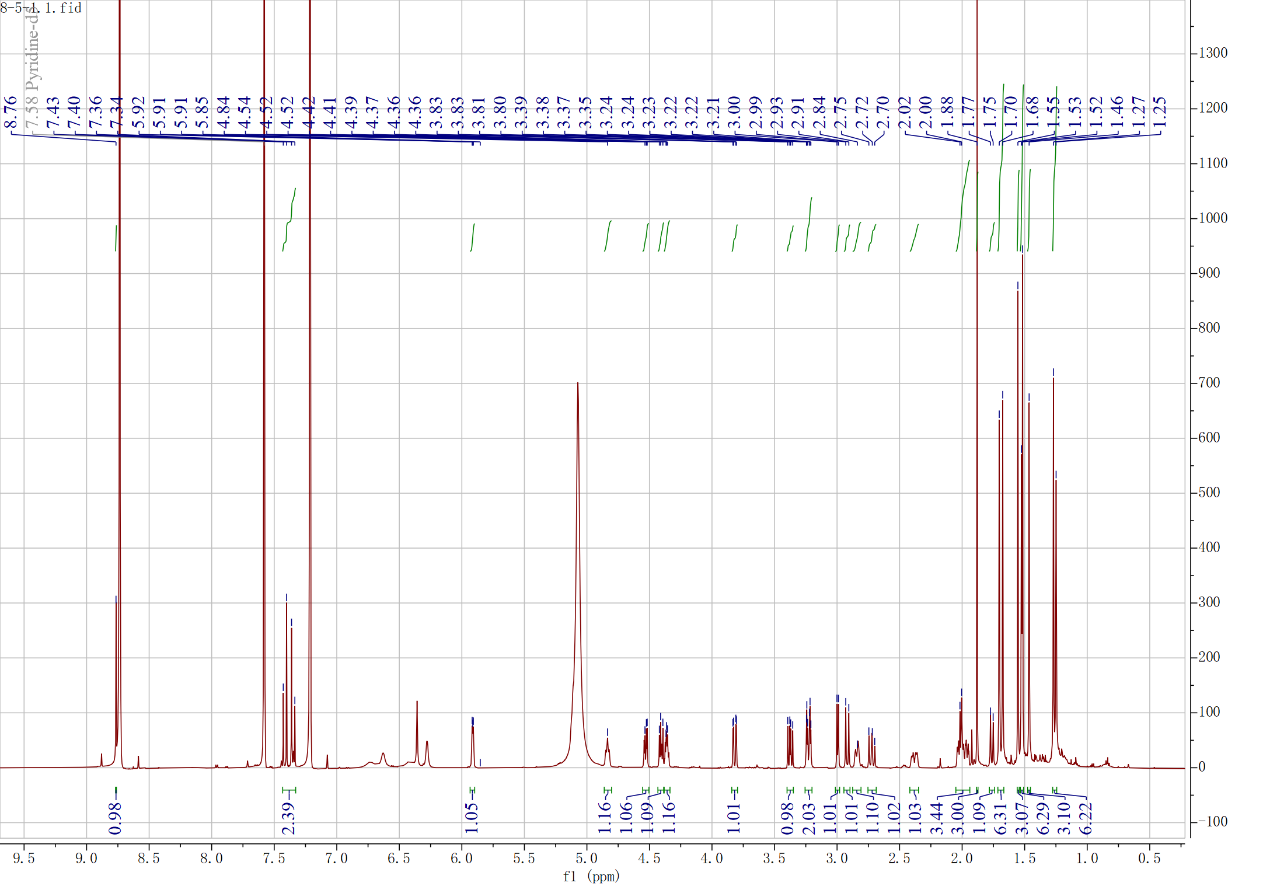


Figure S16. ^1^H-NMR spectrum (Pyrdine-D_5_, 600 MHz) of **2**


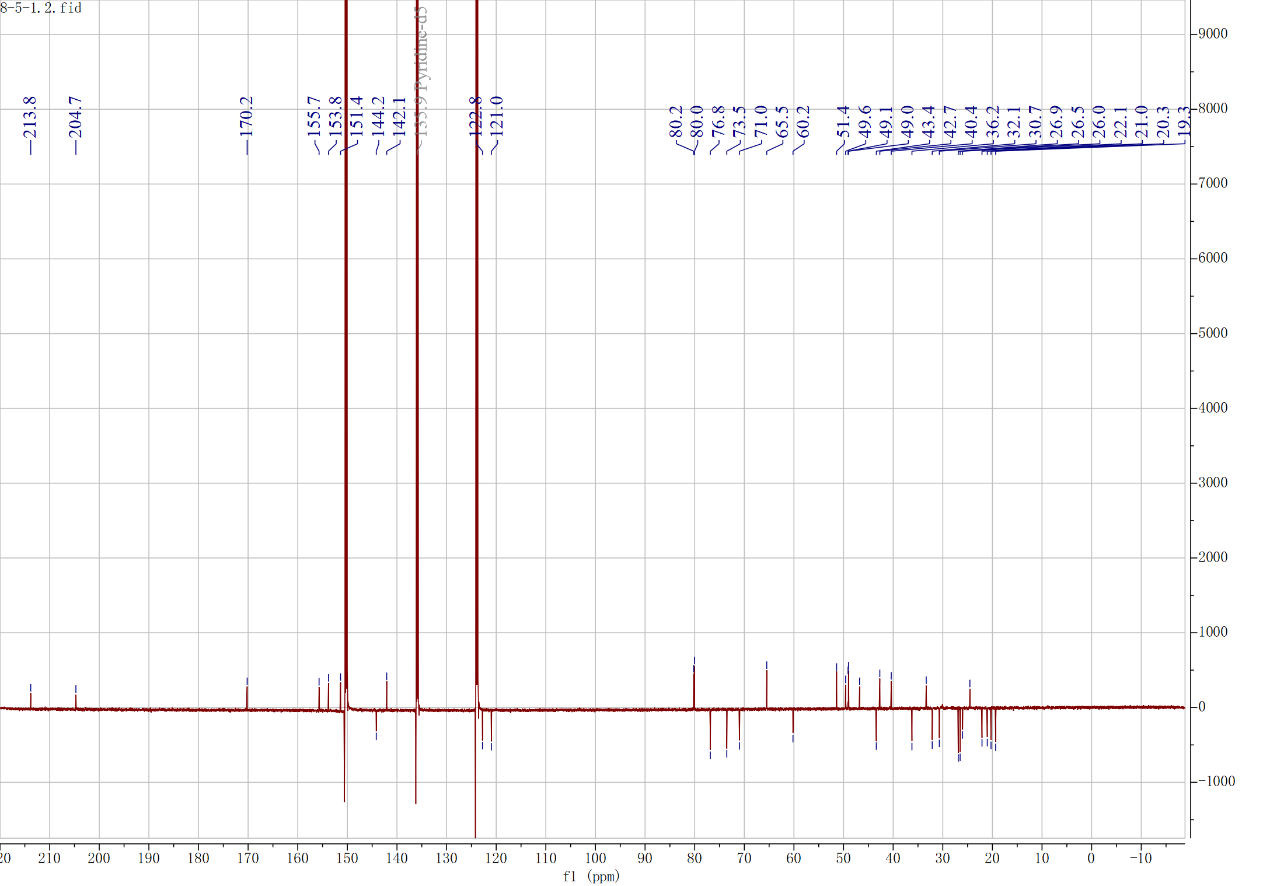


Figure S17. ^13^C-APT NMR spectrum (Pyrdine-D_5_, 125 MHz) of **2**


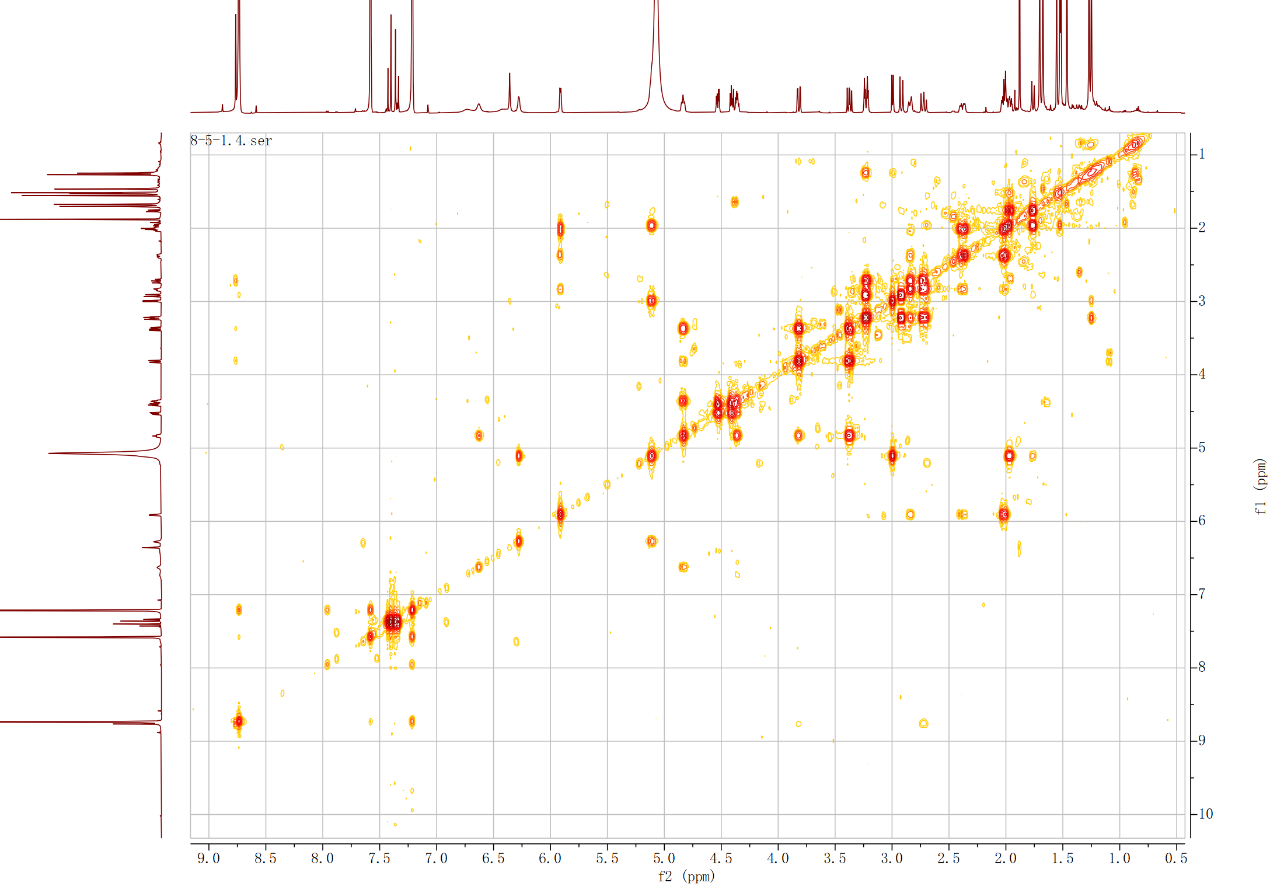


Figure S18. ^1^H-^1^H COSY spectrum (Pyrdine-D_5_) of **2**


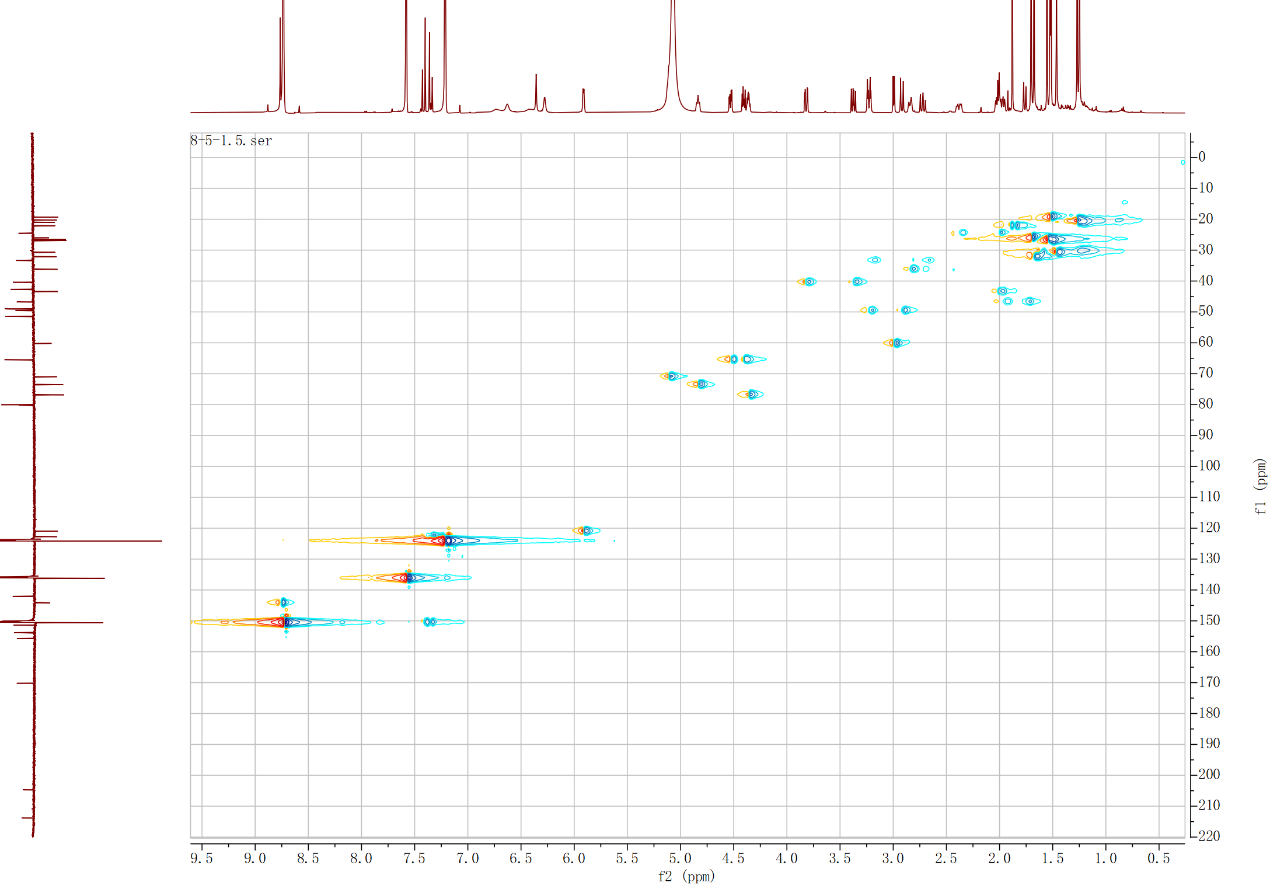


Figure S19. HSQC spectrum (Pyrdine-D_5_) of **2**


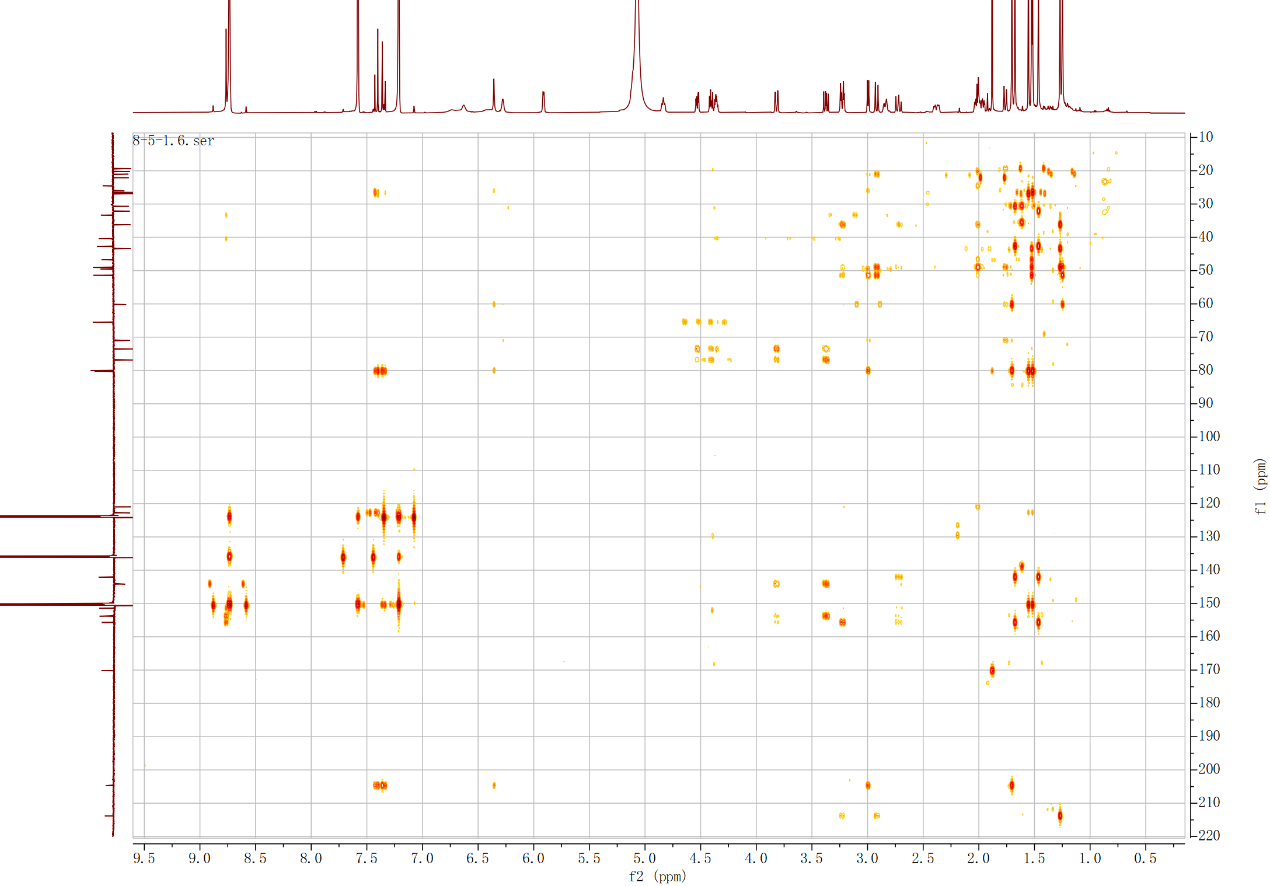


Figure S20. HMBC spectrum (Pyrdine-D_5_) of **2**


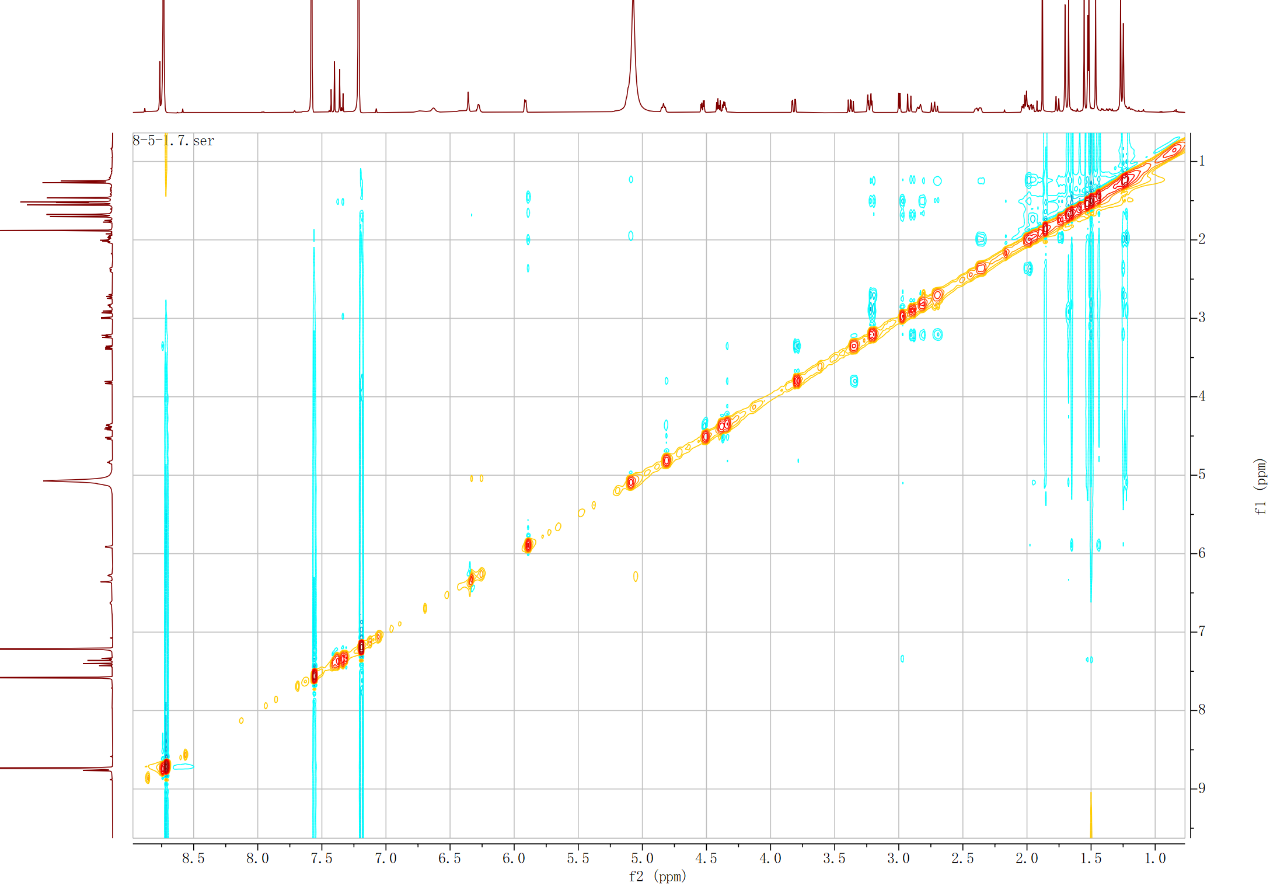


Figure S21. NOESY spectrum (Pyrdine-D_5_) of **2**

**MS, IR, UV, ECD and NMR Spectra of Compound 3**

Figure S22. HR-ESI-MS spectrum of **3**


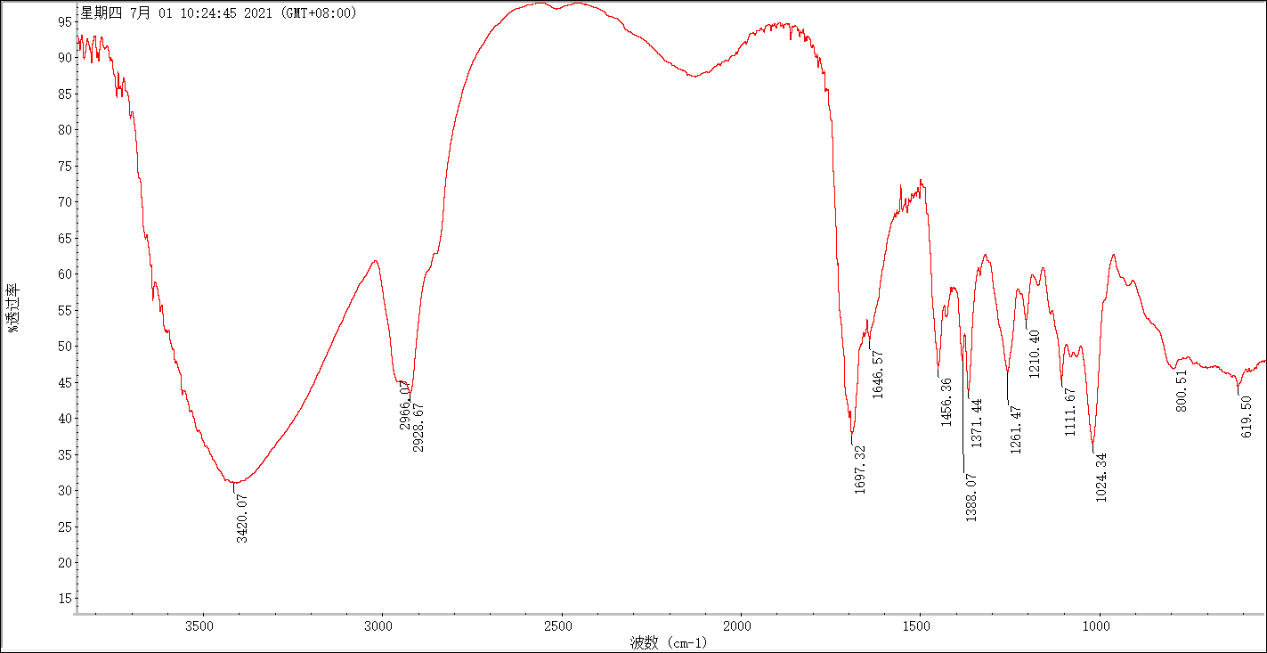


Figure S23. IR spectrum of **3**

Figure S24. UV spectrum in MeOH of **3**


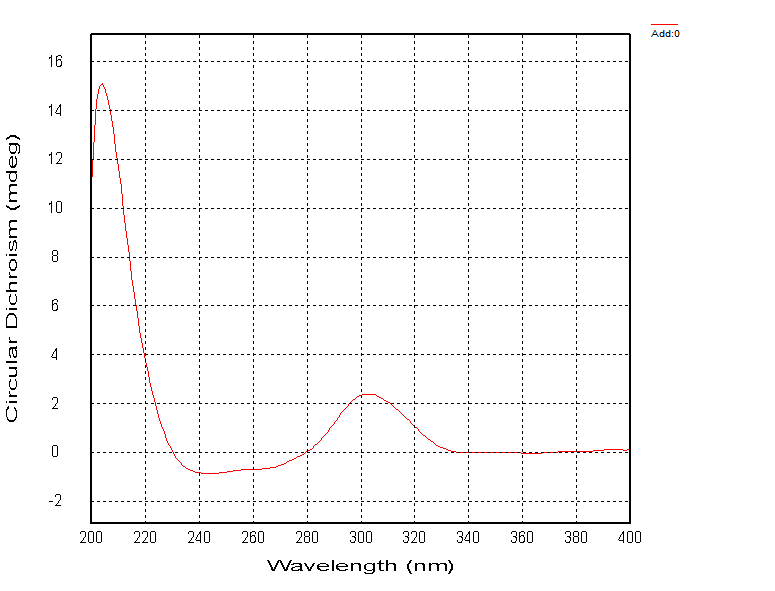


Figure S25. ECD spectrum in MeOH of **3**


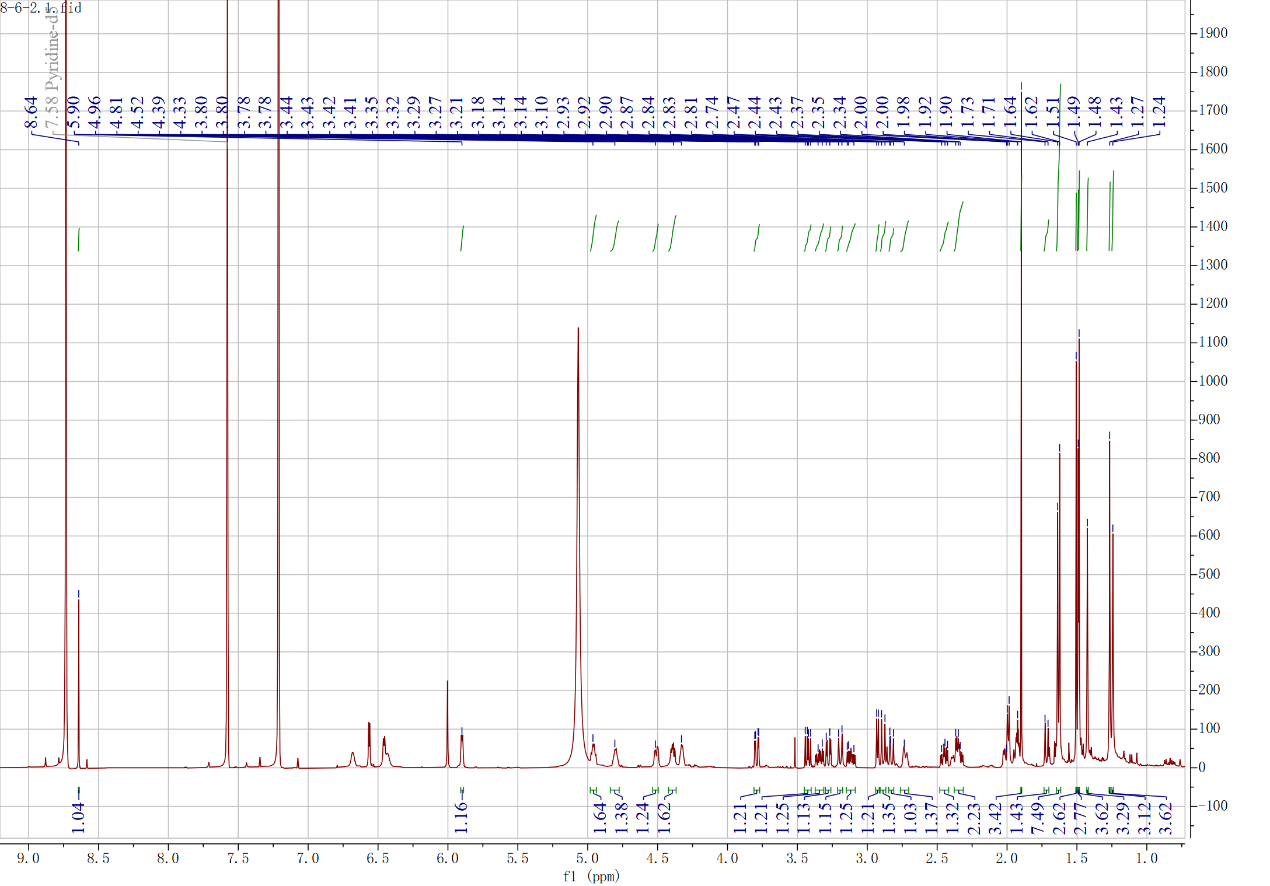


Figure S26. ^1^H-NMR spectrum (Pyrdine-D_5_, 600 MHz) of **3**


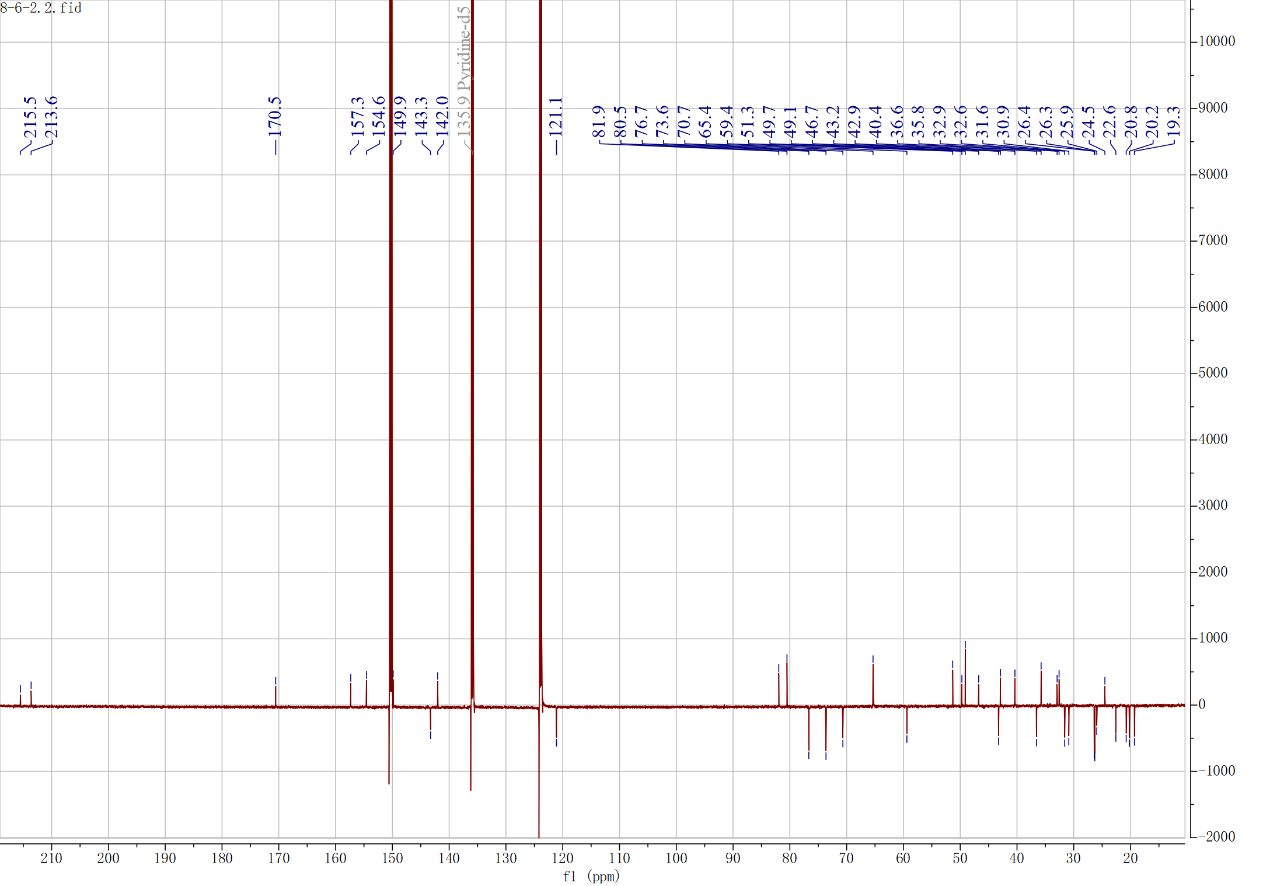


Figure S27. ^13^C-APT NMR spectrum (Pyrdine-D_5_, 125 MHz) of **3**


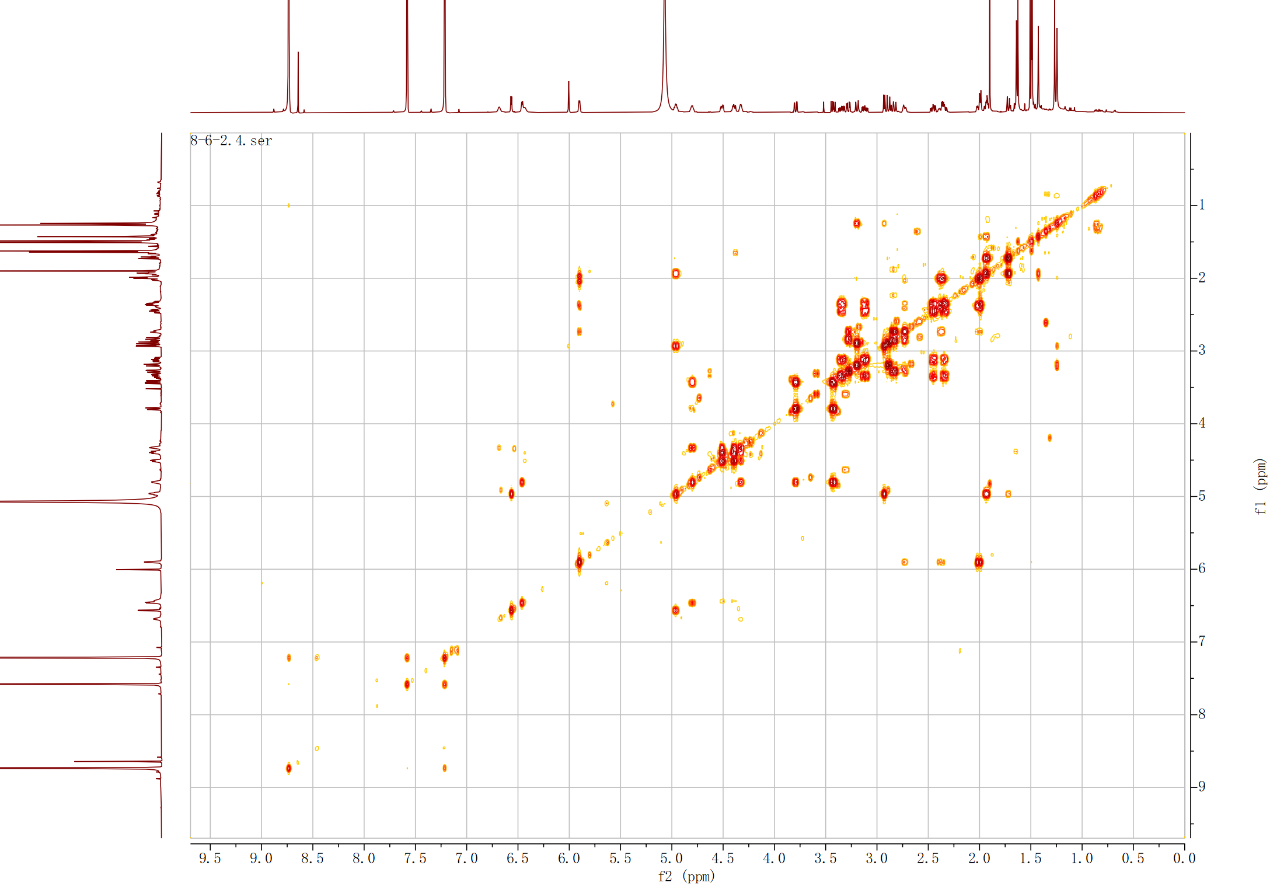


Figure S28. ^1^H-^1^H COSY spectrum (Pyrdine-D_5_) of **3**


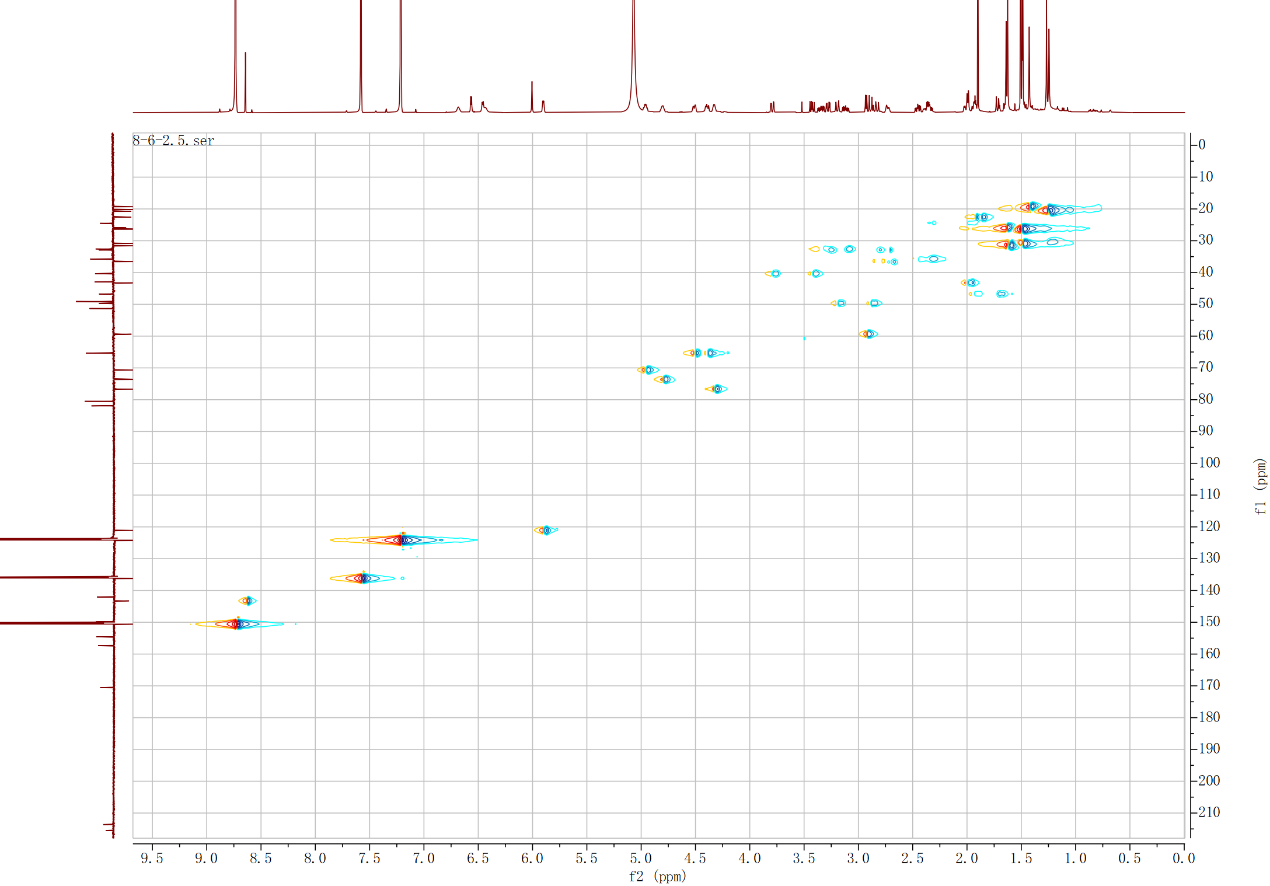


Figure S29. HSQC spectrum (Pyrdine-D_5_) of **3**


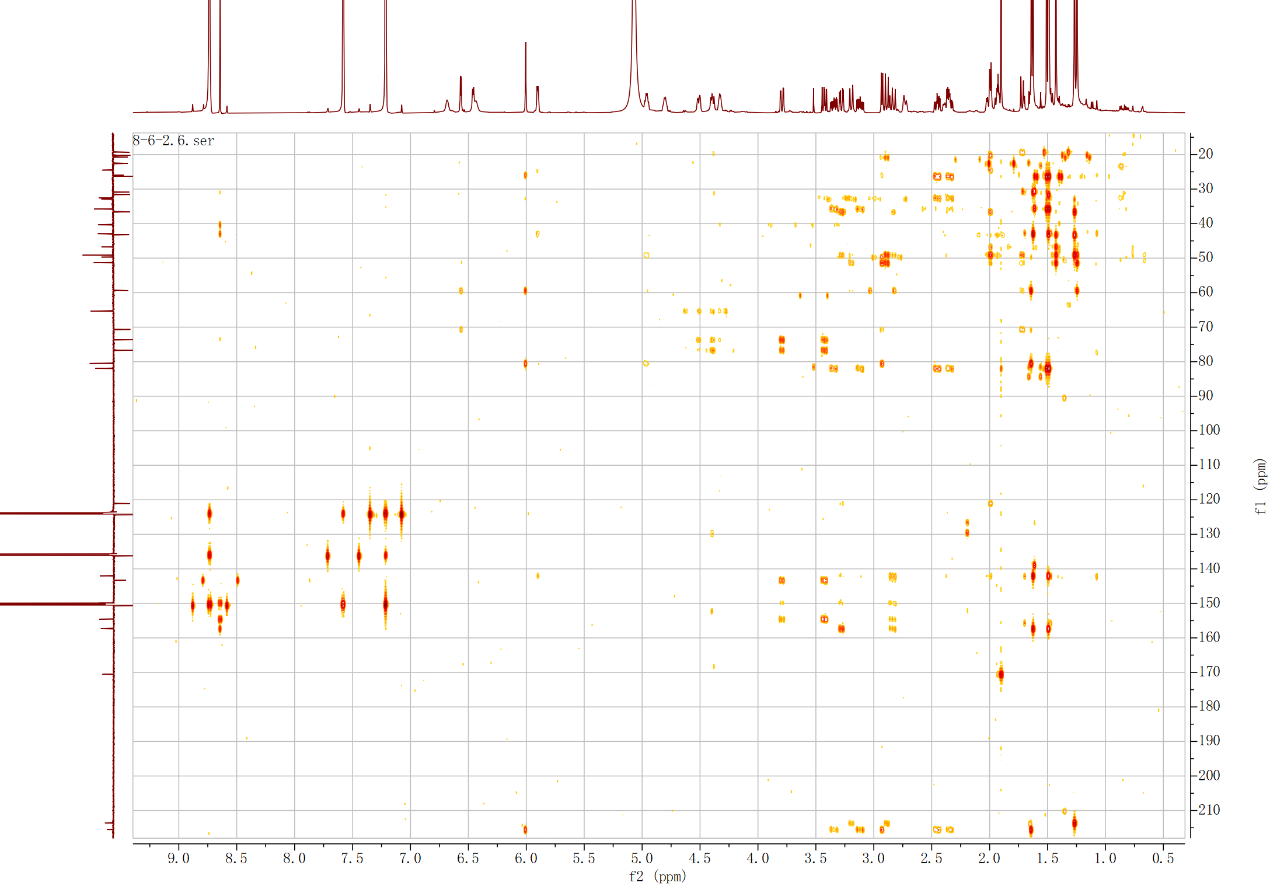


Figure S30. HMBC spectrum (Pyrdine-D_5_) of **3**


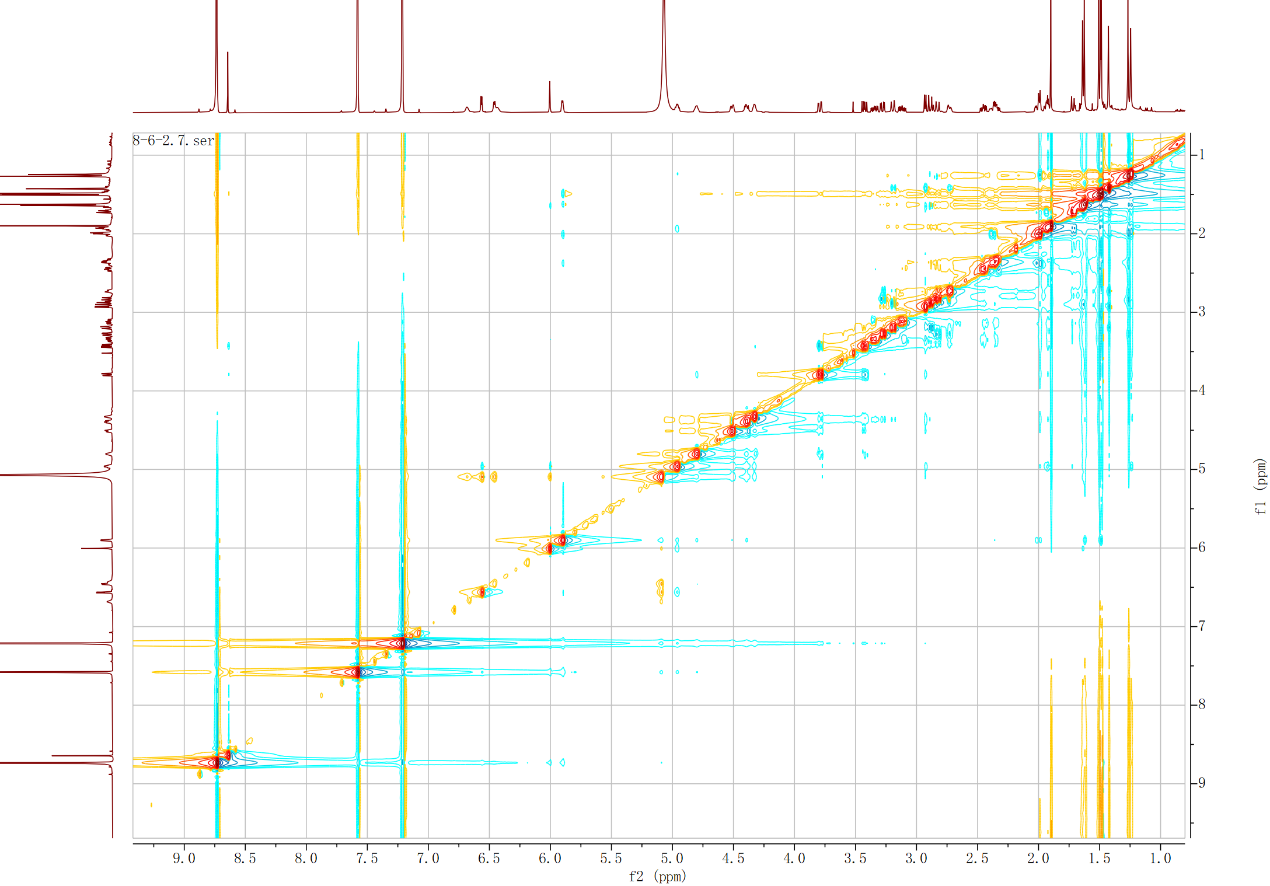


Figure S31. NOESY spectrum (Pyrdine-D_5_) of **3**

**
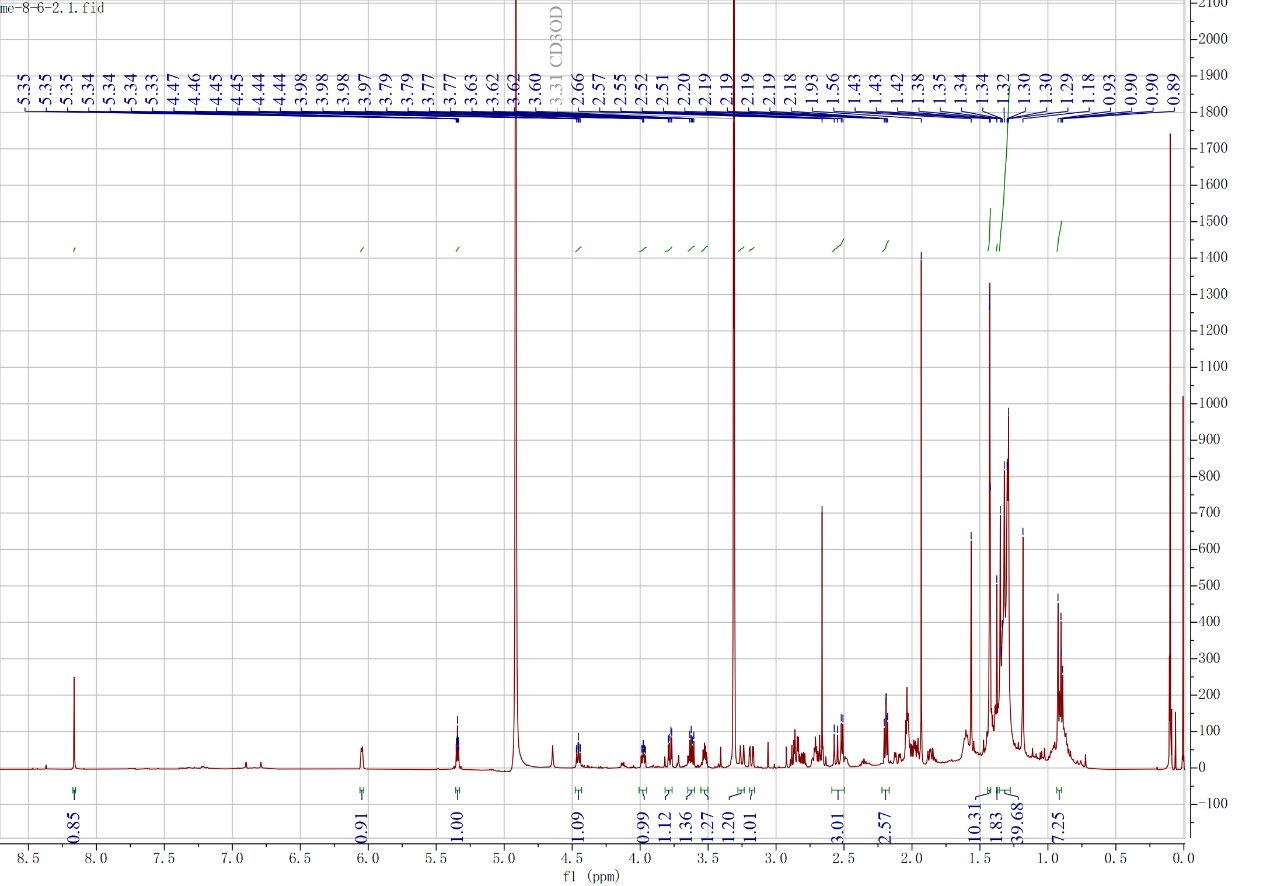
**Figure S32. ^1^H-NMR spectrum (CD_3_OD, 600 MHz) of **3**

**MS, IR, UV, ECD and NMR Spectra of Compound 4**

Figure S33. HR-ESI-MS spectrum of **4**


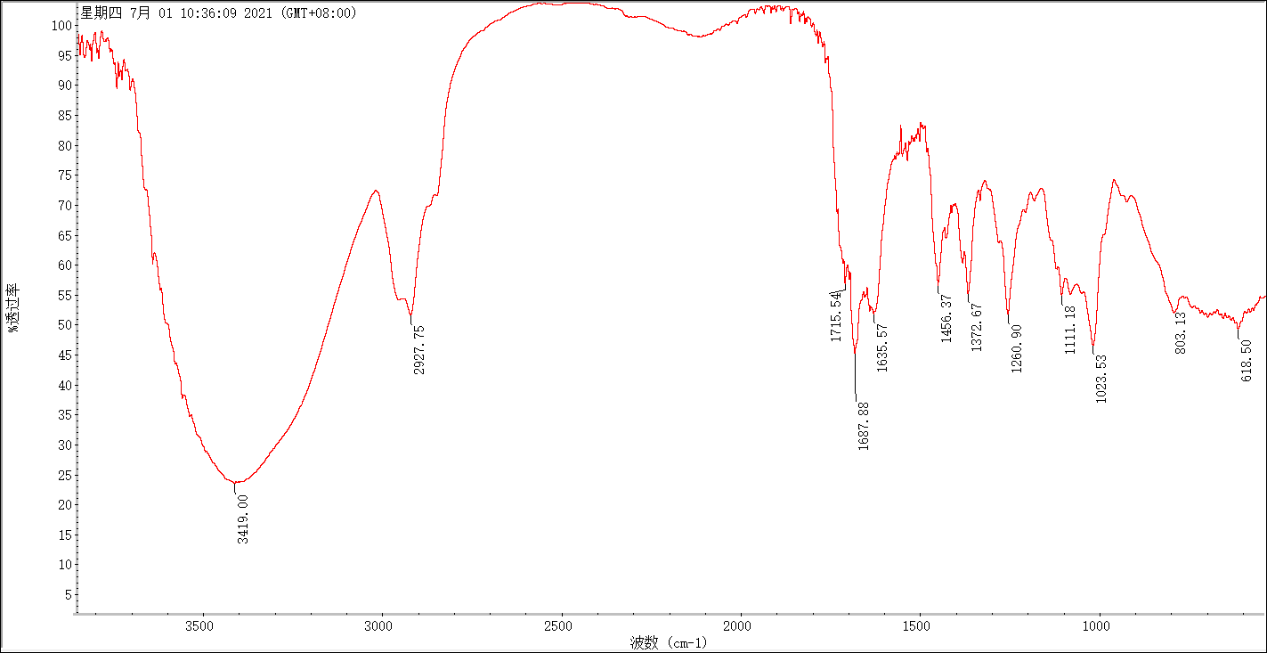


Figure S34. IR spectrum of **4**

Figure S35. UV spectrum in MeOH of **4**


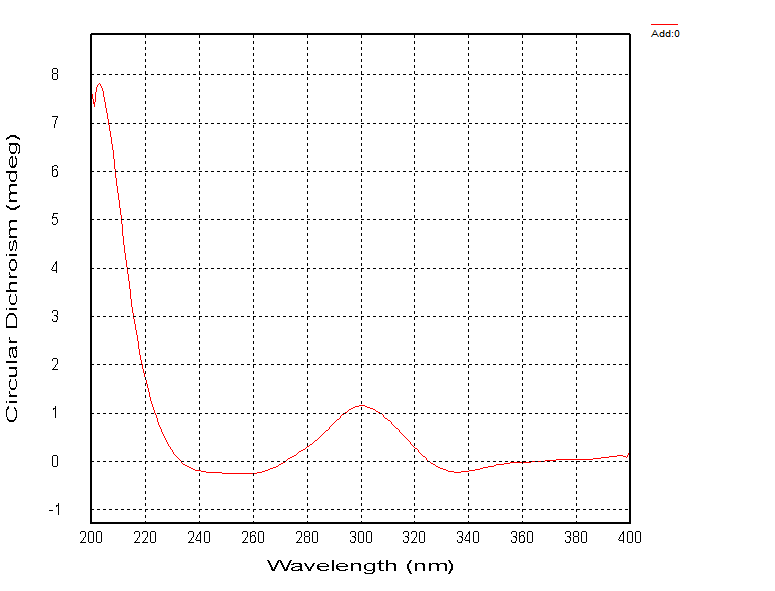


Figure S36. ECD spectrum in MeOH of **4**


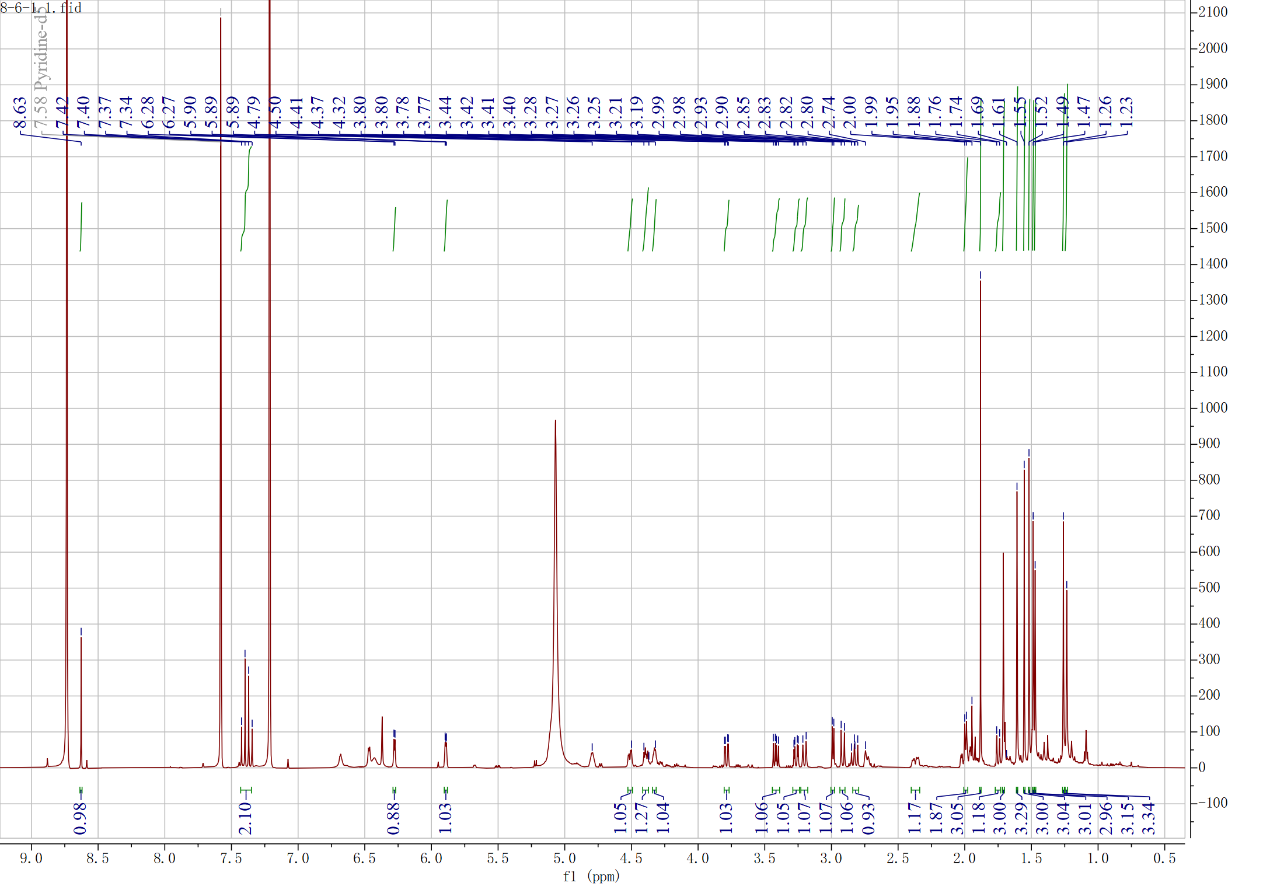


Figure S37. ^1^H-NMR spectrum (Pyrdine-D_5_, 600 MHz) of **4**


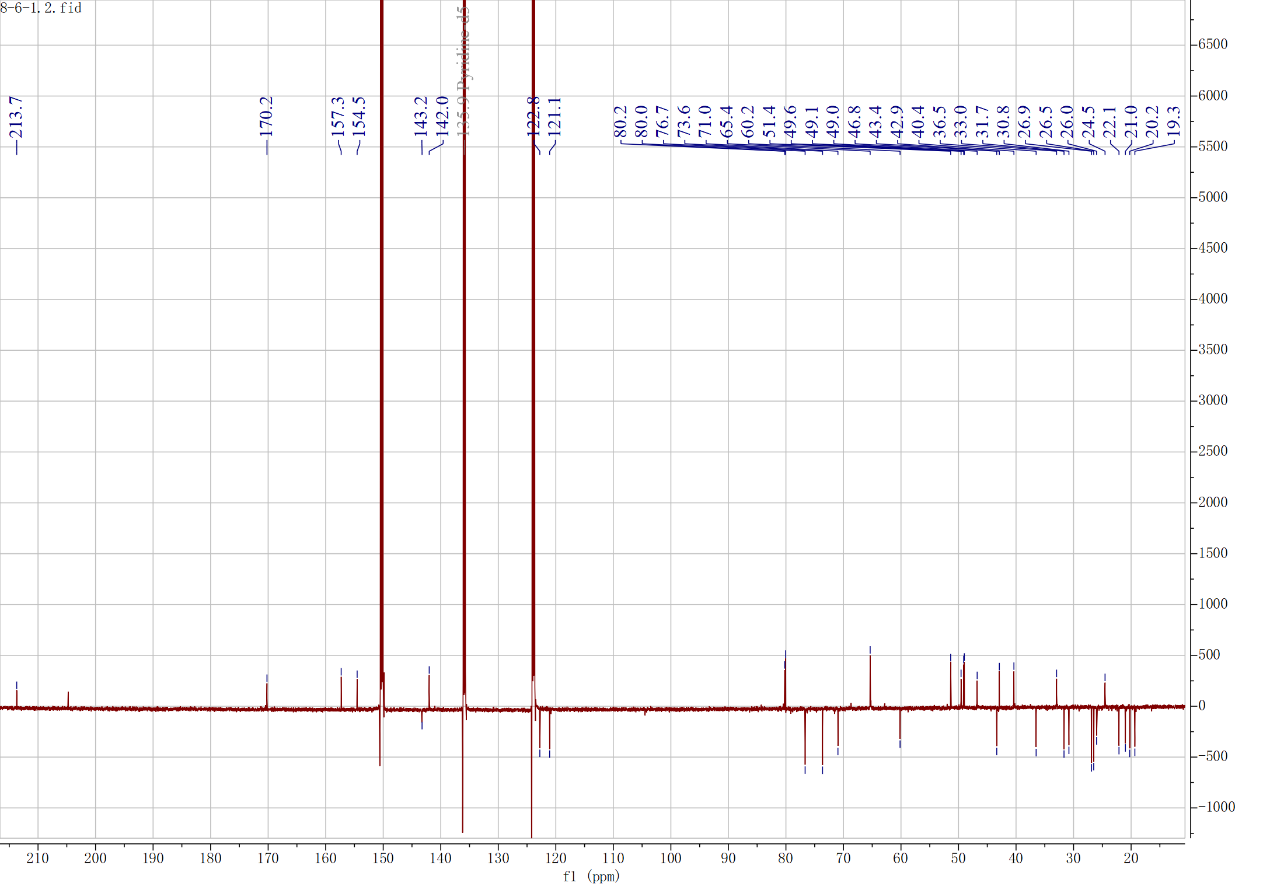


Figure S38. ^13^C-APT NMR spectrum (Pyrdine-D_5_-D5, 125 MHz) of **4**


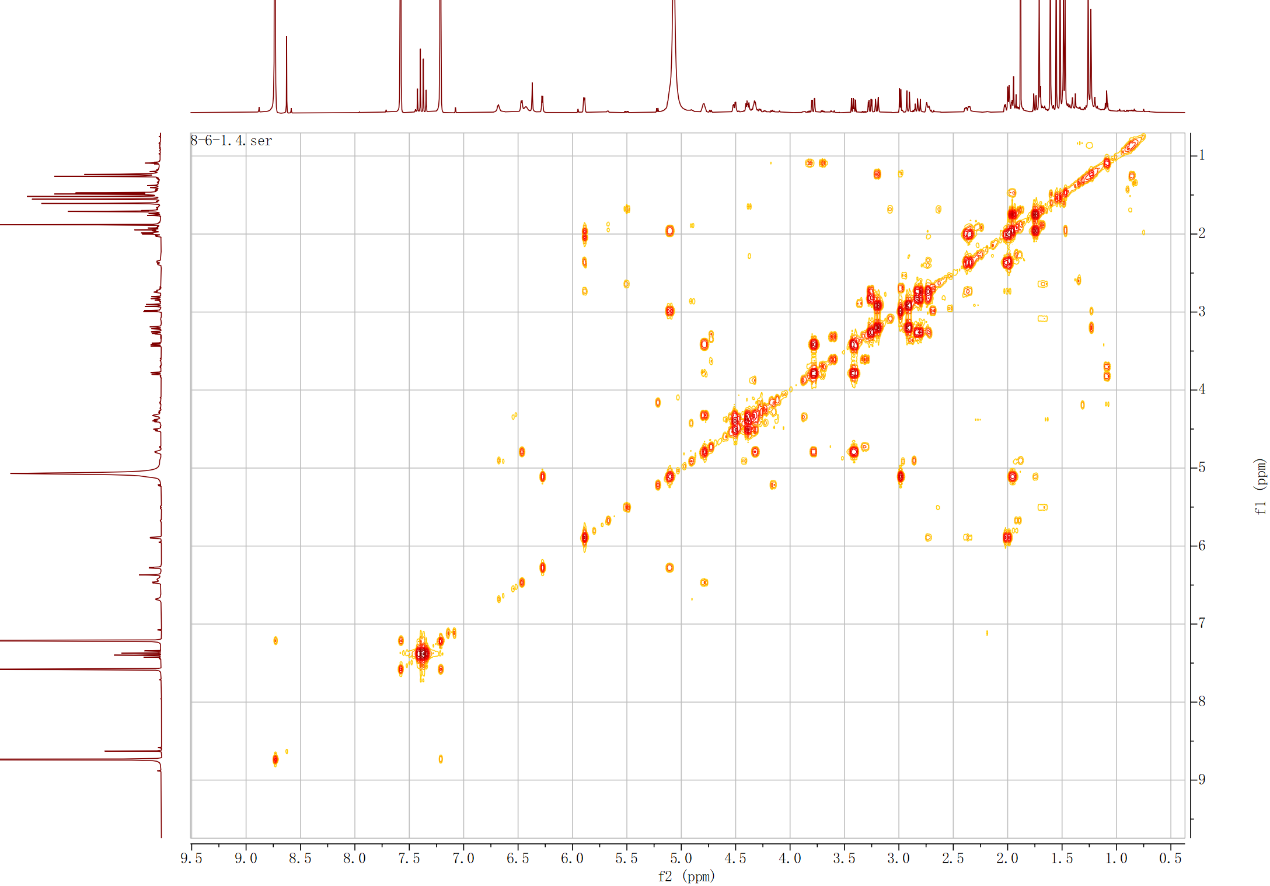


Figure S39. ^1^H-^1^H COSY spectrum (Pyrdine-D_5_) of **4**


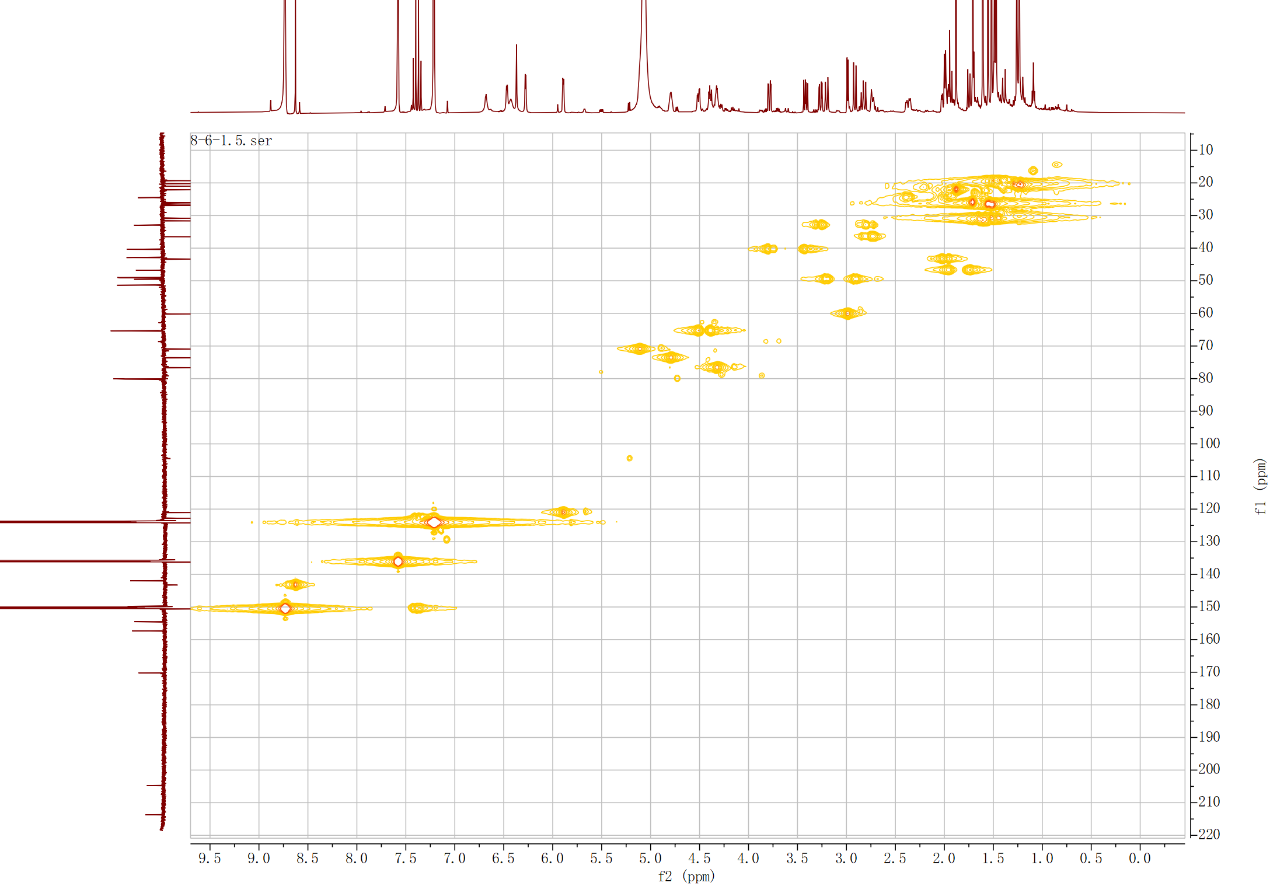


Figure S40. HSQC spectrum (Pyrdine-D_5_) of **4**


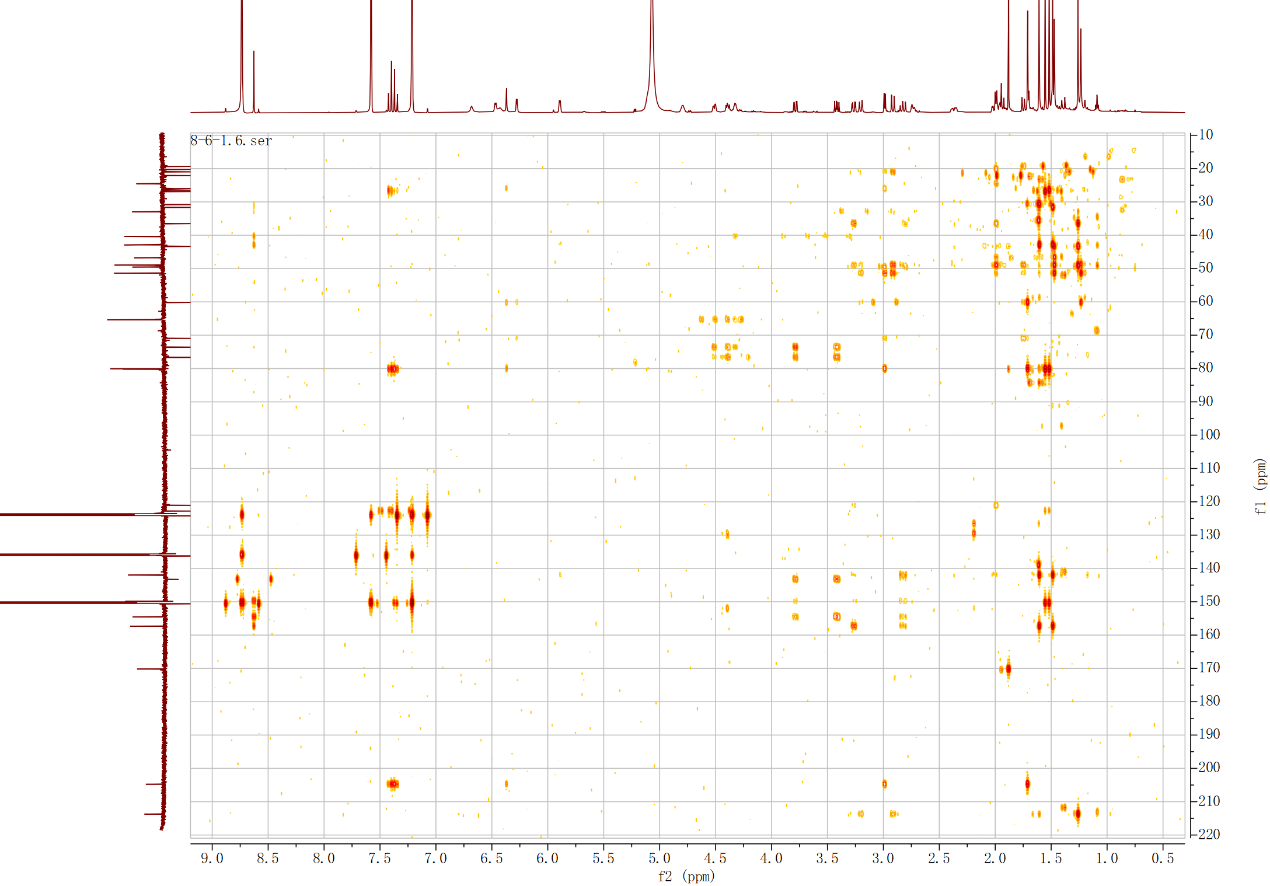


Figure S41. HMBC spectrum (Pyrdine-D_5_) of **4**


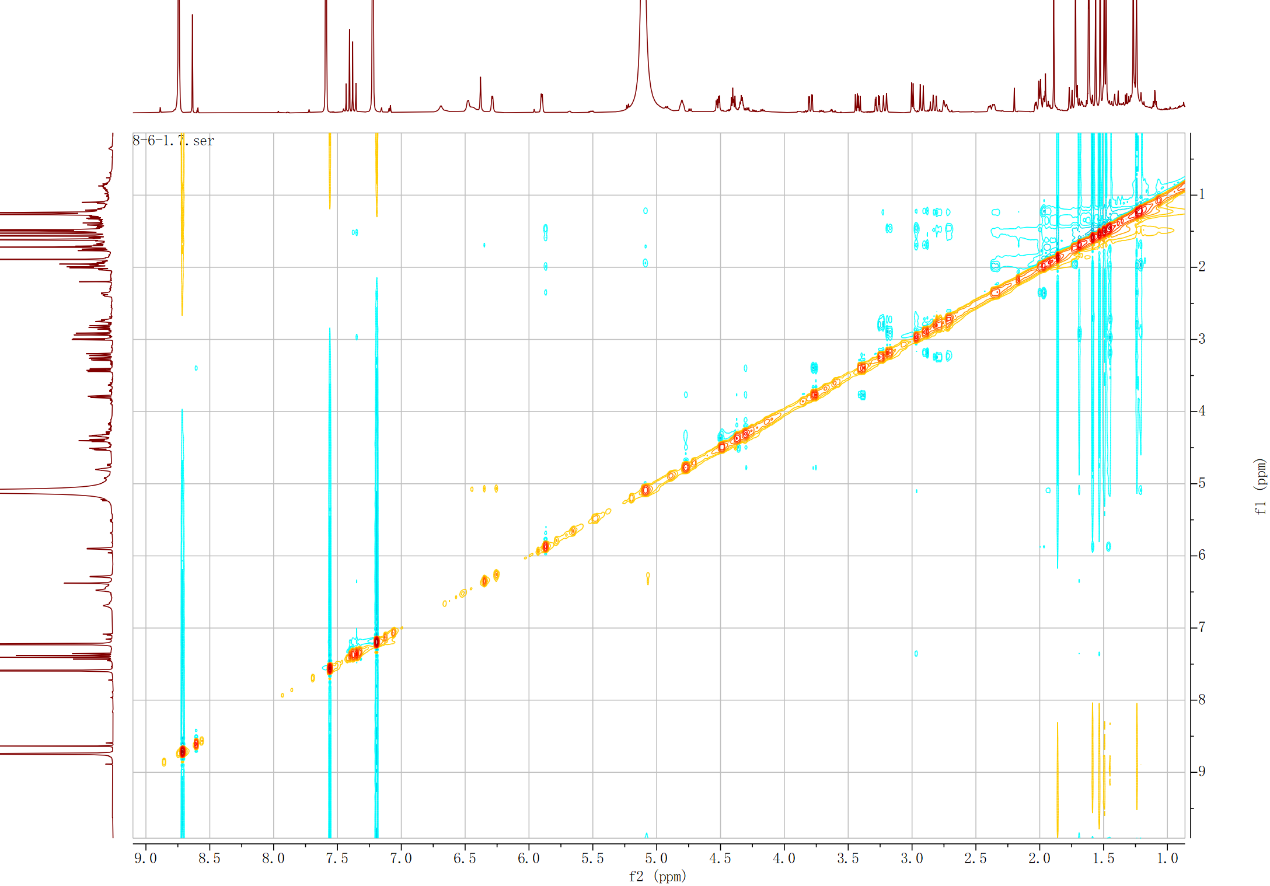


Figure S42. NOESY spectrum (Pyrdine-D_5_) of **4**

**MS, IR, UV, ECD and NMR Spectra of Compound 5**

Figure S43. HR-ESI-MS spectrum of **5**


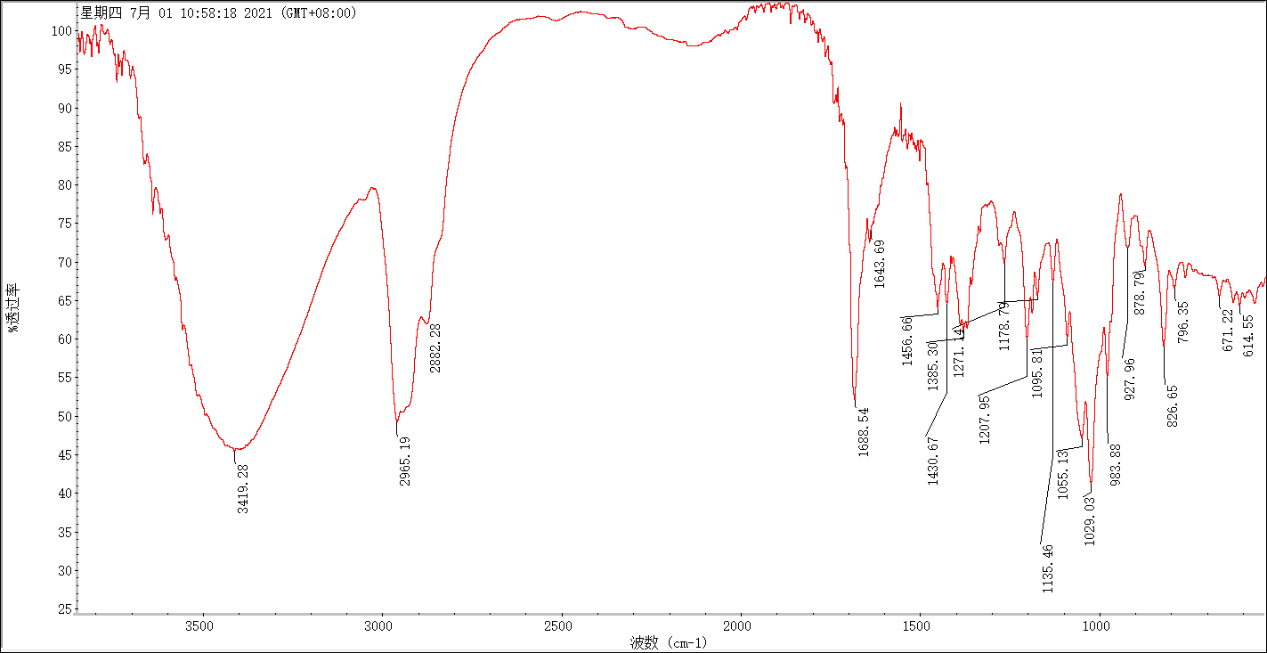


Figure S44. IR spectrum of **5**

Figure S45. UV spectrum in MeOH of **5**


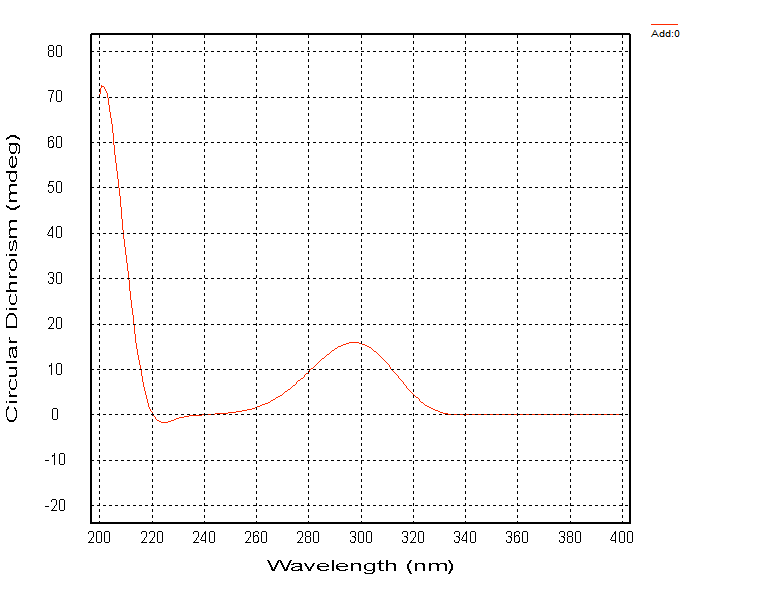


Figure S46. ECD spectrum in MeOH of **5**


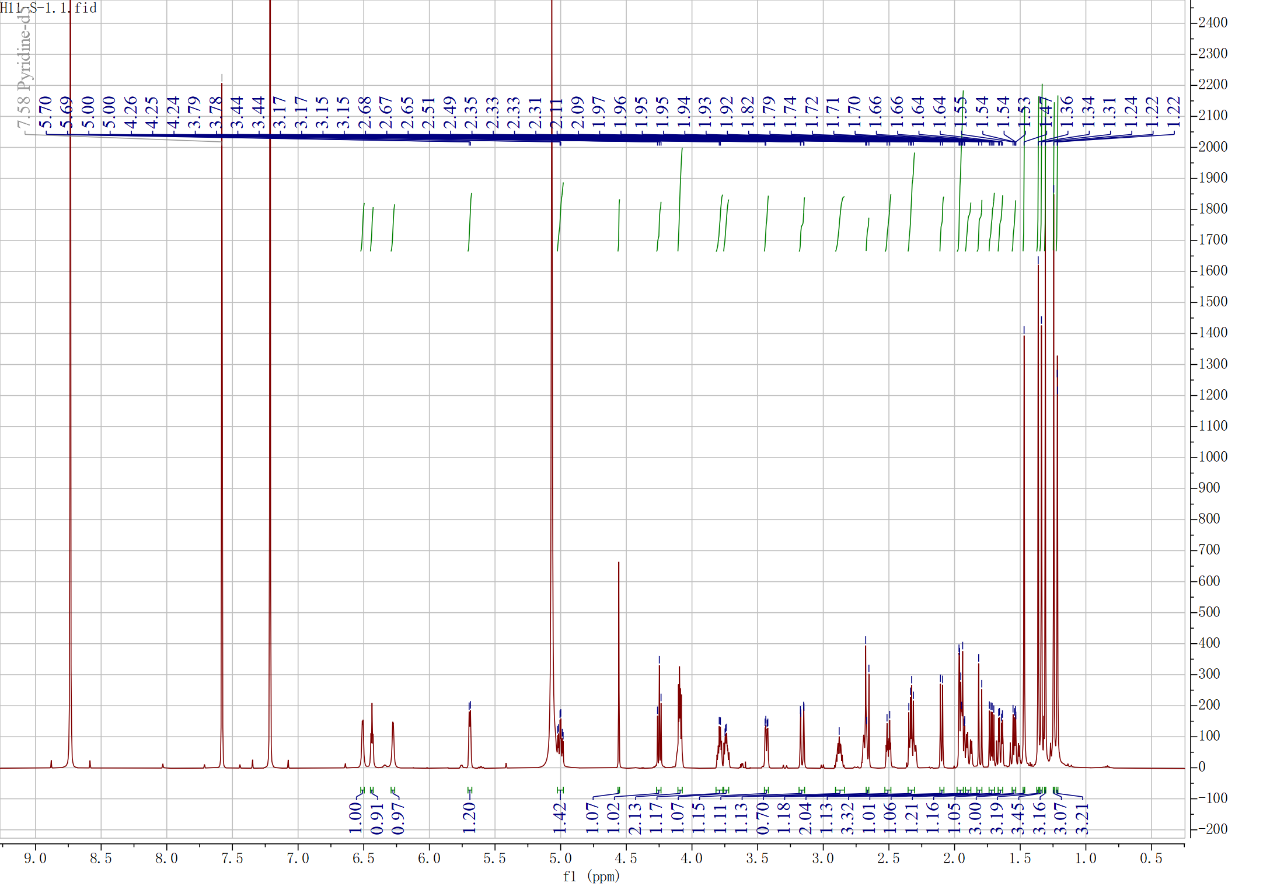


Figure S47. ^1^H-NMR spectrum (Pyrdine-D_5_, 600 MHz) of **5**


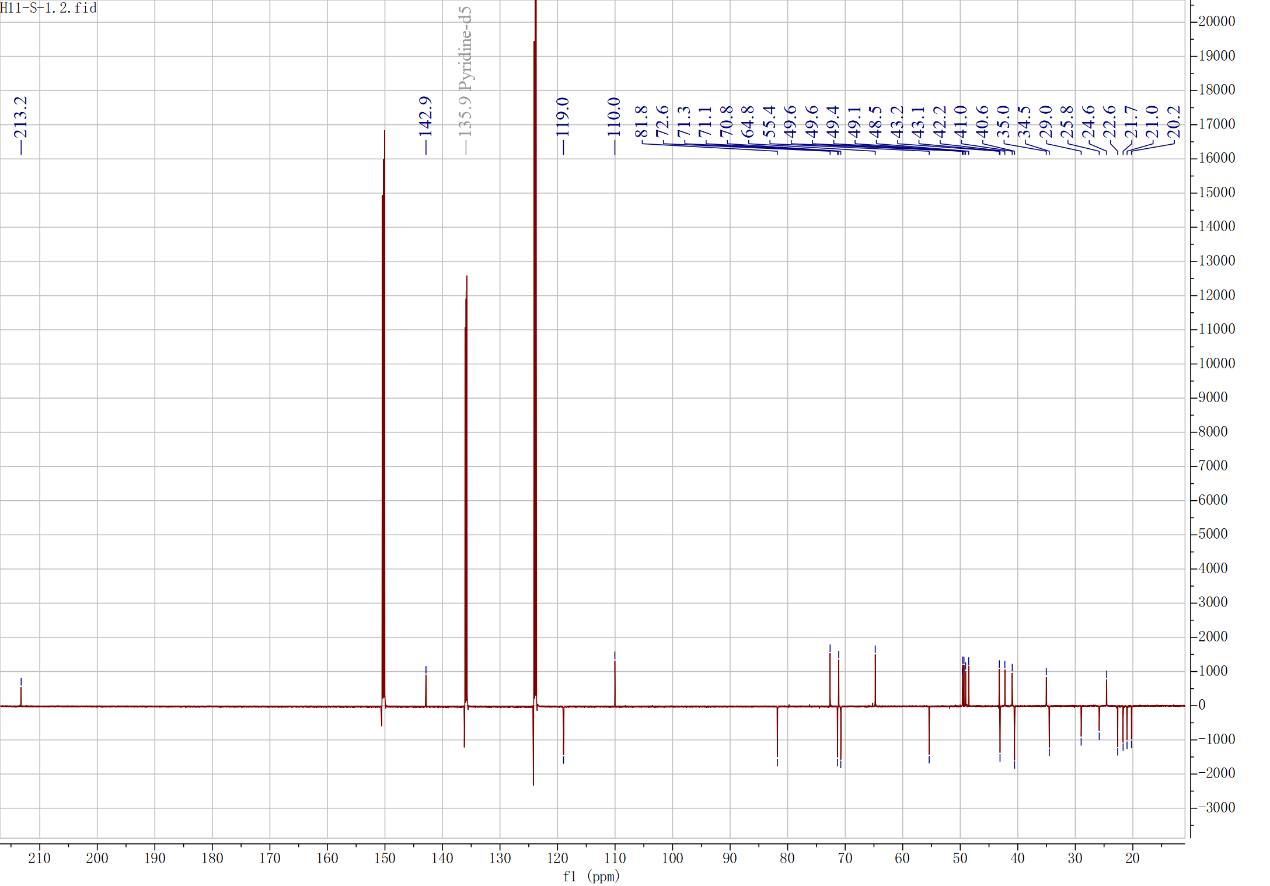


Figure S48. ^13^C-APT NMR spectrum (Pyrdine-D_5_, 125 MHz) of **5**


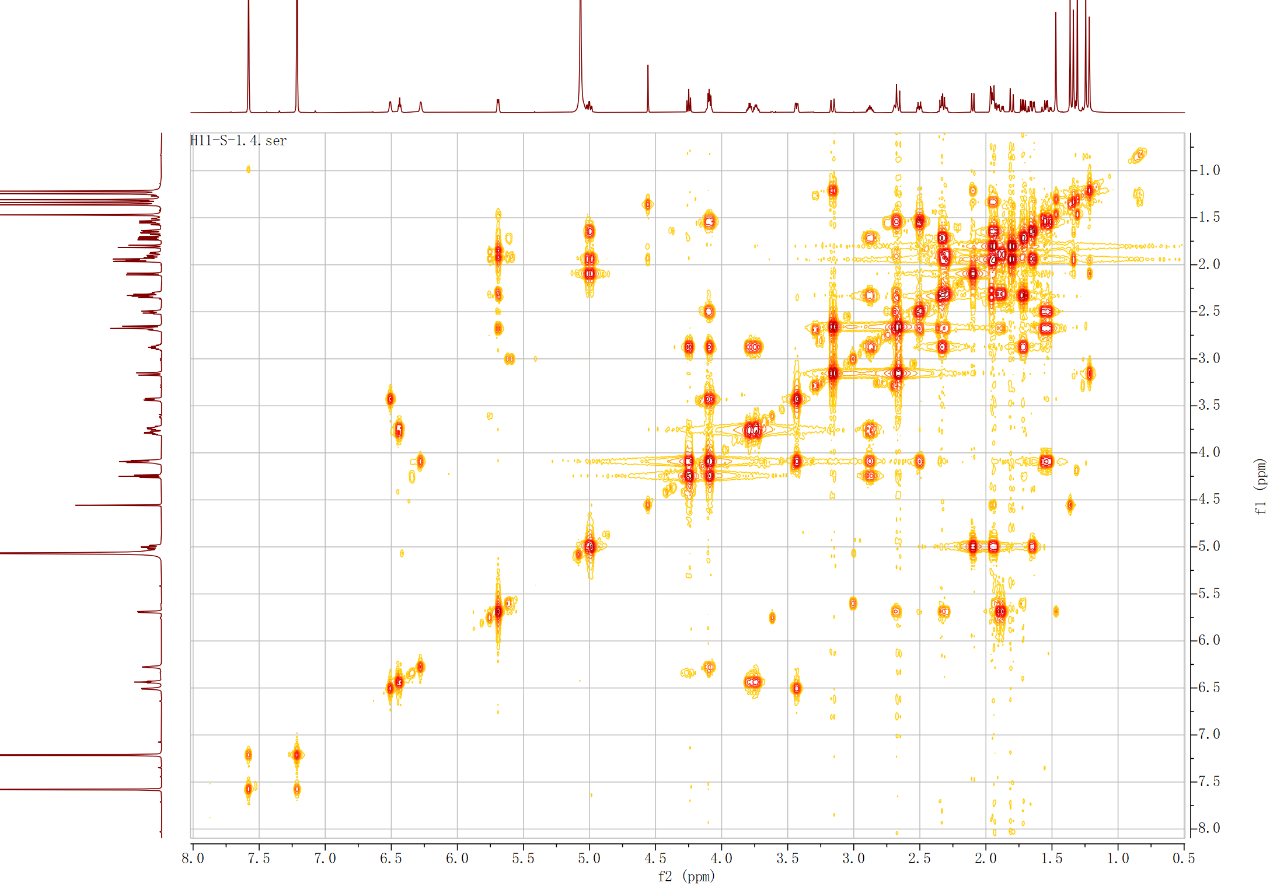


Figure S49. ^1^H-^1^H COSY spectrum (Pyrdine-D_5_) of **5**


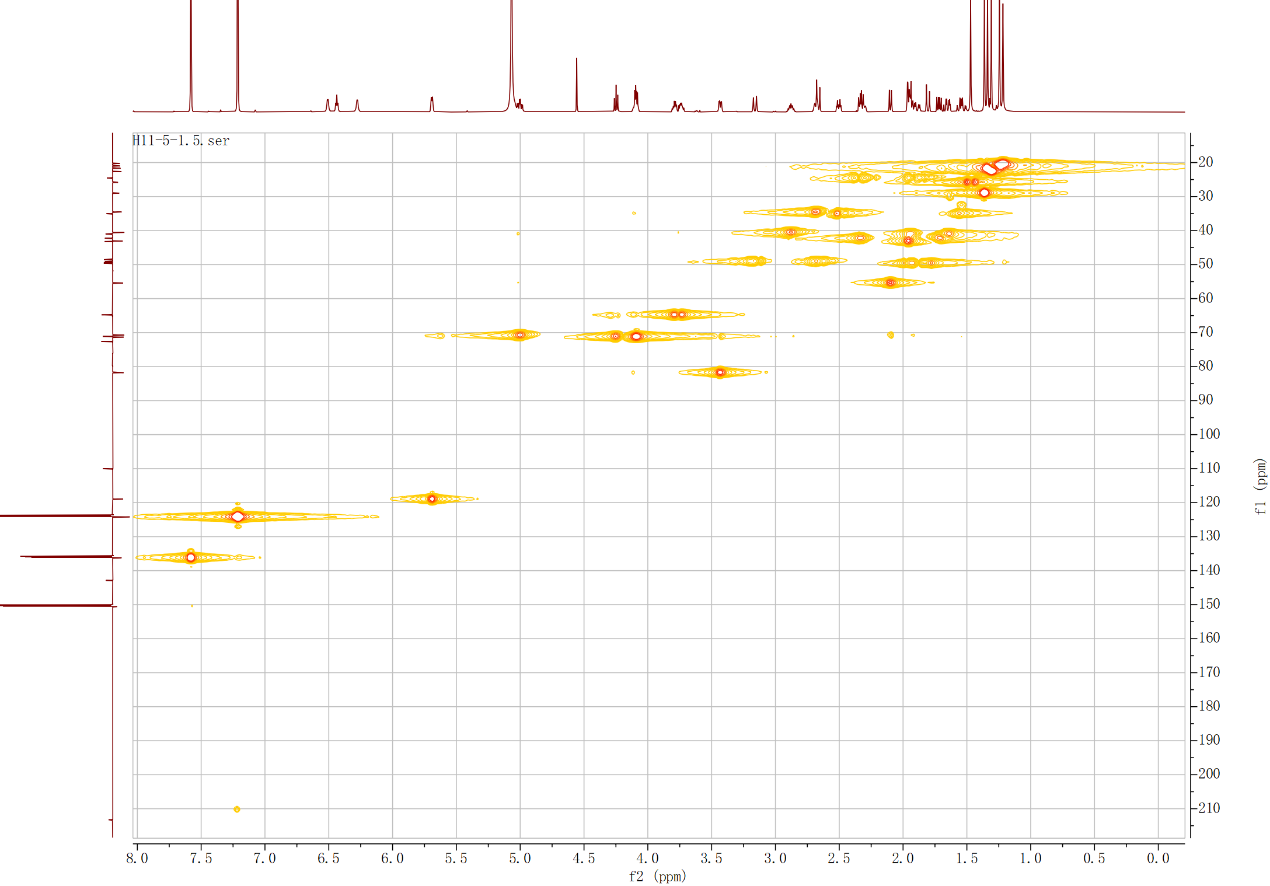


Figure S50. HSQC spectrum (Pyrdine-D_5_) of **5**


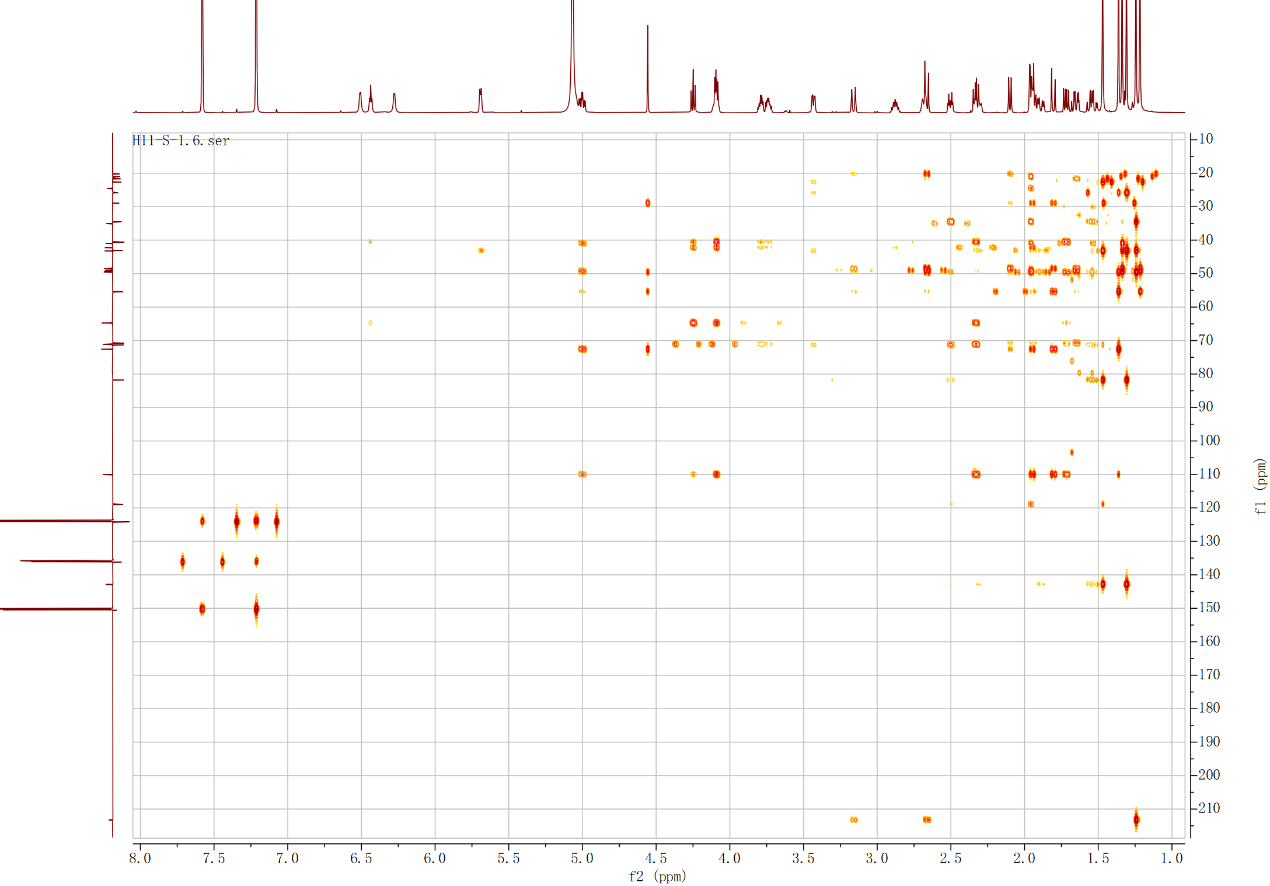


Figure S51. HMBC spectrum (Pyrdine-D_5_) of **5**


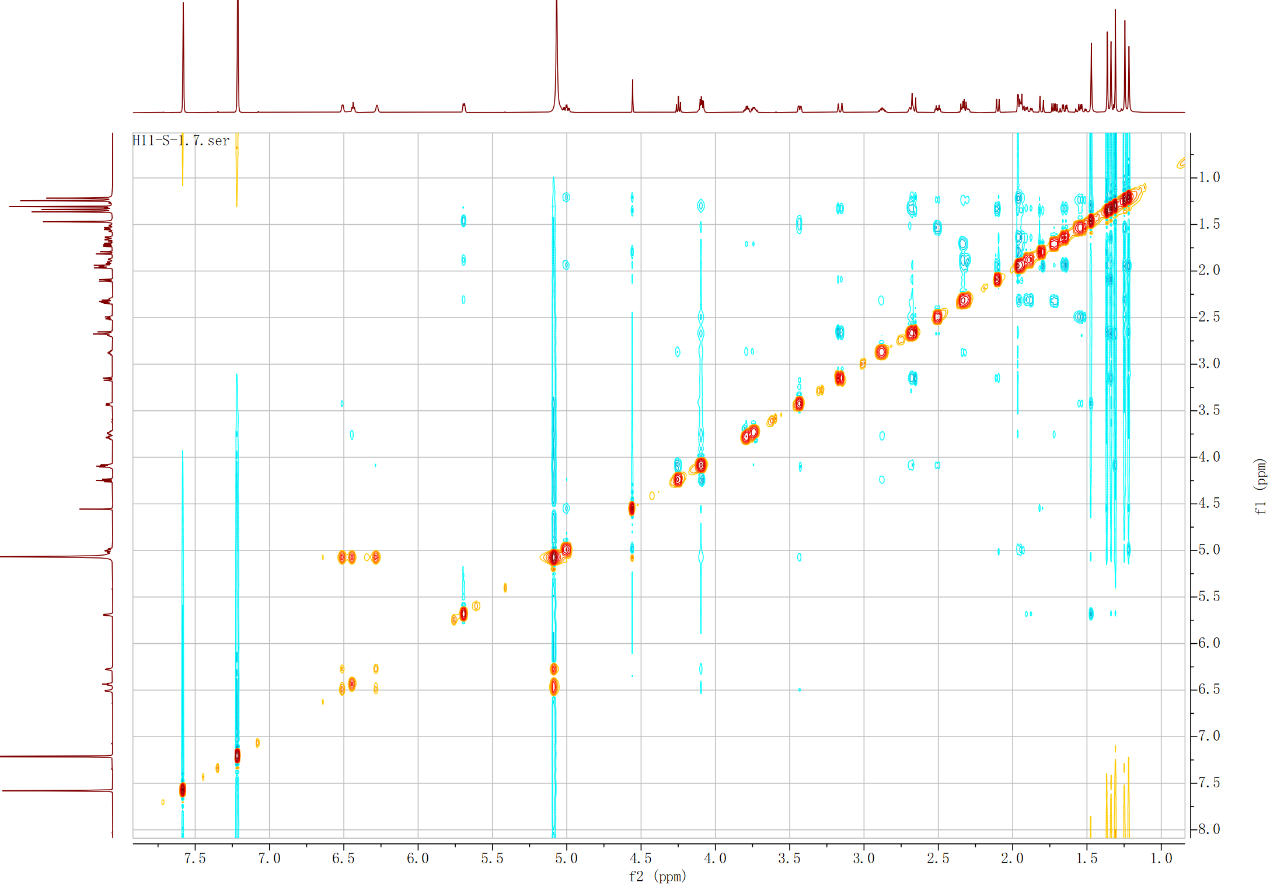


Figure S52. NOESY spectrum (Pyrdine-D_5_) of **5**

**MS, IR, UV, ECD and NMR Spectra of Compound 6**


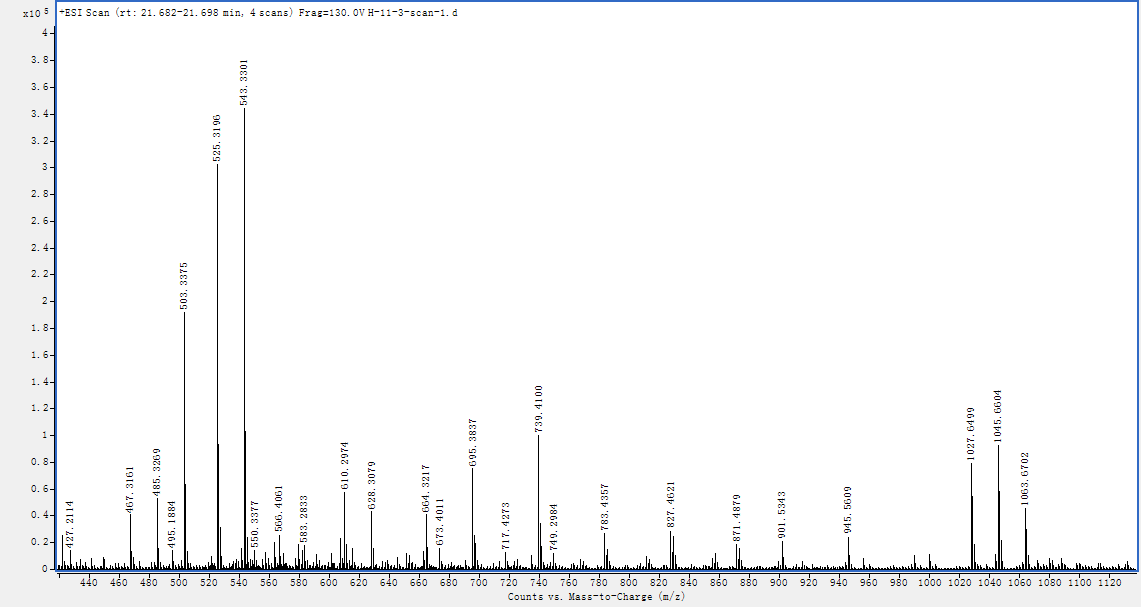


Figure S53. HR-ESI-MS spectrum of **6**


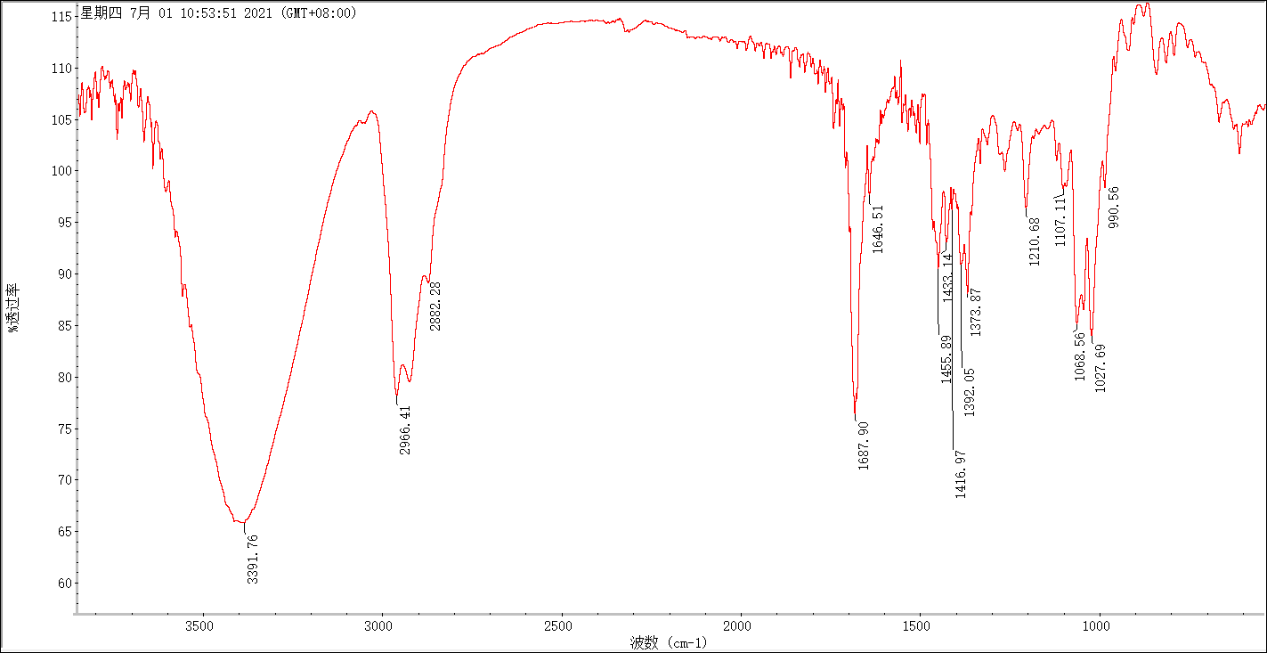


Figure S54. IR spectrum of **6**

Figure S55. UV spectrum in MeOH of **6**


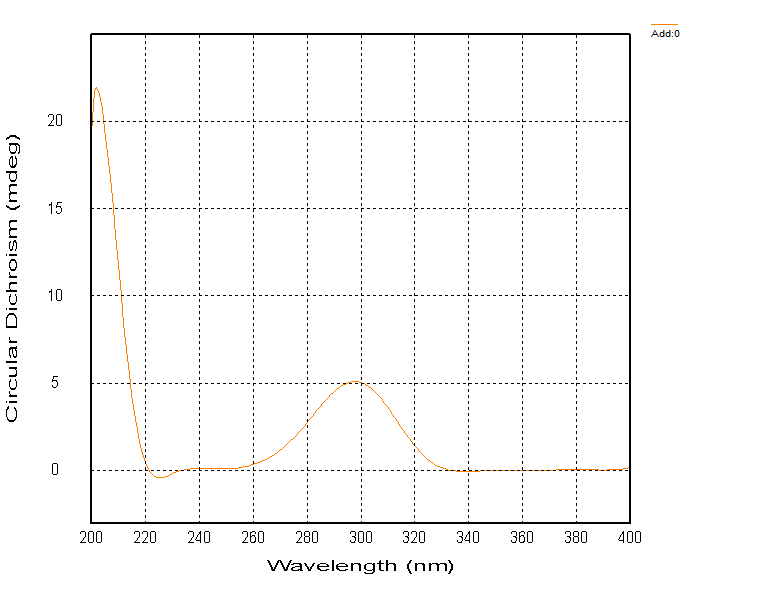


Figure S56. ECD spectrum in MeOH of **6**


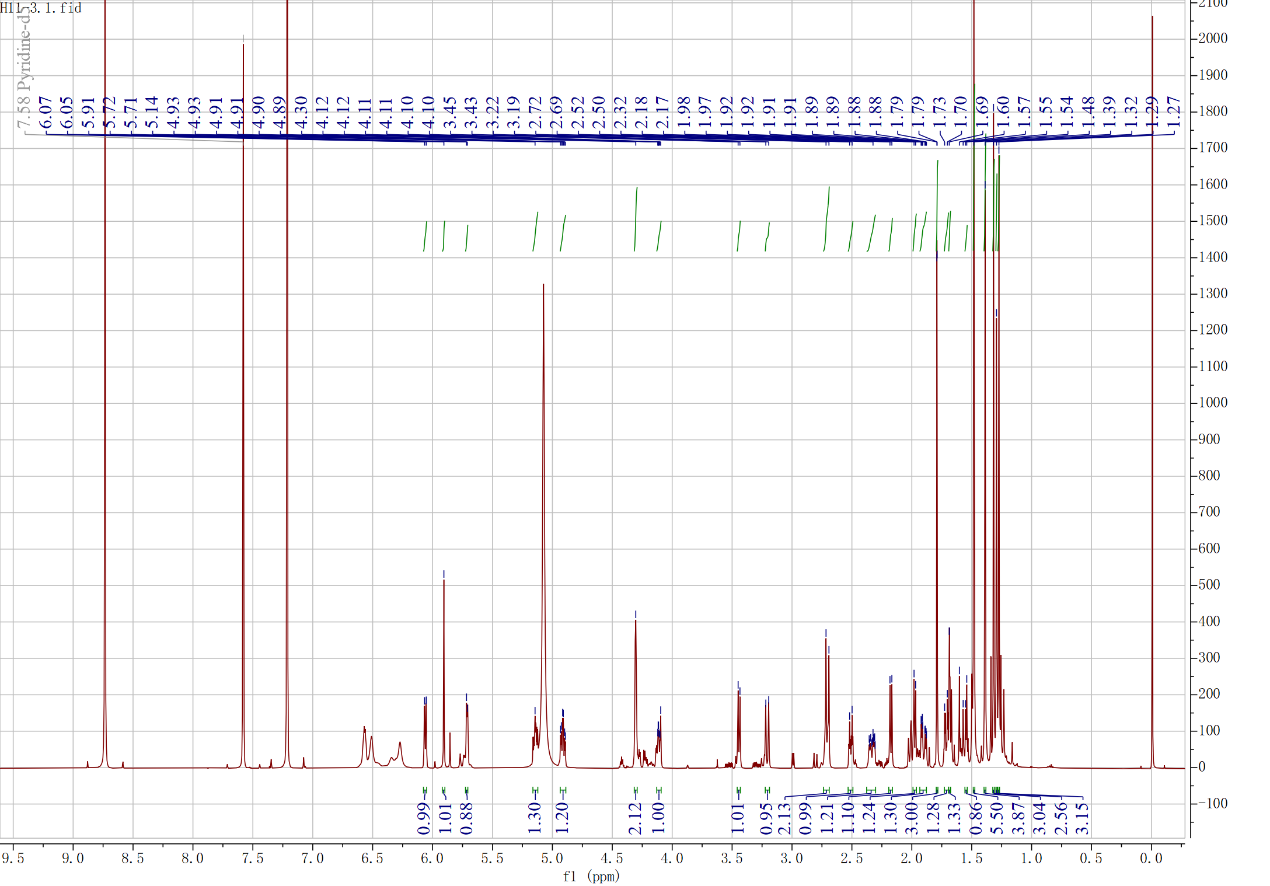


Figure S57. ^1^H-NMR spectrum (Pyrdine-D_5_, 600 MHz) of **6**


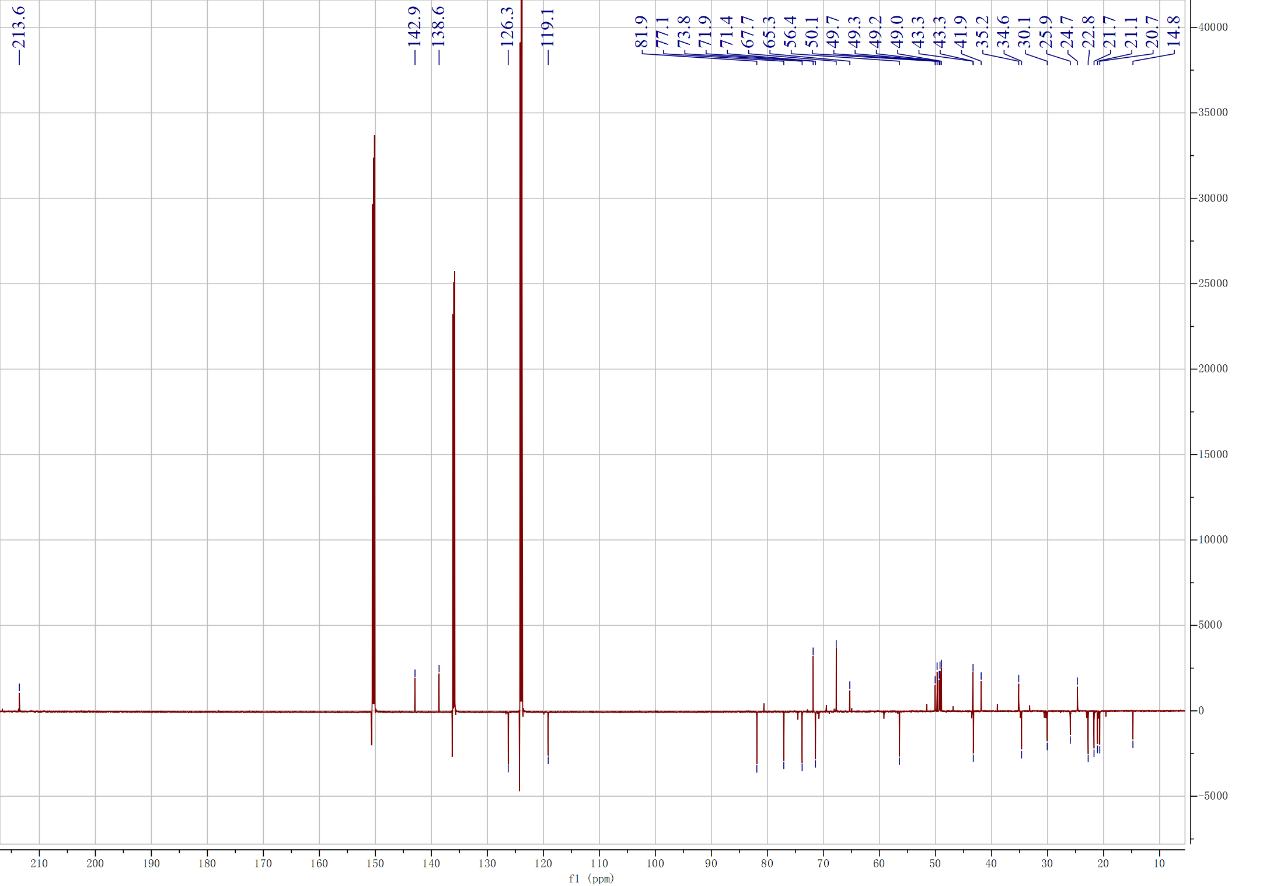


Figure S58. ^13^C-APT NMR spectrum (Pyrdine-D_5_, 125 MHz) of **6**


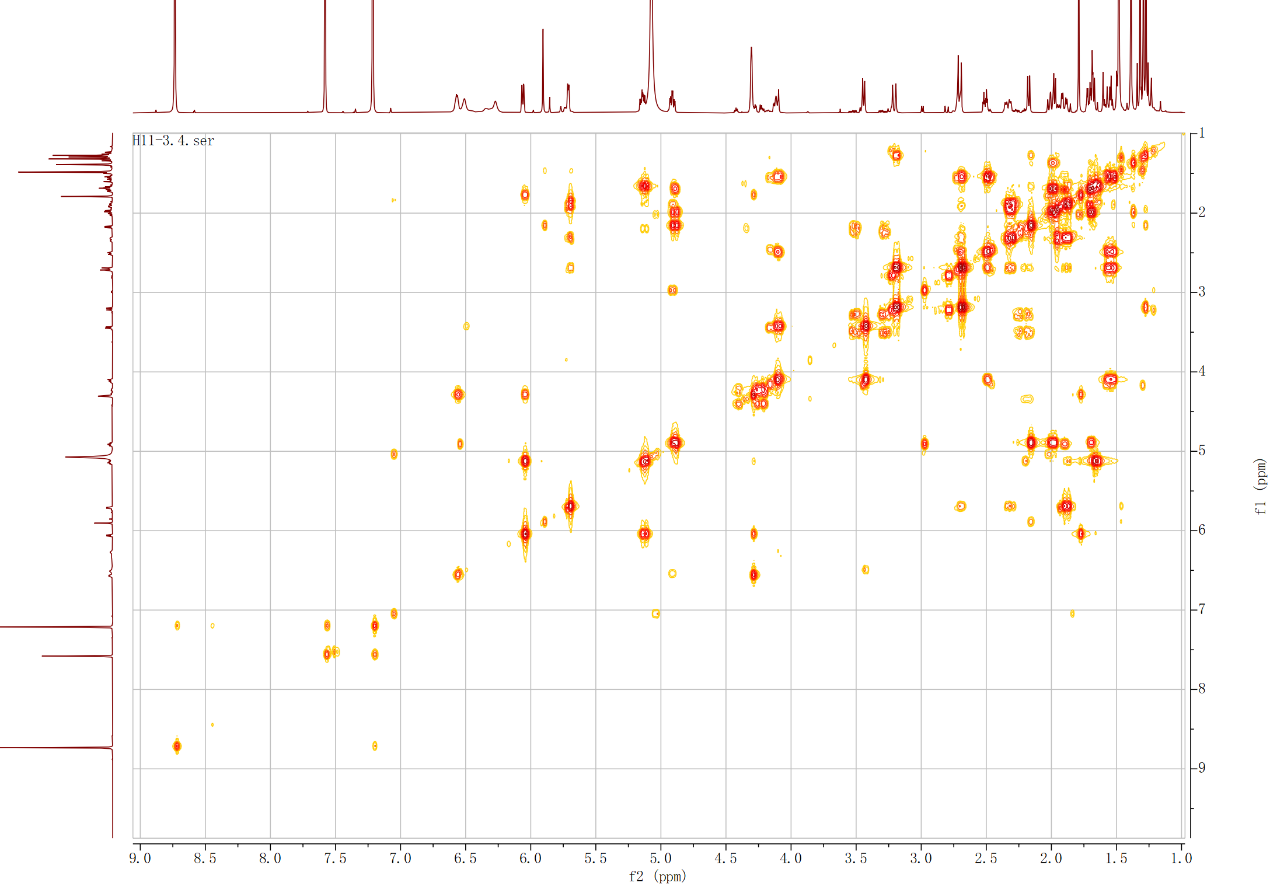


Figure S59. ^1^H-^1^H COSY spectrum (Pyrdine-D_5_) of **6**


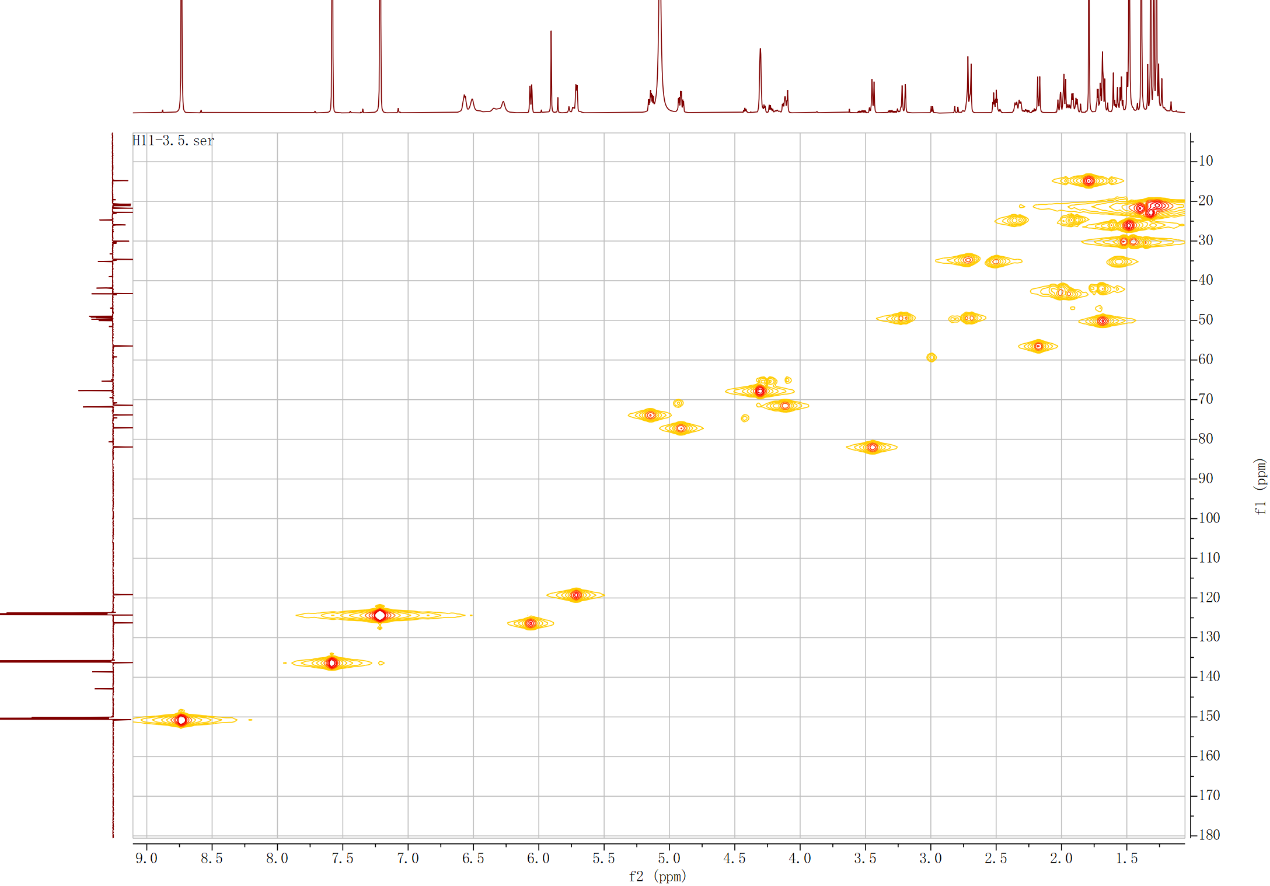


Figure S60. HSQC spectrum (Pyrdine-D_5_) of **6**


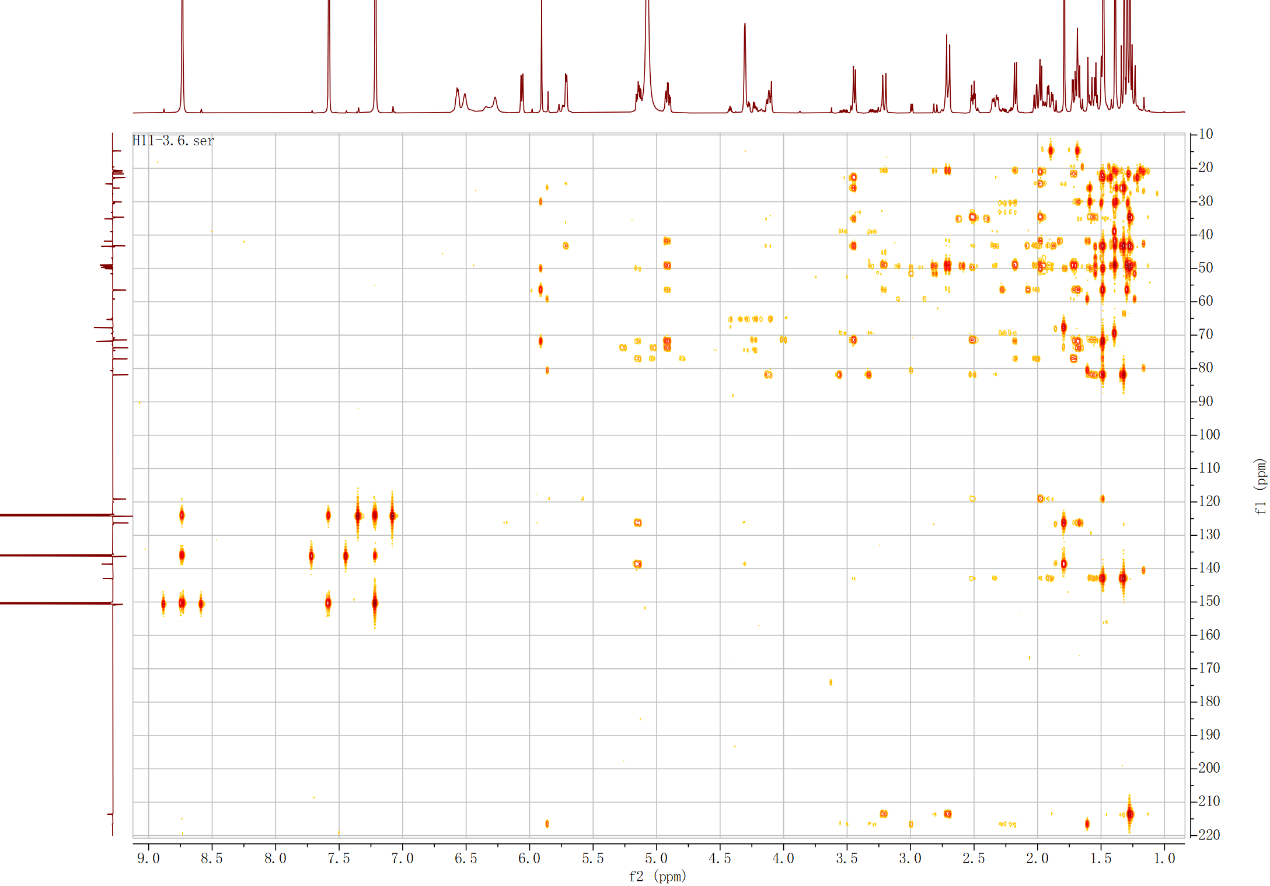


Figure S61. HMBC spectrum (Pyrdine-D_5_) of **6**


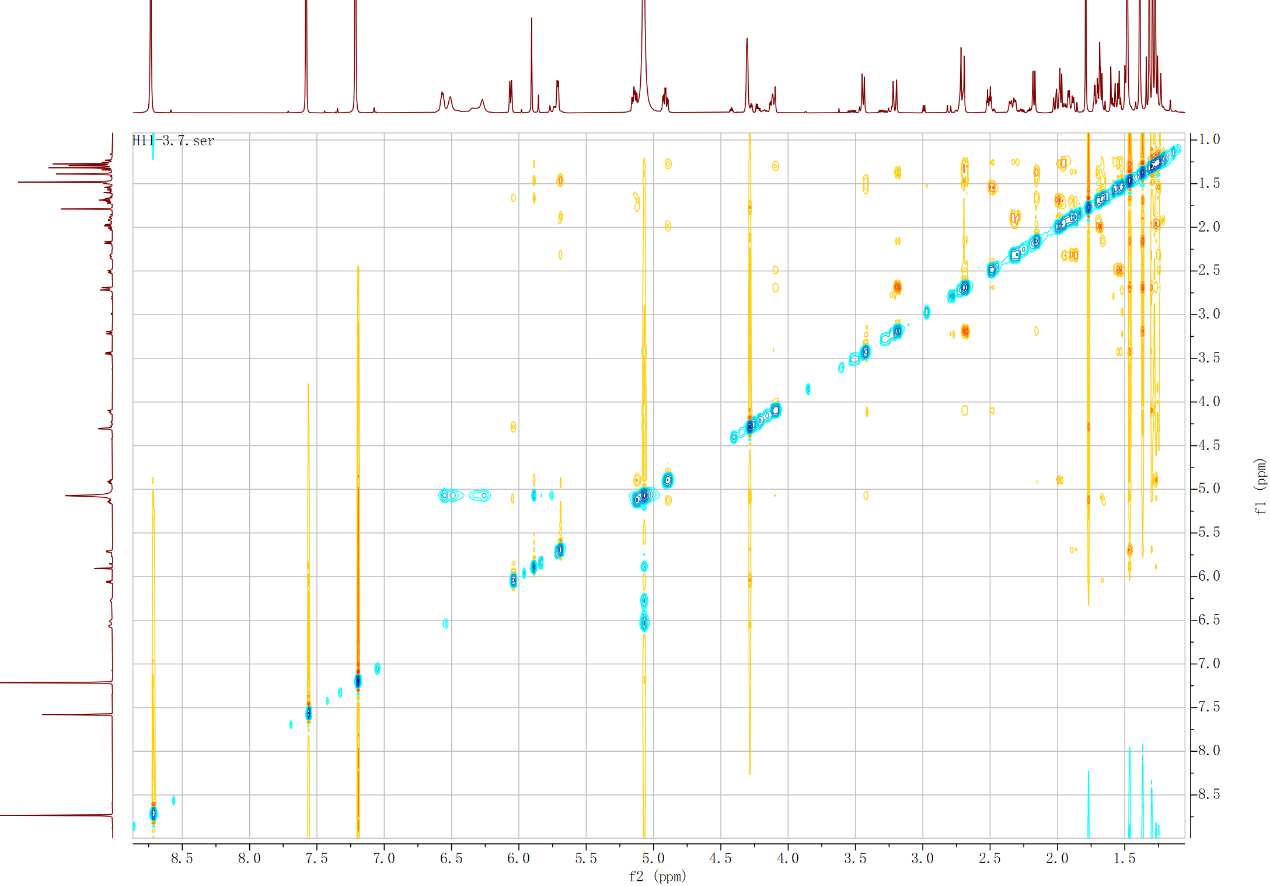


Figure S62. NOESY spectrum (Pyrdine-D_5_) of **6**

**S-1 Calculation of ^13^C NMR Spectra for 1.**

NMR shielding constants were calculated using the GIAO method at the mPW1PW91/6-311+G** level in gas phase by the GAUSSIAN09 program. Gibbs free energies for conformers were determined by using thermal correction at B3LYP-D3(BJ)/6-31G* level and electronic energies evaluated at the wB97M-V/def2-TZVP level in gas phase using ORCA. Boltzmann weights were computed using relative gibbs free energies. The unscaled chemical shifts (δ_u_) were computed using TMS (Tetramethylsilane) as a reference standard according to δ_u_ = σ_0_ - σ_x_, where σ_x_ is the Boltzmann averaged shielding tensor (over all significantly populated conformations) and σ_0_ is the shielding tensor of the TMS computed at the same level of theory employed for σ_x_. The scaled chemical shifts (δ_s_) were calculated as δ_s_ = (δ_u_ - b) / m, where m and b are the slope and intercept, respectively, deduced from a linear regression calculation on a plot of δ_u_ against δ_ex._
